# RNA Sequencing Provides Novel Insights into the Transcriptome of Aldosterone Producing Adenomas

**DOI:** 10.1038/s41598-019-41525-2

**Published:** 2019-04-18

**Authors:** Samuel Backman, Tobias Åkerström, Rajani Maharjan, Kenko Cupisti, Holger S. Willenberg, Per Hellman, Peyman Björklund

**Affiliations:** 1 Department of Surgical Sciences, Uppsala University, Uppsala, Sweden; 2 Department of Surgery, Marien-Hospital, Euskirchen, Germany; 3 Division of Endocrinology and Metabolism, Rostock University Medical Center, Rostock, Germany

**Keywords:** Gene expression, Adrenal gland diseases

## Abstract

Aldosterone producing adenomas (APAs) occur in the adrenal glands of around 30% of patients with primary aldosteronism, the most common form of secondary hypertension. Somatic mutations in *KCNJ5*, *ATP1A1*, *ATP2B3*, *CACNA1D* and *CTNNB1* have been described in ~60% of these tumours. We subjected 15 aldosterone producing adenomas (13 with known mutations and two without) to RNA Sequencing and Whole Genome Sequencing (n = 2). All known mutations were detected in the RNA-Seq reads, and mutations in *ATP2B3* (G123R) and *CACNA1D* (S410L) were discovered in the tumours without known mutations. Adenomas with *CTNNB1* mutations showed a large number of differentially expressed genes (1360 compared to 106 and 75 for *KCNJ5* and *ATP1A1*/*ATP2B3* respectively) and clustered together in a hierarchical clustering analysis. RT-PCR in an extended cohort of 49 APAs confirmed higher expression of *AFF3* and *ISM1* in APAs with *CTNNB1* mutations. Investigation of the expression of genes involved in proliferation and apoptosis revealed subtle differences between tumours with and without *CTNNB1* mutations. Together our results consolidate the notion that *CTNNB1* mutations characterize a distinct subgroup of APAs.

## Introduction

Primary aldosteronism (PA) is a common form of secondary hypertension and consists of inappropriate secretion of aldosterone from the adrenal glands. Once considered a rare entity, it is now thought to affect five to ten percent of the hypertensive population^1^. Aldosterone producing adenomas (APAs) represent a subgroup of PA that is potentially curable by surgical removal of affected adrenal gland. Since 2011 recurrent mutations have been identified in several genes in APAs: *KCNJ5*^2^, *CACNA1D*^3,4^, *ATP1A1*^3,5^, *ATP2B3*^5^, and *CTNNB1*^6,7^, cumulatively accounting for 50–60% of all APAs. Additionally, rare mutations have been reported in *GNAS*^8^, *PRKACA*^9^ and *CACNA1H*^10^. Mutations in *KCNJ5*, *CACNA1D*, *ATP1A1* and *ATP2B3* alter intracellular ion homeostasis, leading to depolarization and autonomous aldosterone hypersecretion. It is as of now uncertain whether these mutations are sufficient to cause adenoma development. A recent case report of an APA with a *KCNJ5* mutation in a patient with Gardner syndrome on the basis of a germline mutation in *APC* highlights the potential for a two-hit model of APA tumorigenesis with a first genetic hit leading to adenoma development, followed by a second hit in one of the mentioned genes leading to autonomous aldosterone secretion^11^. Conversely, the adrenocortical hyperplasia seen in patients with germline mutations in *KCNJ5* (Familial hyperaldosteronism type III) suggests that these mutations do indeed stimulate proliferation. On the contrary, mutations in *CTNNB1* are known to cause cell proliferation and have been demonstrated to promote tumour growth in different contexts. A previous study from our group reported a significantly larger size of APAs carrying *CTNNB1* mutations than APAs lacking mutations in this gene^6^, suggesting that hyperaldosteronism in these cases may be caused primarily by the sheer number of secreting cells rather than oversecretion by the individual cells. One case series reported an association between *CTNNB1* mutations and aberrant hormone receptor expression and disease debut during pregnancy or menopause^7^. However, this pattern of disease debut has not been seen in other cohorts^12,13^.

Several studies have reported on gene expression patterns in APAs. One study identified higher expression of the zona glomerulosa-associated gene *NPNT* in *ATPase*-/*CACNA1D* -mutated tumours compared to *KCNJ5*-mutated tumours^3^. This result has since been validated in a separate cohort^14^, and its functional importance elucidated^15^, but another large study including 102 tumours did not detect a distinct transcriptional profile in *KCNJ5*-mutated APAs^16^. To our knowledge no studies to date have reported on transcriptional differences between *CTNNB1*-mutated APAs and APAs harbouring other mutations. The proteome and the transcriptome of normal adrenal tissue has been thoroughly characterized^17^. In this paper we use mRNA sequencing of 15 well-characterized samples (including two without known mutation) to further investigate the transcriptional landscape of aldosterone producing adenomas with special emphasis on tumours with and without *CTNNB1* mutations, as well as whole genome sequencing of two of the APAs without known mutations despite targeted resequencing of the affected genes.

## Methods

### Cohort

We selected 15 tumours from our well-characterized cohort, which have been screened for mutations in the established recurrently mutated genes as previously reported^6,14,18^. Five tumours were known to carry *KCNJ5*-mutations, three to carry *ATP1A1*-mutations, two to carry *ATP2B3*-mutations and three were known to carry *CTNNB1*-mutations. Two tumours did not harbour known mutations despite screening by Sanger sequencing. The tumours were snap frozen in liquid nitrogen after adrenalectomy, and stored at −70 °C. Ethical approval was obtained from the Regional Ethical Review Board in Uppsala and the study was carried out in accordance with applicable ethical regulations. Included patients provided informed consent.

### Next Generation Sequencing

Serial sections were cut using a cryostat, and DNA/RNA was extracted from the sections using AllPrep DNA/RNA Kit (Qiagen Inc., Hilden, Germany) in accordance with the manufacturer’s instructions. The RNA was subjected to rRNA depletion and conversion to cDNA. Finally, libraries for RNA sequencing were prepared and sequenced on an Illumina HiSeq2500 at the SNP&SEQ platform at the Science for Life Laboratory, Uppsala, Sweden. Two of the samples without known mutations were subjected to Whole Genome Sequencing (with a target average read depth of 30x), along with constitutional DNA extracted from leukocytes from one of the two patients.

### RT-qPCR

RNA was converted into cDNA using First-Strand cDNA Synthesis kit (Fermentas, Thermo Fisher Scientific, Waltham, USA). RT-qPCR reactions were run in triplicates on a Bio-Rad CFX96 Real Time PCR Detection System using Bio-Rad SsoAdvanced Universal Sybr Green Supermix. *ACTB* was used as a reference gene. The primers used were: *AFF3* (5′-AGGCCAAGCTCTCCAAGTTC-3′, 5′-ACACAGCTGTTGGTTTCTCCA-3′), *ISM1* (5′-CCACCGAAGTGAGTCTGCTT-3′, 5′-CTCGCTTTTGCAGCTCATCC-3′), *ACTB* (5′-TCATGAAGTGTGACGTGGACATC-3′, 5′-CAGGAGGAGCAATGATCTTGATCT-3′). After an initial denaturation step (95 °C for 30 s), 40 cycles of 95 °C for 5 s and 58 °C for 7 s (*AFF3*, *ISM1*) or 12 s (*ACTB*) were executed. Melting curve analysis was performed after amplification. The 2^−ΔΔCt^ method was used to calculate relative expression. The two-sample Wilcoxon rank sum test with continuity correction was used for statistical analysis.

### Sanger sequencing

Targeted regions were amplified from extracted DNA by PCR. The primer sequences and PCRconditions are available in Supplementary Table 1. The PCR products were purified and sequenced at Beckman Coulter Genomics. Generated chromatograms were imported into CodonCode Aligner (CodonCode Corporation, Centerville, MA), aligned to the reference sequence and analysed.

### Expression analysis

The resulting FASTQ-files were analysed using Kallisto v. 0.43.0^19^ using the default settings except for the number of bootstraps per sample, which was set to 100. Downstream expression analyses were performed using Sleuth v. 0.29.0^20^. For gene level analyses, transcripts were aggregated by gene name. A false discovery rate of 0.1 was used for the expression analyses throughout this manuscript. The reference transcriptome was generated from the Ensemble release 87 cDNA FASTA file. Q-values reported in the manuscript refer to corrected p-values obtained from sleuth using the Wald test.

### Variant calling from whole genome sequencing data

The generated reads from the two investigated samples were aligned to the reference genome human_g1k_37 using Burrow-Wheeler Alignment (BWA) v. 0.7.12^21^. Subsequently, duplicate reads were marked (Picard) and filtered, and base quality score recalibration was performed using GATK^22^. For the sample with matched constitutional DNA, somatic variants were called using MuTect v. 1.1.5^23^. For the sample without matched non-tumoural DNA, variants were called using HaplotypeCaller. Potential functional impact of the variants was assessed using SIFT^24^ and PolyPhen-2^25^. Sequence conservation was assessed by multiple sequence alignment against the sequences of a range of species using Clustal Omega^26,27^.

### Variant calling from RNA-Sequencing data

Variants were called from the RNA-Sequencing data using the GATK Best Practices workflow for detection of germline variants in RNA-Seq data, as no such protocol for detection of somatic variants has been described to date to the best of our knowledge. Briefly, reads were aligned to the reference genome using the STAR 2-pass method^28^. Duplicates were marked and reads were sorted using Picard. GATK was run with the Split’n’Trim and ReassignMappingQuality tools, followed by local realignment around known insertions/deletions and Base Quality Score Recalibration. Subsequently, variants were called with HaplotypeCaller using the default settings. Generated variants were filtered for quality, and finally annotated using snpEff^29^.

### Gene ontology enrichment analysis

Gene Ontology (GO) enrichment analysis was performed using the Gene Ontology Consortium Enrichment analysis tool (http://www.geneontology.org), PANTHER Overrepresentation Test (release 20171205). Multiple testing was adjusted for using the Bonferroni method.

### Hierarchical clustering and principal components analysis

The dispersion index of every transcript in the cohort was calculated as D = s^2^/$\bar{{\rm{x}}}$ where s^2^ is the variance and $\bar{{\rm{x}}}$ is the mean expression level. The 3000 transcripts with the highest dispersion index were selected and hierarchical clustering was performed and a heatmap was generated using the *heatmap*.*2* function in the R package *gplots*. For visualization purposes all expression values were log2-transformed with an offset of +1 prior to generating the heatmap. The Principal Components Analysis (PCA) was performed using the *sleuth* R package.

## Results

### Cohort

An overview of the cohort is presented in Table 1. Five patients were male and 10 were female. The average age at surgery was 52 years and the average tumour diameter was 16 mm.

**Table 1:** Mutations in the studied tumours and details on their discovery and RNA Seq validation.

Case	Sex	Age	Size	Mutation	Means of discovery	Mutant reads/Total reads in RNA Seq
APA-28	F	32	16	KCNJ5 Leu168Arg	Sanger sequencing	738/1489
APA-31	F	48	15	KCNJ5 Leu168Arg	Sanger sequencing	716/1269
APA-29	F	51	12	KCNJ5 Leu168Arg	Sanger sequencing	610/1270
APA-30	F	45	19	KCNJ5 Gly151Arg	Sanger sequencing	673/1355
APA-39	F	55	15	KCNJ5 Gly151Arg	Sanger sequencing	1116/2008
APA-37	F	55	13	CTNNB1 Ser45Pro	Sanger sequencing	2128/4036
APA-273	F	59	21	CTNNB1 Ser45Pro	Sanger sequencing	779/1733
APA-114	F	26	39	CTNNB1 Ser45Phe	Sanger sequencing	1541/3270
APA-45	M	65	13	CACNA1D Ser410Leu	WGS*	103/124
APA-44	M	56	10	ATP2B3 Leu425_Val426del	Sanger sequencing	73/75
APA-35	F	63	10	ATP2B3 Leu425_Val426del	Sanger sequencing	129/129
APA-13	M	39	10	ATP2B3 Gly123Arg	WGS*	315/322
APA-42	M	62	13	ATP1A1 Val332Gly	Sanger sequencing	1027/2299
APA-19	M	57	23	ATP1A1 Met102_Trp105del	Sanger sequencing	175/1443
APA-53	F	60	12	ATP1A1 Leu104Arg	Sanger sequencing	395/827

Thirteen of 15 mutations have been previously reported and * denotes a mutation not previously reported.

### Mutations in ATP2B3 and CACNA1D discovered by Whole Genome Sequencing

A novel mutation in *ATP2B3* (p.G123R) and a previously described mutation in *CACNA1D* (p.S410L) were detected in two tumours that had previously been screened for mutations in these genes, but which were previously undetected. Both mutations affected conserved residues (Supplementary Fig. 1) and were classified as “deleterious” by SIFT and “probably damaging” by PolyPhen-2. MuTect classified the *CACNA1D* mutation as somatic. Both the reference and the mutated allele of the *ATP2B3*-mutation were found in both WGS and RNA-Seq reads from the affected sample, which originated from a man, suggesting that the mutation is somatic as the gene is located on the X chromosome. Both mutations were verified by Sanger sequencing (Supplementary Fig. 2).

### Mutation detection in RNA sequencing reads

All of the 15 mutations identified by DNA sequencing could be identified in the RNA-Sequencing data (Table 1).

### Differential expression between mutational groups

**Figure 1:**
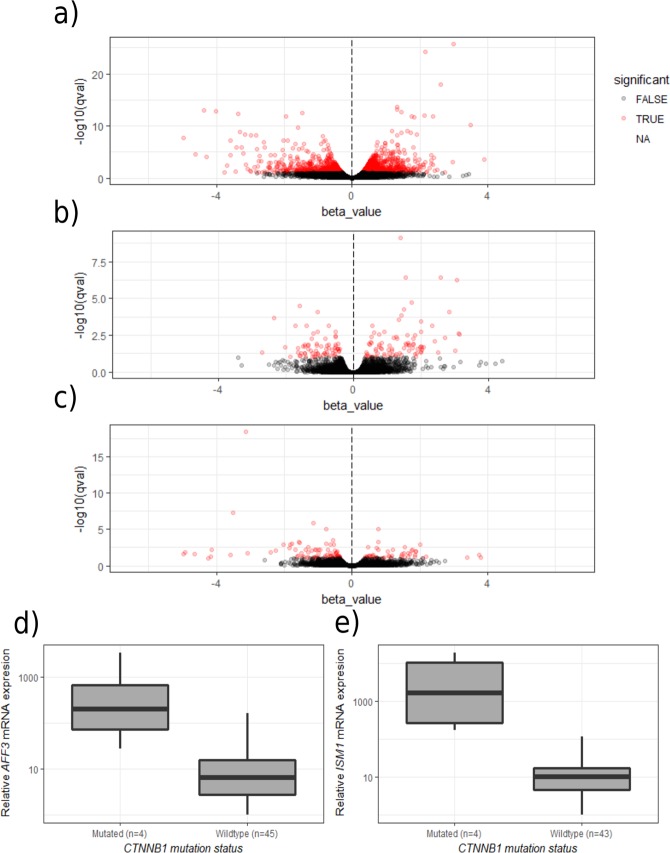
Volcano plots for (**a**) *CTNNB1*-mutated tumours vs. *CTNNB1*-wildtype tumours (**b**) *KCNJ5*-mutated tumours vs. *KCNJ5*-wildtype tumours (**c**) *ATP1A1*/*ATP2B3*-mutated tumours vs. *ATP1A1*/*ATP2B3*-wildtype tumours. (**d**) Overexpression of *AFF3* in *CTNNB1* mutated tumours confirmed by RT-qPCR (p < 0.01 by Wilcoxon’s rank sum test). (**e**) Overexpression of *ISM1* in *CTNNB1* mutated tumours confirmed by RT-qPCR (p < 0.01 by Wilcoxon’s rank sum test). Note that “wildtype” refers to tumours that are wildtype for the gene in question, and that these tumours may carry mutations in other genes.

We defined three mutational subgroups: *KCNJ5*-mutated tumours, *ATPase*-mutated tumours and *CTNNB1*-mutated tumours. For each of these subgroups we performed a differential expression analysis between all the tumours in the group and all the tumours not in the group (i.e. the tumours with mutations in other genes, Fig. 1a–c). This analysis revealed that 1360 genes were differentially expressed between tumours with *CTNNB1*-mutations and tumours without *CTNNB1*-mutations. Several genes previously found overexpressed in other (non-APA) types of *CTNNB1*-mutated adrenal tumours were overexpressed in *CTNNB1-*mutated APAs: *AFF3*, *ISM1*, *NKD1*, *ENC1* and *RALBP1*. *AFF3* and *ISM1* were selected for validation by RT-qPCR, and their overexpression confirmed (Fig. 1d,e). Gene ontology enrichment analysis of transcripts differentially expressed in *CTNNB1*-mutated tumours shows overrepresentation of genes involved in ribosome and protein synthesis and protein trafficking pathways (Table 2, Supplementary Table 2). In contrast, only 106 genes were differentially expressed between tumours carrying *KCNJ5*-mutations and those without *KCNJ5*-mutations, and 75 genes between tumours with *ATPase* mutations and those without (Supplementary Tables 3–5). When *CTNNB1*-mutated tumours were excluded, 40 genes were found differentially expressed between tumours with *KCNJ5* mutations and tumours with *ATPase* or *CACNA1D* mutations (Supplementary Table 6). Of these, 13 were higher expressed in *KCNJ5*-mutated tumours and 27 had higher expression in the *ATPase/CACNA1D* group.

**Table 2:** Top 10 GO terms enriched in the set of genes differentially expressed between CTNNB1 wildtype and CTNNB1 mutated tumours.

GO term	Fold Enrichment	p-value (FDR)
SRP-dependent cotranslational protein targeting to membrane (GO:0006614)	9.29	2.32E-25
cotranslational protein targeting to membrane (GO:0006613)	8.88	1.96E-25
protein targeting to ER (GO:0045047)	8.62	4.12E-25
establishment of protein localization to endoplasmic reticulum (GO:0072599)	8.30	1.36E-24
viral transcription (GO:0019083)	7.58	4.32E-23
nuclear-transcribed mRNA catabolic process, nonsense-mediated decay (GO:0000184)	7.52	8.54E-24
ribosomal small subunit assembly (GO:0000028)	7.48	1.19E-03
protein localization to endoplasmic reticulum (GO:0070972)	7.35	3.37E-24
viral gene expression (GO:0019080)	7.24	5.71E-24
protein targeting to membrane (GO:0006612)	6.97	2.24E-23

The full set is reported in Supplementary Table 6.

### Hierarchical clustering

**Figure 2:**
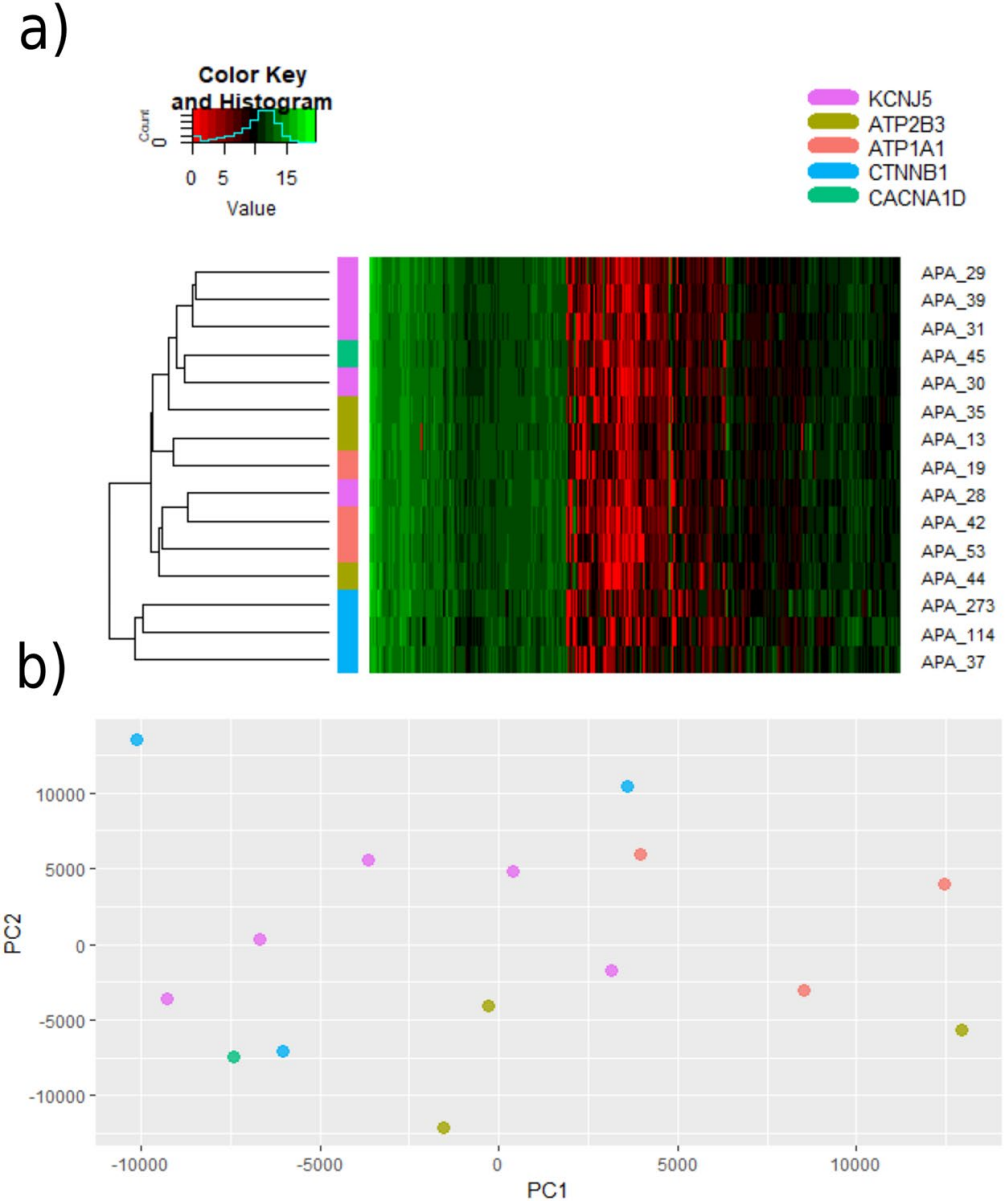
(**a**) Unsupervised hierarchical clustering based on the 3000 transcripts with the highest variability in the cohort. Two clusters are seen, characterized by the presence or absence of *CTNNB1*-mutations. (**b**) Principal components analysis of the included tumours.

Hierarchical clustering based on the expression values of the 3000 most variably expressed transcripts identified two clusters, characterized by the presence or absence of *CTNNB1*-mutations (Fig. 2a). However, visual inspection of the heatmap suggests that the overall transcriptional differences are subtle. A principal components analysis of the tumours shows no clear separation of the tumours carrying different mutations (Fig. 2b).

### Aberrant hormone receptor expression

**Figure 3:**
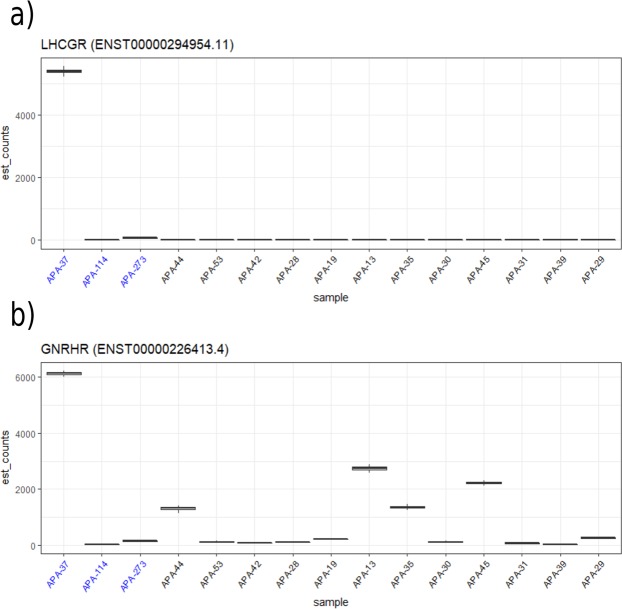
APA-37, which harbours a mutation in *CTNNB1*, has a higher expression of (**a**) *LHCGR* and (**b**) *GNRHR* than all other tumours in the cohort. *CTNNB1*-mutated samples are denoted in blue.

One tumour with a *CTNNB1*-mutation was found to have substantially higher expression of both *GNRHR* and *LHCGR* than the other studied tumours (Fig. 3). The female patient from whom this sample originated was diagnosed with hypertension at age 24 and with primary aldosteronism at age 54. Adrenalectomy was performed at age 55 after workup with computed tomography and adrenal venous sampling. The pathology report suggested micronodular hyperplasia with two dominant nodules. The onset of hypertension preceded her first pregnancy, and while her hypertension persisted throughout her two pregnancies, she did not require increased doses of antihypertensive medications while pregnant.

### Investigation of APA related pathways

**Figure 4:**
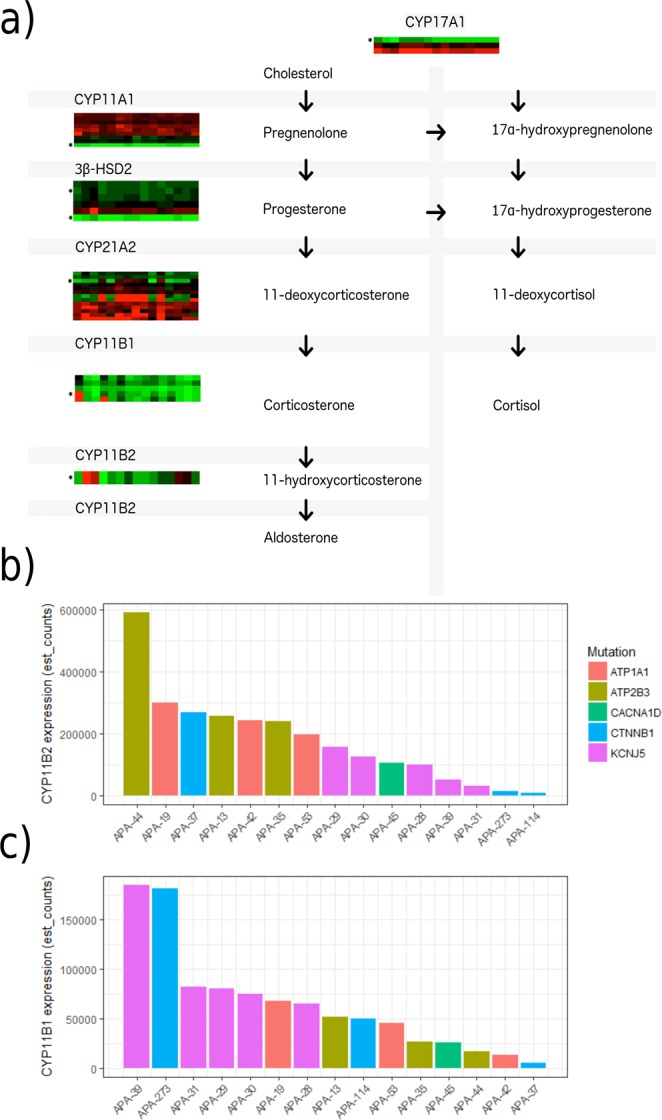
(**a**) Expression of aldosterone synthesis genes in aldosterone producing adenomas. Each heatmap represents the 15 studied tumours (horizontally) and the detected transcripts for each gene (vertically). Principal transcripts are annotated with an asterisk. (**b**) Expression of the *CYP11B2* transcript, encoding aldosterone synthase, in the studied tumours. (**c**) Expression of *CYP11B1* in the studied tumours.

We further specifically investigated three pathways: aldosterone and cortisol biosynthesis, apoptosis and cell division. The dominating *CYP11A1* transcript was shared between all tumours and corresponded to the APPRIS P1 transcript. On average it contributed to more than 97% of the *CYP11A1* RNA expression. The same was true for *CYP17A1* where the dominant transcript contributed on average >99% of all expression and for *HSD3B2* (>95%). Both *CYP21A2* and *CYP11B1* had discrepant dominant transcripts between the tumours, mirrored by a great range of total expression of these genes in the cohort, with some samples having very high expression and others very low (Fig. 4a). The single *CYP11B2*-transcript annotated in Ensembl was expressed in all samples but varied greatly in expression level with the highest expressing samples having an expression 89 times higher than the lowest expressing sample (Fig. 4b). While not statistically significant, *ATPase* and *CACNA1D*-mutated tumours generally had higher expression of *CYP11B2* than *KCNJ5*-mutated tumours while the opposite trend was seen for *CYP11B1*. The expression of *CYP11B1* and *CYP11B*2 in *CTNNB1*-mutated tumours varied greatly.

**Figure 5:**
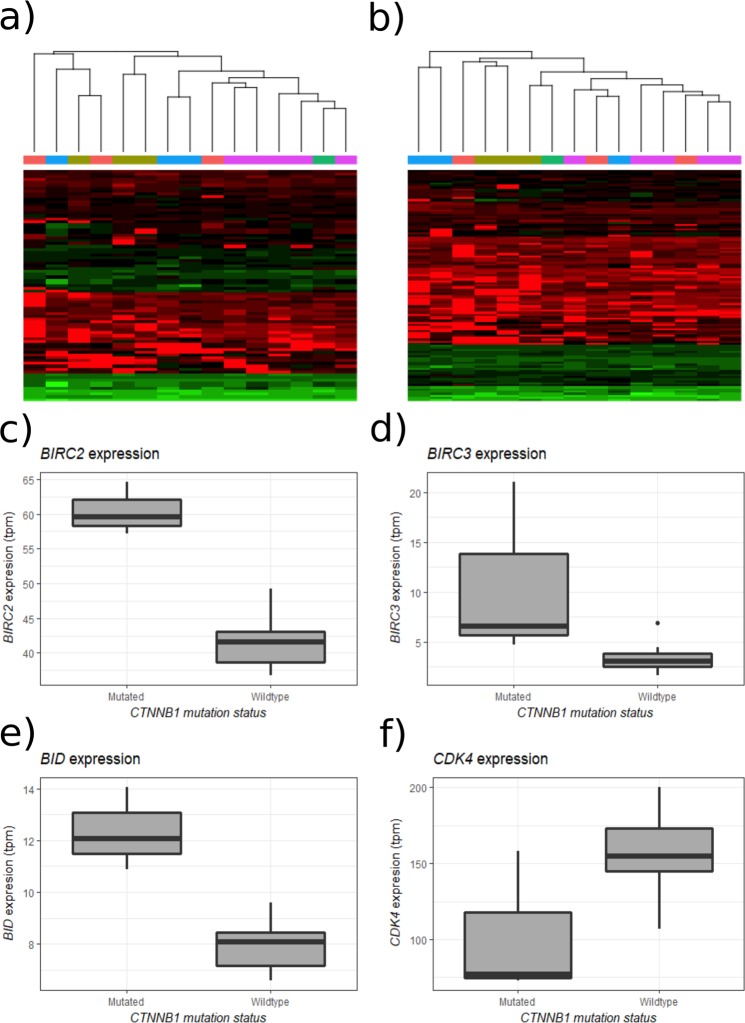
Hierarchical clustering of tumours based on genes involved in (**a**) apoptosis and (**b**) proliferation. Expression of BIRC*2* (**c**), *BIRC3* (**d**) and BID (**e**) is higher in *CTNNB1*-mutated tumours. (**f**) Expression of *CDK4* is lower in *CTNNB1*-mutated tumours. Tumours labelled “wildtype” lack mutations in *CTNNB1* but carry mutations in other genes.

Fifteen genes selected for their involvement in the regulation or execution of apoptosis were selected and analysed in detail. Hierarchical clustering based on the 83 associated transcripts revealed no obvious grouping of tumours (Fig. 5a). Three of these genes, apoptosis inhibitors *BID*, *BIRC2* and *BIRC3* were found in the differential expression analyses, being overexpressed in *CTNNB1*-mutated tumours. (Fig. 5c–e). Similarly, 36 genes involved in regulating the cell cycle were selected. Again, no obvious grouping was revealed upon hierarchical clustering of the tumours (Fig. 5b). Of these genes, only *CDK4* occurred in the differential expression analyses, being underexpressed in *CTNNB1*-mutated tumours (Fig. 5f). None of the genes in either of the analyses were differentially expressed in *KCNJ5*- or *ATPase*-mutated tumours. Taken together, these findings suggest a role for altered cell proliferation in *CTNNB1*-mutated APAs.

## Discussion

We describe two mutations in known genes in aldosterone producing adenomas that were previously reported as wildtype for the canonical genes after hot-spot Sanger sequencing. The detected S410L mutation in *CACNA1D* has been reported once before (in an aldosterone producing cell cluster)^30^, while the *ATP2B3* G123R to the best of our knowledge has not been described previously. Both mutations affect conserved residues. SIFT classifies the mutations as “deleterious” and PolyPhen2 classifies them as “probably damaging”, supporting their pathogenicity. All known mutations in this cohort were detected and validated in the mRNA sequencing data. The high expression of the canonical mutated APA genes in APAs renders RNA sequencing a sensitive method for mutation detection in these tumours.

For *CACNA1D* and *ATP2B3* we previously sequenced only the hotspot regions, not including the regions with the two mutations found by WGS. The two mutations detected by WGS occur in relatively high frequency in the reads (83%/97% and 83%/31% in RNA seq and WGS reads respectively). Sanger sequencing using new primers detected the mutations in *CACNA1D* and *ATP2B3*. The identification of mutations in the known genes in both included “wildtype” tumours suggests that mutations in the known driver genes may contribute to the pathogenesis of a larger fraction of APAs than thought.

Based on transcriptome analyses, tumours carrying *CTNNB1*-mutations appear as distinct subset of APAs. They cluster separately in our unsupervised analysis and have a substantial number of transcripts that are differentially expressed compared to other tumours. This is not surprising given the role of β-catenin in activating the LEF and TCF transcription factors, likely more directly affecting other transcriptional patterns than the other genes mutated in APAs. Several of the genes upregulated in *CTNNB1*-mutated tumours have previously been described as overexpressed in *CTNNB1*-mutated adrenal lesions, regardless of type of hormone production or differentiation level: *AFF3*, *ISM1*, *NKD1*, *ENC1* and *RALBP1*^31,32^. Of these, *AFF3* has been demonstrated to affect cell proliferation and apoptosis in an adrenocortical carcinoma cell line, and to predict outcome in two ACC patient cohorts^31^. Gene ontology analysis revealed that many of the genes differentially expressed in *CTNNB1*-mutated APAs are involved in pathways related to protein synthesis and shuttling. The targeted investigation of cell cycle and apoptosis genes revealed few and subtle differences between the subgroups. Of note all significant differences in expression were between *CTNNB1* mutated and *CTNNB1* wildtype tumours. This supports the notion that *CTNNB1* mutations in APAs drive proliferation and tumour growth.

One of the *CTNNB1*-mutated samples showed remarkably high expression of hormone receptors *LHCGR* and *GNRHR*. The patient was diagnosed with hypertension prior to her first pregnancy and does not report requiring increased doses of antihypertensive medication during her pregnancies. The significance of the hormone receptor overexpression in this particular case remains unknown.

Previous studies have reported discordant data regarding the association of *CYP11B1*/*CYP11B2* expression with mutational status. Azizan *et al*. reported higher expression of *CYP11B1* and a trend towards lower expression of *CYP11B2* in *KCNJ5*-mutated APAs and we previously reported lower expression of *CYP11B2* in *KCNJ5*-mutated tumours than in *ATPase*-mutated tumours. On the contrary, Fernandes-Rosa *et al*. did not detect a dependence of *CYP11B1*/*CYP11B2*-expression on genotype. In the fifteen tumours subjected to RNA-Sequencing in this study, opposite patterns of expression in tumours with mutations in *KCNJ5* and those with mutations in *CACNA1D*/*ATP1A1*/*ATP2B3* were observed, with *KCNJ5*-mutants showing higher levels of *CYP11B1* expression and lower levels of *CYP11B2* expression. However, this pattern did not reach transcriptome-wide statistical significance and the variation within each genotype was pronounced. The expression in the *CTNNB1* mutated samples was variable with one sample showing high *CYP11B2* and low *CYP11B1* expression, and two samples showing low *CYP11B2* expression and variable *CYP11B1* expression. However, the small number of tumours (and in particular with *CTNNB1* mutations) means that these results should be considered with caution. Nevertheless, these results are concordant with previous suggestions that *KCNJ5*-mutated tumours co-secrete cortisol and aldosterone.

The main limitation of this study is the low number of included tumours, in particular with *CTNNB1*-mutation. Nevertheless, we were able to identify differentially expressed genes previously reported in other types of adrenal tumours with *CTNNB1*-mtations, and validated two of these using RT-PCR. An additional limitation is the inability to identify novel transcripts that comes with the pseudo-alignment based workflow employed in the analyses. However, the workflow has been demonstrated to be highly accurate for quantification of known transcripts and robustly detects differential expression even on the isoform level^19,20^. Moreover, it is established that fusion genes are infrequent in solid tumours compared to haematological malignancies. It is particularly unlikely that fusion genes would occur in aldosterone producing adenomas, which are benign and which mostly have well-ordered genomes^2^ with no or few chromosomal aberrations with the potential to create novel fusion transcripts.

## Conclusion

Our results demonstrate that RNA Sequencing can be used to identify pathogenic mutations in aldosterone producing adenomas. Two tumours previously subjected to hot-spot sequencing of known genes were found to carry mutations in the canonical APA genes, suggesting that these may be mutated in a larger fraction of tumours than previously thought. Several lines of analysis identify APAs with activating mutations in *CTNNB1* as a subgroup with a distinct transcriptional profile, supporting the notion that they have a distinctly different biology compared to other APAs.

## Supplementary information

Supplementary Dataset 1

## Data Availability

The General Data Protection Regulation (GDPR) requires data processing agreements, and the public genomics archives available in Europe do not enter such agreements. The dataset will be deposited in a GDPR-compliant local EGA-node as soon as this service is available. Until then, the data is deposited on a secure Swedish server and has been assigned a DOI (10.17044/NBIS/G000007), but regulations by the service provider may make access technically restricted to PIs at Swedish organizations. Data access requests may be submitted to the Science for Life Laboratory Data Centre through the DOI link.

## References

[1] Funder JW (2016). The Management of Primary Aldosteronism: Case Detection, Diagnosis, and Treatment: An Endocrine Society Clinical Practice Guideline. J Clin Endocrinol Metab.

[2] Choi M (2011). K+ channel mutations in adrenal aldosterone-producing adenomas and hereditary hypertension. Science.

[3] Azizan EA (2013). Somatic mutations in ATP1A1 and CACNA1D underlie a common subtype of adrenal hypertension. Nat Genet.

[4] Scholl UI (2013). Somatic and germline CACNA1D calcium channel mutations in aldosterone-producing adenomas and primary aldosteronism. Nat Genet.

[5] Beuschlein F (2013). Somatic mutations in ATP1A1 and ATP2B3 lead to aldosterone-producing adenomas and secondary hypertension. Nat Genet.

[6] Akerstrom T (2016). Activating mutations in CTNNB1 in aldosterone producing adenomas. Sci Rep.

[7] Teo AE (2015). Pregnancy, Primary Aldosteronism, and Adrenal CTNNB1 Mutations. N Engl J Med.

[8] Nakajima Y (2016). GNAS mutations in adrenal aldosterone-producing adenomas. Endocrine journal.

[9] Rhayem Y (2016). PRKACA Somatic Mutations Are Rare Findings in Aldosterone-Producing Adenomas. The Journal of clinical endocrinology and metabolism.

[10] Daniil G (2016). CACNA1H Mutations Are Associated With Different Forms of Primary Aldosteronism. EBioMedicine.

[11] Vouillarmet J (2016). Aldosterone-Producing Adenoma With a Somatic KCNJ5 Mutation Revealing APC-Dependent Familial Adenomatous Polyposis. J Clin Endocrinol Metab.

[12] Berthon A, Drelon C, Val P (2016). Pregnancy, Primary Aldosteronism, and Somatic CTNNB1 Mutations. N Engl J Med.

[13] Murtha TD, Carling T, Scholl UI (2016). Pregnancy, Primary Aldosteronism, and Somatic CTNNB1 Mutations. N Engl J Med.

[14] Akerstrom T (2015). Novel somatic mutations and distinct molecular signature in aldosterone-producing adenomas. Endocr Relat Cancer.

[15] Teo AE (2017). Physiological and Pathological Roles in Human Adrenal of the Glomeruli-Defining Matrix Protein NPNT (Nephronectin). Hypertension.

[16] Boulkroun S (2012). Prevalence, clinical, and molecular correlates of KCNJ5 mutations in primary aldosteronism. Hypertension.

[17] Bergman, J. *et al*. The human adrenal gland proteome defined by transcriptomics and antibody-based profiling. *Endocrinology*, en20161758, 10.1210/en.2016-1758 (2016). 10.1210/en.2016-1758 27901589

[18] Akerstrom T (2012). Comprehensive re-sequencing of adrenal aldosterone producing lesions reveal three somatic mutations near the KCNJ5 potassium channel selectivity filter. PLoS One.

[19] Bray NL, Pimentel H, Melsted P, Pachter L (2016). Near-optimal probabilistic RNA-seq quantification. Nat Biotechnol.

[20] Pimentel H, Bray NL, Puente S, Melsted P, Pachter L (2017). Differential analysis of RNA-seq incorporating quantification uncertainty. Nature methods.

[21] Li H, Durbin R (2009). Fast and accurate short read alignment with Burrows-Wheeler transform. Bioinformatics.

[22] McKenna A (2010). The Genome Analysis Toolkit: a MapReduce framework for analyzing next-generation DNA sequencing data. Genome Res.

[23] Cibulskis K (2013). Sensitive detection of somatic point mutations in impure and heterogeneous cancer samples. Nat Biotechnol.

[24] Ng PC, Henikoff S (2001). Predicting deleterious amino acid substitutions. Genome Res.

[25] Adzhubei IA (2010). A method and server for predicting damaging missense mutations. Nat Methods.

[26] Li W (2015). The EMBL-EBI bioinformatics web and programmatic tools framework. Nucleic Acids Res.

[27] Sievers F (2011). Fast, scalable generation of high-quality protein multiple sequence alignments using Clustal Omega. Mol Syst Biol.

[28] Dobin A (2013). STAR: ultrafast universal RNA-seq aligner. Bioinformatics.

[29] Cingolani P (2012). A program for annotating and predicting the effects of single nucleotide polymorphisms, SnpEff: SNPs in the genome of Drosophila melanogaster strain w1118; iso-2; iso-3. Fly (Austin).

[30] Omata K (2017). Aldosterone-Producing Cell Clusters Frequently Harbor Somatic Mutations and Accumulate With Age in Normal Adrenals. Journal of the Endocrine Society.

[31] Lefevre L (2015). Combined transcriptome studies identify AFF3 as a mediator of the oncogenic effects of beta-catenin in adrenocortical carcinoma. Oncogenesis.

[32] Durand J, Lampron A, Mazzuco TL, Chapman A, Bourdeau I (2011). Characterization of differential gene expression in adrenocortical tumors harboring beta-catenin (CTNNB1) mutations. The Journal of clinical endocrinology and metabolism.

