# Supplementary material for: RNA Sequencing Provides Novel Insights into the Transcriptome of Aldosterone Producing Adenomas
Source: Sci Rep. 2019 Apr 18;9:6269. doi: 10.1038/s41598-019-41525-2 (PMC6472367; doi:10.1038/s41598-019-41525-2)
Supplement: Supplementary file 1 — Supplementary Dataset 1 [file 41598_2019_41525_MOESM1_ESM.pdf]

RNA Sequencing Provides Novel Insights Into the Transcriptome of Aldosterone Producing Adenomas

Supplementary figures and tables

Samuel Backman*[1], Tobias Åkerström*[1], Rajani Maharjan[1], Kenko Cupisti[2], Holger S Willenberg[3], Per Hellman[#1] and Peyman Björklund[#1].

1) Department of Surgical Sciences, Uppsala University, Uppsala, Sweden. 2) Department of Surgery, Marien-Hospital, Euskirchen, Germany. 3) Division of Endocrinology and Metabolism, Rostock University Medical Center, Rostock, Germany.

* These authors contributed equally to this study

# These authors contributed equally to this study

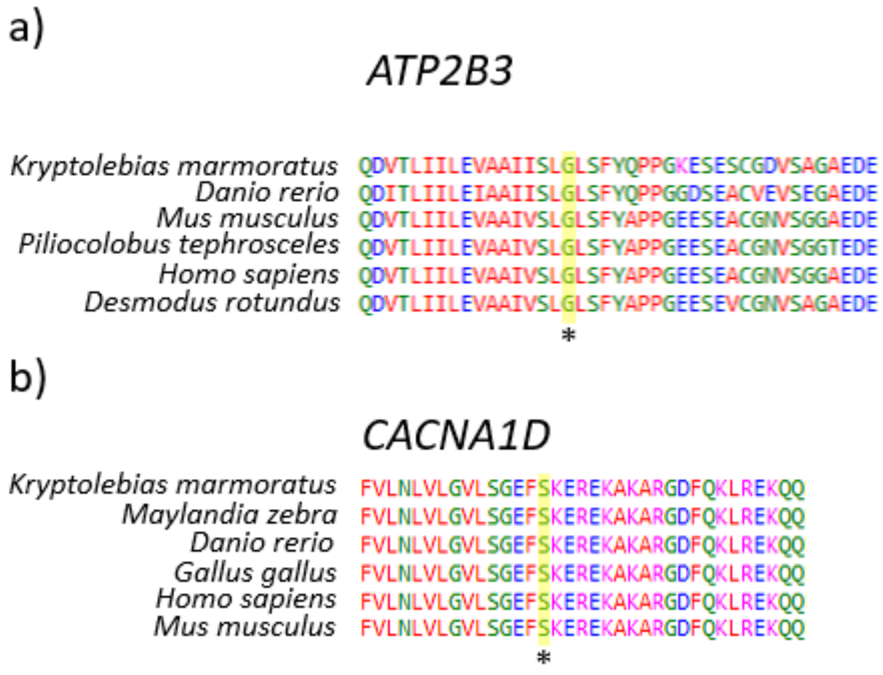

Supplementary Figure 1 A) Multiple sequence alignment of the ATP2B3-region mutated in APA-13. B) Multiple sequence alignment of the CACNA1D-region mutated in APA-45.

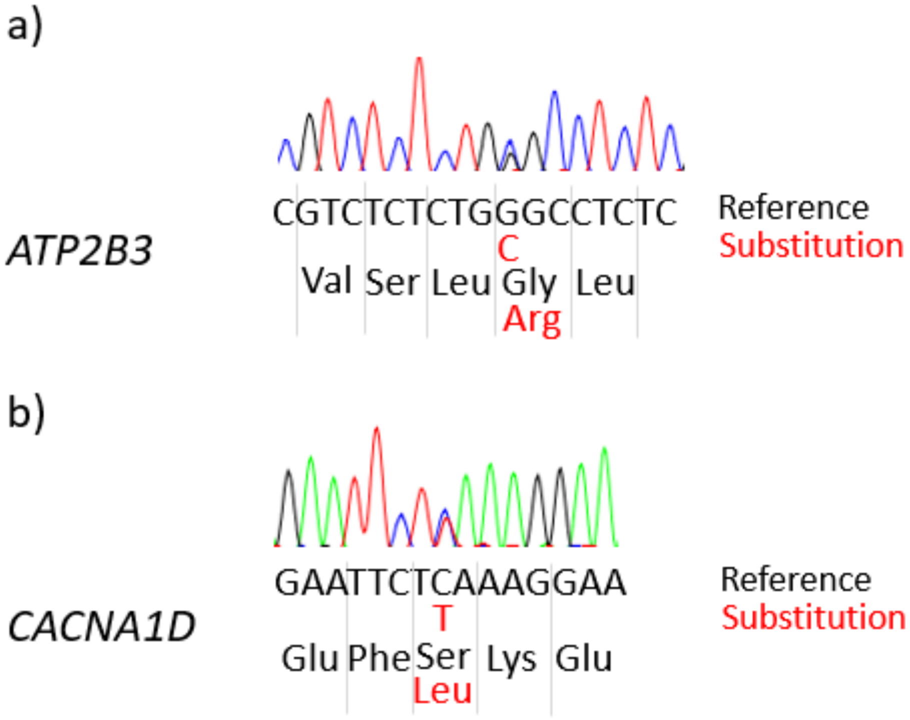

Supplementary Figure 2 A) The Gly123Arg mutation in ATP2B3 in APA-13 verified by Sanger sequencing. B) The Ser410Leu mutation in CACNA1D in APA-45 verified by Sanger sequencing.

Supplementary table 1: PCR Primers and conditions

**Experiment**	**Primer 1**	**Primer 2**	**Conditions**
ATP2B3 G123R validation	AGCAGTCTGCAGAAAGGCTC	GTTCCGGATGACCGTGAACT	Thermo Fisher Platinum Taq Polymerase. 35 ul reactions. 0.7 ul of each primer (primer stock is 10 pmol/ul), 0.7 ul dNTP (100 mM of each), 1.05 MgCl2. Annealing temperature 60°C. 0.7 ul template (8 ng/ul). The PCR products were purified and sequenced at Beckman Coulter Genomics
CACNA1D S410L validation	CTATACTCAGTGCTGTGTGTCCT	TAGGCGTCTGAACCGCACA	Thermo Fisher Platinum Taq Polymerase. 35 ul reactions. 0.7 ul of each primer (primer stock is 10 pmol/ul), 0.7 ul dNTP (100 mM of each), 1.05 MgCl2. Annealing temperature 60°C. 0.7 ul template (8 ng/ul). The PCR products were purified and sequenced at Beckman Coulter Genomics
ACTB qPCR	TCATGAAGTGTGACGTGGACATC	CAGGAGGAGCAATGATCTTGATCT	Initial denaturation at 95°C for 30s. 40 cycles of 95°C for 5 s - 58°C for 12s - plate read. Bio-Rad CFX96 Real Time PCR Detection System, Bio-Rad SsoAdvanced Universal Sybr Green Supermix.
AFF3 qPCR	AGGCCAAGCTCTCCAAGTTC	ACACAGCTGTTGGTTTCTCCA	Initial denaturation at 95°C for 30s. 40 cycles of 95°C for 5 s - 58°C for 7s - plate read. Bio-Rad CFX96 Real Time PCR Detection System, Bio-Rad SsoAdvanced Universal Sybr Green Supermix.
ISM1 qPCR	CCACCGAAGTGAGTCTGCTT	CTCGCTTTTGCAGCTCATCC	Initial denaturation at 95°C for 30s. 40 cycles of 95°C for 5 s - 58°C for 7s - plate read. Bio-Rad CFX96 Real Time PCR Detection System, Bio-Rad SsoAdvanced Universal Sybr Green Supermix.

Supplementary table 2: Gene ontology enrichment analysis of genes differentially expressed between CTNNB1-mutated and CTNNB1-wildtype tumors							
Analysis Type:	PANTHER Overrepresentation Test (Released 20171205)						
Annotation Version and Release Date:	GO Ontology database Released 2017-12-27						
Analyzed List:	upload_1 (Homo sapiens)						
Reference List:	Homo sapiens (all genes in database)						
Test Type:	FISHER						
**GO biological process complete**	**Homo sapiens - REFLIST (21042)**	**upload_1 (1340)**	**upload_1 (expected)**	**upload_1 (over/under)**	**upload_1 (fold Enrichment)**	**upload_1 (raw P-value)**	**upload_1 (FDR)**
SRP-dependent cotranslational protein targeting to membrane (GO:0006614)	93	55	5.92	+	9.29	1.50E-29	2.32E-25
cotranslational protein targeting to membrane (GO:0006613)	99	56	6.30	+	8.88	2.52E-29	1.96E-25
protein targeting to ER (GO:0045047)	102	56	6.50	+	8.62	7.96E-29	4.12E-25
establishment of protein localization to endoplasmic reticulum (GO:0072599)	106	56	6.75	+	8.30	3.51E-28	1.36E-24
viral transcription (GO:0019083)	114	55	7.26	+	7.58	3.34E-26	4.32E-23
nuclear-transcribed mRNA catabolic process, nonsense-mediated decay (GO:0000184)	119	57	7.58	+	7.52	5.50E-27	8.54E-24
ribosomal small subunit assembly (GO:0000028)	21	10	1.34	+	7.48	7.96E-06	1.19E-03
protein localization to endoplasmic reticulum (GO:0070972)	126	59	8.02	+	7.35	1.74E-27	3.37E-24
viral gene expression (GO:0019080)	128	59	8.15	+	7.24	3.31E-27	5.71E-24
protein targeting to membrane (GO:0006612)	133	59	8.47	+	6.97	1.59E-26	2.24E-23
translational initiation (GO:0006413)	143	63	9.11	+	6.92	4.13E-28	1.28E-24
cytoplasmic translation (GO:0002181)	45	18	2.87	+	6.28	1.71E-08	3.41E-06

nuclear-transcribed mRNA catabolic process (GO:0000956)	199	62	12.67	+	4.89	3.03E-21	2.61E-18
establishment of protein localization to membrane (GO:0090150)	217	67	13.82	+	4.85	1.11E-22	1.02E-19
mRNA catabolic process (GO:0006402)	214	63	13.63	+	4.62	1.63E-20	1.33E-17
protein targeting (GO:0006605)	278	79	17.70	+	4.46	1.33E-24	1.37E-21
RNA catabolic process (GO:0006401)	243	64	15.47	+	4.14	9.60E-19	7.10E-16
translation (GO:0006412)	383	99	24.39	+	4.06	7.41E-28	1.64E-24
ribosome assembly (GO:0042255)	66	17	4.20	+	4.04	6.94E-06	1.05E-03
peptide biosynthetic process (GO:0043043)	410	103	26.11	+	3.94	4.20E-28	1.09E-24
rRNA processing (GO:0006364)	261	65	16.62	+	3.91	5.67E-18	3.67E-15
mitochondrial translational elongation (GO:0070125)	86	21	5.48	+	3.83	1.25E-06	2.01E-04
regulation of muscle hypertrophy (GO:0014743)	46	11	2.93	+	3.76	4.90E-04	4.79E-02
mitochondrial translational termination (GO:0070126)	88	21	5.60	+	3.75	1.72E-06	2.72E-04
translational termination (GO:0006415)	96	22	6.11	+	3.60	1.75E-06	2.74E-04
ribosomal large subunit biogenesis (GO:0042273)	70	16	4.46	+	3.59	4.50E-05	5.97E-03
rRNA metabolic process (GO:0016072)	288	65	18.34	+	3.54	3.62E-16	1.81E-13
regulation of muscle adaptation (GO:0043502)	71	16	4.52	+	3.54	5.21E-05	6.80E-03
amide biosynthetic process (GO:0043604)	480	107	30.57	+	3.50	1.56E-25	1.86E-22
nucleobase-containing compound catabolic process (GO:0034655)	362	78	23.05	+	3.38	3.76E-18	2.65E-15
translational elongation (GO:0006414)	121	26	7.71	+	3.37	6.03E-07	1.05E-04
ribosome biogenesis (GO:0042254)	334	71	21.27	+	3.34	2.29E-16	1.18E-13

establishment of protein localization to organelle (GO:0072594)	372	79	23.69	+	3.33	4.71E-18	3.18E-15
peptide metabolic process (GO:0006518)	537	112	34.20	+	3.28	1.26E-24	1.40E-21
ribosomal small subunit biogenesis (GO:0042274)	73	15	4.65	+	3.23	2.14E-04	2.48E-02
heterocycle catabolic process (GO:0046700)	407	81	25.92	+	3.13	4.72E-17	2.82E-14
protein localization to membrane (GO:0072657)	407	81	25.92	+	3.13	4.72E-17	2.71E-14
cellular nitrogen compound catabolic process (GO:0044270)	409	81	26.05	+	3.11	6.03E-17	3.34E-14
mitochondrial translation (GO:0032543)	108	21	6.88	+	3.05	2.64E-05	3.62E-03
aromatic compound catabolic process (GO:0019439)	422	82	26.87	+	3.05	1.01E-16	5.38E-14
protein complex disassembly (GO:0043241)	189	36	12.04	+	2.99	6.54E-08	1.21E-05
cellular protein complex disassembly (GO:0043624)	131	24	8.34	+	2.88	1.72E-05	2.47E-03
organic cyclic compound catabolic process (GO:1901361)	454	83	28.91	+	2.87	1.40E-15	6.79E-13
ncRNA processing (GO:0034470)	405	72	25.79	+	2.79	4.87E-13	1.84E-10
mitochondrial gene expression (GO:0140053)	124	22	7.90	+	2.79	5.80E-05	7.50E-03
ribonucleoprotein complex biogenesis (GO:0022613)	468	83	29.80	+	2.78	7.36E-15	3.36E-12
mucopolysaccharide metabolic process (GO:1903510)	109	19	6.94	+	2.74	2.21E-04	2.54E-02
cellular amide metabolic process (GO:0043603)	695	120	44.26	+	2.71	2.91E-20	2.26E-17
macromolecular complex disassembly (GO:0032984)	221	38	14.07	+	2.70	3.91E-07	6.98E-05
protein localization to organelle (GO:0033365)	611	99	38.91	+	2.54	3.26E-15	1.53E-12
intracellular protein transport (GO:0006886)	744	118	47.38	+	2.49	1.92E-17	1.19E-14
ncRNA metabolic process (GO:0034660)	566	82	36.04	+	2.27	1.24E-10	3.86E-08

viral process (GO:0016032)	662	93	42.16	+	2.21	3.95E-11	1.28E-08
ribonucleoprotein complex assembly (GO:0022618)	207	29	13.18	+	2.20	2.49E-04	2.74E-02
organonitrogen compound biosynthetic process (GO:1901566)	1438	201	91.57	+	2.19	2.83E-23	2.74E-20
regulation of muscle system process (GO:0090257)	215	30	13.69	+	2.19	1.94E-04	2.29E-02
ribonucleoprotein complex subunit organization (GO:0071826)	220	30	14.01	+	2.14	3.79E-04	3.82E-02
cellular component disassembly (GO:0022411)	419	57	26.68	+	2.14	6.65E-07	1.13E-04
symbiosis, encompassing mutualism through parasitism (GO:0044403)	734	97	46.74	+	2.08	2.20E-10	6.22E-08
interspecies interaction between organisms (GO:0044419)	771	100	49.10	+	2.04	3.29E-10	8.66E-08
mRNA metabolic process (GO:0016071)	680	86	43.30	+	1.99	1.63E-08	3.29E-06
cellular protein localization (GO:0034613)	1332	164	84.82	+	1.93	1.94E-14	8.37E-12
cellular macromolecule localization (GO:0070727)	1342	165	85.46	+	1.93	1.62E-14	7.20E-12
cellular macromolecule catabolic process (GO:0044265)	854	102	54.38	+	1.88	1.17E-08	2.56E-06
blood vessel morphogenesis (GO:0048514)	386	45	24.58	+	1.83	2.90E-04	3.13E-02
macromolecule catabolic process (GO:0009057)	997	116	63.49	+	1.83	3.96E-09	9.31E-07
cardiovascular system development (GO:0072358)	500	57	31.84	+	1.79	8.59E-05	1.07E-02
circulatory system development (GO:0072359)	808	91	51.46	+	1.77	8.60E-07	1.42E-04
protein transport (GO:0015031)	1362	153	86.74	+	1.76	1.11E-10	3.52E-08
intracellular transport (GO:0046907)	1265	142	80.56	+	1.76	5.52E-10	1.41E-07
blood vessel development (GO:0001568)	466	52	29.68	+	1.75	3.27E-04	3.38E-02
peptide transport (GO:0015833)	1386	154	88.26	+	1.74	2.23E-10	6.19E-08

amide transport (GO:0042886)	1408	156	89.66	+	1.74	1.55E-10	4.45E-08
establishment of protein localization (GO:0045184)	1439	159	91.64	+	1.74	1.54E-10	4.51E-08
RNA processing (GO:0006396)	897	99	57.12	+	1.73	7.03E-07	1.19E-04
vasculature development (GO:0001944)	490	54	31.20	+	1.73	3.19E-04	3.32E-02
negative regulation of gene expression (GO:0010629)	1637	174	104.25	+	1.67	3.03E-10	8.12E-08
establishment of localization in cell (GO:0051649)	1494	158	95.14	+	1.66	3.32E-09	7.93E-07
neuron projection development (GO:0031175)	627	66	39.93	+	1.65	2.28E-04	2.56E-02
cellular catabolic process (GO:0044248)	1671	173	106.41	+	1.63	2.20E-09	5.33E-07
protein localization (GO:0008104)	1945	200	123.86	+	1.61	1.49E-10	4.44E-08
nitrogen compound transport (GO:0071705)	1672	171	106.48	+	1.61	5.66E-09	1.31E-06
enzyme linked receptor protein signaling pathway (GO:0007167)	715	73	45.53	+	1.60	2.23E-04	2.55E-02
organic substance catabolic process (GO:1901575)	1655	168	105.39	+	1.59	1.22E-08	2.63E-06
catabolic process (GO:0009056)	1950	195	124.18	+	1.57	1.83E-09	4.51E-07
cellular localization (GO:0051641)	2122	206	135.13	+	1.52	6.75E-09	1.54E-06
negative regulation of signal transduction (GO:0009968)	1176	113	74.89	+	1.51	4.70E-05	6.19E-03
organic substance transport (GO:0071702)	2043	196	130.10	+	1.51	3.45E-08	6.53E-06
macromolecule localization (GO:0033036)	2258	216	143.79	+	1.50	7.59E-09	1.71E-06
negative regulation of response to stimulus (GO:0048585)	1477	141	94.06	+	1.50	5.76E-06	8.86E-04
anatomical structure morphogenesis (GO:0009653)	2021	192	128.70	+	1.49	1.10E-07	2.01E-05
negative regulation of molecular function (GO:0044092)	1178	110	75.02	+	1.47	1.66E-04	1.99E-02

negative regulation of macromolecule metabolic process (GO:0010605)	2546	236	162.13	+	1.46	1.54E-08	3.14E-06
negative regulation of metabolic process (GO:0009892)	2795	256	177.99	+	1.44	8.08E-09	1.79E-06
negative regulation of cell communication (GO:0010648)	1273	116	81.07	+	1.43	2.80E-04	3.07E-02
negative regulation of signaling (GO:0023057)	1277	116	81.32	+	1.43	2.87E-04	3.12E-02
macromolecular complex subunit organization (GO:0043933)	1768	160	112.59	+	1.42	1.87E-05	2.64E-03
small molecule metabolic process (GO:0044281)	1815	160	115.58	+	1.38	6.81E-05	8.74E-03
multi-organism process (GO:0051704)	2348	206	149.53	+	1.38	5.91E-06	9.00E-04
regulation of intracellular signal transduction (GO:1902531)	1800	156	114.63	+	1.36	1.79E-04	2.12E-02
regulation of catalytic activity (GO:0050790)	2317	200	147.55	+	1.36	2.19E-05	3.07E-03
regulation of signal transduction (GO:0009966)	3083	266	196.33	+	1.35	6.09E-07	1.05E-04
regulation of phosphorus metabolic process (GO:0051174)	1737	149	110.62	+	1.35	4.52E-04	4.44E-02
cellular component organization or biogenesis (GO:0071840)	5505	472	350.57	+	1.35	1.65E-12	6.10E-10
cellular component biogenesis (GO:0044085)	2610	223	166.21	+	1.34	1.23E-05	1.82E-03
organonitrogen compound metabolic process (GO:1901564)	5534	472	352.42	+	1.34	3.56E-12	1.23E-09
negative regulation of biological process (GO:0048519)	4952	422	315.35	+	1.34	1.39E-10	4.22E-08
cellular nitrogen compound biosynthetic process (GO:0044271)	3489	293	222.19	+	1.32	1.19E-06	1.92E-04
cellular protein metabolic process (GO:0044267)	3752	315	238.94	+	1.32	4.34E-07	7.66E-05
cellular biosynthetic process (GO:0044249)	4689	391	298.61	+	1.31	1.48E-08	3.06E-06
organic substance biosynthetic process (GO:1901576)	4784	398	304.66	+	1.31	1.26E-08	2.64E-06
protein metabolic process (GO:0019538)	4466	371	284.40	+	1.30	5.98E-08	1.12E-05

biosynthetic process (GO:0009058)	4853	401	309.05	+	1.30	2.19E-08	4.31E-06
regulation of cell communication (GO:0010646)	3374	277	214.86	+	1.29	1.59E-05	2.32E-03
regulation of signaling (GO:0023051)	3423	280	217.98	+	1.28	1.78E-05	2.53E-03
regulation of localization (GO:0032879)	2595	212	165.26	+	1.28	2.93E-04	3.14E-02
response to organic substance (GO:0010033)	2765	225	176.08	+	1.28	2.23E-04	2.53E-02
regulation of response to stimulus (GO:0048583)	4078	330	259.70	+	1.27	5.57E-06	8.64E-04
macromolecule biosynthetic process (GO:0009059)	3763	304	239.64	+	1.27	1.59E-05	2.31E-03
regulation of multicellular organismal process (GO:0051239)	2801	226	178.37	+	1.27	3.33E-04	3.42E-02
regulation of molecular function (GO:0065009)	3391	273	215.95	+	1.26	7.31E-05	9.23E-03
cellular component organization (GO:0016043)	5280	424	336.24	+	1.26	1.85E-07	3.34E-05
cellular macromolecule biosynthetic process (GO:0034645)	3698	296	235.50	+	1.26	4.34E-05	5.81E-03
gene expression (GO:0010467)	3783	299	240.91	+	1.24	1.08E-04	1.31E-02
negative regulation of cellular process (GO:0048523)	4457	346	283.83	+	1.22	8.98E-05	1.11E-02
cellular nitrogen compound metabolic process (GO:0034641)	5192	401	330.64	+	1.21	2.30E-05	3.19E-03
transport (GO:0006810)	4326	332	275.49	+	1.21	3.03E-04	3.23E-02
system development (GO:0048731)	4200	321	267.47	+	1.20	5.10E-04	4.95E-02
establishment of localization (GO:0051234)	4442	338	282.88	+	1.19	4.52E-04	4.47E-02
localization (GO:0051179)	5433	413	345.99	+	1.19	6.90E-05	8.79E-03
anatomical structure development (GO:0048856)	5151	390	328.03	+	1.19	1.96E-04	2.28E-02
multicellular organism development (GO:0007275)	4795	362	305.36	+	1.19	4.41E-04	4.39E-02

metabolic process (GO:0008152)	9970	752	634.91	+	1.18	6.09E-10	1.52E-07
cellular metabolic process (GO:0044237)	9070	682	577.60	+	1.18	2.89E-08	5.54E-06
developmental process (GO:0032502)	5511	412	350.95	+	1.17	3.05E-04	3.22E-02
organic substance metabolic process (GO:0071704)	9521	711	606.32	+	1.17	2.86E-08	5.54E-06
regulation of metabolic process (GO:0019222)	6672	498	424.89	+	1.17	3.99E-05	5.38E-03
nitrogen compound metabolic process (GO:0006807)	8641	643	550.28	+	1.17	7.34E-07	1.23E-04
primary metabolic process (GO:0044238)	9163	676	583.52	+	1.16	8.88E-07	1.45E-04
macromolecule metabolic process (GO:0043170)	7782	566	495.57	+	1.14	1.34E-04	1.61E-02
cellular process (GO:0009987)	15086	1055	960.71	+	1.10	1.22E-08	2.60E-06
regulation of biological process (GO:0050789)	11425	797	727.57	+	1.10	2.32E-04	2.60E-02
biological regulation (GO:0065007)	12072	842	768.77	+	1.10	8.24E-05	1.03E-02
biological_process (GO:0008150)	17500	1207	1114.44	+	1.08	2.77E-12	1.00E-09
G-protein coupled receptor signaling pathway (GO:0007186)	1306	52	83.17	-	.63	3.08E-04	3.24E-02
nervous system process (GO:0050877)	1281	51	81.58	-	.63	3.44E-04	3.52E-02
Unclassified (UNCLASSIFIED)	3542	133	225.56	-	.59	2.77E-12	9.77E-10
adaptive immune response (GO:0002250)	434	10	27.64	-	.36	2.48E-04	2.75E-02
sensory perception (GO:0007600)	946	18	60.24	-	.30	4.02E-10	1.04E-07
detection of stimulus (GO:0051606)	675	8	42.99	-	.19	2.26E-10	6.15E-08
adaptive immune response based on somatic recombination of immune receptors built from immunoglobulin superfamily domains (GO:0002460)	238	2	15.16	-	.13	9.16E-05	1.12E-02
immunoglobulin mediated immune response (GO:0016064)	179	1	11.40	-	.09	3.74E-04	3.80E-02

B cell mediated immunity (GO:0019724)	180	1	11.46	-	.09	3.81E-04	3.82E-02
keratinization (GO:0031424)	227	1	14.46	-	.07	2.79E-05	3.81E-03
sensory perception of smell (GO:0007608)	458	1	29.17	-	.03	2.28E-11	7.53E-09
detection of stimulus involved in sensory perception (GO:0050906)	524	1	33.37	-	.03	4.53E-13	1.76E-10
sensory perception of chemical stimulus (GO:0007606)	533	1	33.94	-	.03	1.92E-13	7.83E-11
detection of chemical stimulus involved in sensory perception of smell (GO:0050911)	429	0	27.32	-	< 0.01	3.91E-12	1.32E-09
detection of chemical stimulus involved in sensory perception (GO:0050907)	472	0	30.06	-	< 0.01	3.78E-13	1.51E-10
detection of chemical stimulus (GO:0009593)	507	0	32.29	-	< 0.01	3.09E-14	1.30E-11

Supplementary table 3. Sleuth output for genes differentially expressed between CTNNB1-wildtype and CTNNB1-mutated tumors

	target_id	pval	qval	b	se_b	mean_obs	var_obs	tech_var	sigma_sq	smooth_sigma_sq	final_sigma_sq	Expression CTNNB1 mutated	Expression CTNNB1 wildtype
1	RSPO3	9.08E-31	1.59E-26	2.99	0.26	6.10	1.68	0.01	0.15	0.04	0.15	46.63	847.85
2	OMD	5.70E-29	4.99E-25	2.16	0.19	6.42	0.89	0.00	0.09	0.03	0.09	110.16	980.14
3	KLHDC8A	1.89E-22	1.10E-18	2.61	0.27	5.54	1.33	0.01	0.17	0.05	0.17	32.81	462.55
4	PRELP	4.75E-18	2.08E-14	1.32	0.15	7.07	0.35	0.00	0.06	0.03	0.06	435.64	1560.30
5	FZD1	1.84E-17	6.44E-14	1.32	0.16	7.22	0.35	0.00	0.06	0.03	0.06	492.94	1829.56
6	PTGER3	2.95E-17	8.61E-14	-4.36	0.52	3.64	3.86	0.05	0.59	0.17	0.59	1512.10	23.28
7	LIX1	5.09E-17	1.27E-13	-4.01	0.48	1.63	3.21	0.23	0.25	0.32	0.32	196.71	2.07
8	NR2F1	1.05E-16	2.30E-13	1.46	0.18	5.89	0.43	0.00	0.07	0.04	0.07	114.55	498.37
9	SLC7A8	1.56E-16	3.04E-13	-1.46	0.18	6.64	0.44	0.00	0.07	0.03	0.07	2505.05	588.91
10	MME	2.66E-16	4.66E-13	-3.35	0.41	3.04	2.30	0.11	0.30	0.21	0.30	450.96	11.46
11	FAM160A1	7.41E-16	1.18E-12	2.13	0.26	3.98	0.89	0.02	0.10	0.14	0.14	10.00	85.51
12	ISYNA1	1.25E-15	1.56E-12	1.76	0.22	6.77	0.64	0.00	0.11	0.03	0.11	220.63	1305.54
13	OGN	1.08E-15	1.56E-12	2.40	0.30	6.87	1.19	0.00	0.21	0.03	0.21	141.63	1758.91
14	STON2	1.16E-15	1.56E-12	-1.96	0.24	5.87	0.79	0.01	0.13	0.04	0.13	1985.83	246.83
15	PLAC9	1.54E-15	1.80E-12	1.84	0.23	5.28	0.70	0.01	0.12	0.06	0.12	51.28	293.23
16	SYTL5	6.70E-14	7.34E-11	3.48	0.47	7.81	2.56	0.00	0.52	0.03	0.52	337.10	5393.61
17	CHN1	2.39E-13	2.46E-10	-1.58	0.22	5.70	0.53	0.01	0.10	0.05	0.10	1070.88	231.89
18	FBLN1	1.03E-12	9.74E-10	1.62	0.23	6.62	0.57	0.00	0.12	0.03	0.12	222.29	1082.04
19	PACSIN3	1.06E-12	9.74E-10	1.17	0.16	6.73	0.30	0.00	0.06	0.03	0.06	365.31	1065.44
20	TNFSF4	1.54E-12	1.35E-09	-3.32	0.47	5.73	2.38	0.01	0.52	0.04	0.52	5255.70	210.19
21	SHC2	2.48E-12	2.07E-09	1.44	0.21	5.81	0.45	0.00	0.10	0.04	0.10	112.37	457.59
22	CCDC74A	2.68E-12	2.13E-09	1.73	0.25	6.32	0.65	0.00	0.14	0.03	0.14	171.66	813.05
23	KLHL14	4.94E-12	3.77E-09	-3.15	0.46	1.97	2.09	0.21	0.20	0.29	0.29	136.32	3.80
24	EGFLAM	5.45E-12	3.98E-09	1.90	0.28	5.12	0.79	0.01	0.17	0.07	0.17	40.78	261.62
25	NKD1	7.86E-12	5.51E-09	-2.82	0.41	3.47	1.74	0.03	0.37	0.18	0.37	493.52	19.34

26	SV2C	9.36E-12	6.31E-09	-2.98	0.44	3.12	1.95	0.09	0.37	0.20	0.37	295.83	15.54
27	MGP	1.03E-11	6.66E-09	1.32	0.19	10.45	0.39	0.00	0.09	0.06	0.09	13567.61	46151.93
28	CYTH1	1.80E-11	1.13E-08	-0.88	0.13	6.09	0.16	0.00	0.03	0.04	0.04	899.86	375.55
29	ISM1	3.45E-11	2.08E-08	-4.97	0.75	4.01	5.49	0.09	1.26	0.14	1.26	6616.19	36.13
30	RASGRF1	9.19E-11	5.37E-08	-3.60	0.56	2.80	2.91	0.36	0.38	0.22	0.38	314.09	11.79
31	TLE1	1.13E-10	6.39E-08	-0.80	0.12	6.69	0.14	0.00	0.04	0.03	0.04	1533.80	700.84
32	ZNF365	1.27E-10	6.98E-08	-1.90	0.29	3.48	0.76	0.03	0.12	0.18	0.18	177.28	22.71
33	SULT1B1	1.37E-10	7.30E-08	-2.03	0.32	3.85	0.93	0.00	0.24	0.15	0.24	318.70	32.61
34	LRP4	1.87E-10	9.63E-08	1.19	0.19	4.91	0.29	0.00	0.05	0.08	0.08	54.09	173.32
35	LNPEP	2.17E-10	1.09E-07	-0.83	0.13	7.15	0.16	0.00	0.04	0.03	0.04	2579.96	1085.52
36	C12orf57	2.40E-10	1.17E-07	0.74	0.12	7.70	0.12	0.00	0.03	0.03	0.03	1233.24	2585.83
37	PDE11A	2.72E-10	1.29E-07	-2.61	0.41	3.24	1.55	0.05	0.36	0.19	0.36	352.13	15.68
38	HYI	4.03E-10	1.86E-07	0.97	0.16	6.47	0.22	0.00	0.06	0.03	0.06	315.78	790.84
39	CAB39L	4.45E-10	2.00E-07	0.97	0.16	7.74	0.21	0.00	0.06	0.03	0.06	1091.69	2864.47
40	VAV3	5.63E-10	2.47E-07	-1.42	0.23	4.82	0.46	0.02	0.11	0.09	0.11	403.59	98.94
41	CDS1	5.92E-10	2.53E-07	-0.93	0.15	6.82	0.20	0.00	0.05	0.03	0.05	2087.04	770.87
42	TMEM26	7.33E-10	3.06E-07	-1.68	0.27	4.29	0.62	0.05	0.09	0.12	0.12	323.47	54.47
43	SEC24A	8.86E-10	3.61E-07	-0.71	0.12	7.48	0.10	0.00	0.02	0.03	0.03	3145.20	1544.65
44	FMOD	1.04E-09	4.16E-07	1.36	0.22	5.22	0.43	0.00	0.12	0.07	0.12	61.91	256.52
45	SPINT2	1.07E-09	4.16E-07	1.32	0.22	5.84	0.40	0.00	0.11	0.04	0.11	128.23	463.23
46	LMNA	1.47E-09	5.60E-07	0.72	0.12	8.01	0.12	0.00	0.03	0.03	0.03	1699.24	3543.53
47	HSD3BP2	2.00E-09	7.46E-07	0.97	0.16	7.50	0.22	0.00	0.06	0.03	0.06	865.59	2252.88
48	C1orf198	3.20E-09	1.15E-06	0.87	0.15	5.74	0.18	0.00	0.05	0.04	0.05	161.61	376.30
49	IGFBP6	3.18E-09	1.15E-06	1.80	0.30	7.34	0.76	0.00	0.22	0.03	0.22	369.42	2449.46
50	SOWAHA	3.42E-09	1.20E-06	-3.41	0.58	2.40	2.74	0.21	0.59	0.25	0.59	309.79	6.45
51	AJAP1	3.51E-09	1.21E-06	-3.25	0.55	2.64	2.49	0.06	0.66	0.24	0.66	320.01	8.74
52	CPN2	4.61E-09	1.55E-06	-2.58	0.44	4.60	1.57	0.02	0.45	0.10	0.45	1156.01	67.21
53	MEGF9	5.04E-09	1.67E-06	-0.69	0.12	7.95	0.10	0.00	0.02	0.03	0.03	4944.98	2505.12
54	TTC39A	5.15E-09	1.67E-06	1.35	0.23	5.42	0.43	0.01	0.12	0.06	0.12	77.03	311.98

55	GRIK4	6.16E-09	1.96E-06	-1.76	0.30	3.30	0.68	0.03	0.14	0.19	0.19	113.16	20.18
56	TUBB2A	7.20E-09	2.25E-06	-1.48	0.26	5.12	0.52	0.01	0.15	0.07	0.15	618.56	131.07
57	CHST1	7.98E-09	2.45E-06	-2.81	0.49	3.05	1.88	0.21	0.36	0.20	0.36	456.69	12.67
58	ADH1B	1.03E-08	3.10E-06	0.94	0.16	9.53	0.21	0.00	0.06	0.04	0.06	6666.91	17139.04
59	AFAP1L1	1.13E-08	3.35E-06	-1.58	0.28	5.57	0.60	0.01	0.18	0.05	0.18	1309.77	194.08
60	TSPAN14	1.15E-08	3.37E-06	-0.74	0.13	6.53	0.13	0.00	0.04	0.03	0.04	1262.49	597.72
61	COL12A1	1.22E-08	3.51E-06	1.52	0.27	7.98	0.55	0.00	0.17	0.03	0.17	892.81	4318.73
62	SESTD1	1.57E-08	4.38E-06	-0.83	0.15	5.68	0.16	0.01	0.04	0.05	0.05	601.61	250.43
63	SSPN	1.57E-08	4.38E-06	0.86	0.15	5.52	0.16	0.00	0.03	0.05	0.05	126.24	301.06
64	OSBPL6	1.72E-08	4.71E-06	-2.06	0.37	4.21	1.03	0.11	0.22	0.13	0.22	520.87	47.10
65	PDK4	2.11E-08	5.69E-06	-1.35	0.24	8.22	0.44	0.00	0.14	0.03	0.14	11525.46	3030.72
66	HSD17B8	2.22E-08	5.90E-06	0.72	0.13	6.48	0.13	0.00	0.04	0.03	0.04	376.20	766.34
67	SRPX2	2.75E-08	7.20E-06	1.06	0.19	5.64	0.27	0.00	0.08	0.05	0.08	122.73	362.46
68	ATP10D	2.86E-08	7.38E-06	-1.28	0.23	6.07	0.40	0.00	0.12	0.04	0.12	1380.46	347.64
69	PLA2G16	3.15E-08	7.99E-06	1.24	0.22	6.87	0.38	0.00	0.12	0.03	0.12	409.24	1270.87
70	WNT2B	3.53E-08	8.85E-06	1.19	0.22	5.12	0.35	0.02	0.10	0.07	0.10	64.40	224.99
71	ATXN2	3.75E-08	9.26E-06	-0.67	0.12	7.39	0.11	0.00	0.03	0.03	0.03	2901.74	1427.62
72	PIFO	4.20E-08	1.02E-05	1.39	0.25	5.68	0.47	0.01	0.14	0.05	0.14	98.96	414.20
73	AMHR2	4.61E-08	1.11E-05	1.16	0.21	7.81	0.33	0.00	0.11	0.03	0.11	1009.13	3251.59
74	GSTM5	5.35E-08	1.27E-05	1.41	0.26	4.02	0.48	0.02	0.13	0.14	0.14	18.60	78.48
75	ARL2	6.44E-08	1.50E-05	0.88	0.16	7.27	0.19	0.00	0.06	0.03	0.06	736.76	1749.98
76	PCSK1	7.25E-08	1.67E-05	-3.21	0.60	3.48	2.56	0.08	0.78	0.18	0.78	1030.17	20.37
77	CXXC5	8.26E-08	1.88E-05	0.91	0.17	5.26	0.21	0.00	0.07	0.06	0.07	98.80	236.00
78	GRTP1	8.47E-08	1.90E-05	0.98	0.18	5.68	0.24	0.01	0.07	0.05	0.07	138.66	365.97
79	GALC	8.93E-08	1.98E-05	-0.63	0.12	6.82	0.10	0.00	0.03	0.03	0.03	1535.44	822.28
80	PRRX1	9.27E-08	2.03E-05	1.54	0.29	5.67	0.60	0.00	0.20	0.05	0.20	92.26	429.54
81	CD9	9.86E-08	2.13E-05	1.21	0.23	8.84	0.36	0.00	0.12	0.04	0.12	2951.04	9142.52
82	PXMP4	1.02E-07	2.18E-05	0.70	0.13	5.98	0.11	0.00	0.03	0.04	0.04	236.73	454.26
83	EIF4EBP3	1.05E-07	2.21E-05	1.09	0.21	6.30	0.30	0.00	0.10	0.03	0.10	251.91	691.12

84	KLF5	1.11E-07	2.32E-05	1.87	0.35	4.67	0.87	0.05	0.25	0.10	0.25	27.70	171.70
85	RALBP1	1.21E-07	2.49E-05	-1.14	0.22	6.63	0.33	0.00	0.11	0.03	0.11	2175.84	611.37
86	TMEM132E	1.25E-07	2.55E-05	-4.62	0.87	3.00	5.36	0.12	1.72	0.21	1.72	4209.11	10.81
87	SYNPO2	1.30E-07	2.62E-05	1.16	0.22	7.52	0.34	0.00	0.11	0.03	0.11	772.65	2434.79
88	GNAI3	1.58E-07	3.15E-05	-0.61	0.12	7.60	0.09	0.00	0.03	0.03	0.03	3324.49	1774.98
89	FOXF2	1.87E-07	3.63E-05	-3.59	0.69	2.41	3.26	0.22	0.92	0.25	0.92	417.01	7.18
90	GMPR	1.89E-07	3.63E-05	0.70	0.13	5.87	0.12	0.00	0.04	0.04	0.04	211.56	409.79
91	SORBS1	1.86E-07	3.63E-05	0.72	0.14	6.69	0.13	0.00	0.04	0.03	0.04	459.18	947.10
92	SPP1	1.93E-07	3.67E-05	-2.41	0.46	4.58	1.47	0.02	0.50	0.10	0.50	838.24	76.72
93	SH3D19	2.04E-07	3.81E-05	0.71	0.14	7.47	0.13	0.00	0.04	0.03	0.04	1006.69	2071.64
94	THBS4	2.05E-07	3.81E-05	-1.99	0.38	3.51	1.00	0.03	0.32	0.17	0.32	259.08	22.67
95	NDUFS8	2.26E-07	4.12E-05	0.62	0.12	8.04	0.09	0.00	0.03	0.03	0.03	1914.57	3562.74
96	PAX6	2.24E-07	4.12E-05	-2.29	0.44	3.37	1.33	0.02	0.45	0.18	0.45	225.21	22.72
97	CBLN4	2.40E-07	4.33E-05	2.34	0.45	6.66	1.40	0.00	0.49	0.03	0.49	232.42	1368.82
98	TIGAR	2.42E-07	4.33E-05	-0.72	0.14	6.48	0.13	0.01	0.04	0.03	0.04	1196.73	574.44
99	MLST8	2.83E-07	5.01E-05	0.65	0.13	6.15	0.10	0.00	0.03	0.04	0.04	281.95	539.24
100	EGFR	2.88E-07	5.05E-05	-1.16	0.23	6.81	0.35	0.00	0.12	0.03	0.12	2426.67	758.59
101	KCNT2	3.03E-07	5.26E-05	1.12	0.22	4.74	0.32	0.02	0.10	0.09	0.10	51.16	148.96
102	PKIG	3.14E-07	5.40E-05	0.65	0.13	7.71	0.11	0.00	0.04	0.03	0.04	1378.05	2574.34
103	CCDC28B	3.40E-07	5.58E-05	0.85	0.17	5.91	0.19	0.00	0.06	0.04	0.06	197.89	447.38
104	CRYAB	3.38E-07	5.58E-05	0.71	0.14	6.28	0.13	0.00	0.04	0.03	0.04	301.34	626.62
105	IGLON5	3.36E-07	5.58E-05	-2.73	0.53	3.44	1.91	0.06	0.62	0.18	0.62	409.36	24.17
106	MAPK4	3.41E-07	5.58E-05	-1.81	0.36	6.63	0.85	0.00	0.30	0.03	0.30	3557.92	591.34
107	TSC22D1	3.34E-07	5.58E-05	-1.00	0.20	7.31	0.26	0.00	0.09	0.03	0.09	3510.58	1280.18
108	BOC	3.58E-07	5.81E-05	1.20	0.24	4.33	0.32	0.01	0.06	0.12	0.12	30.20	98.29
109	RPS28P7	3.77E-07	6.07E-05	0.62	0.12	6.77	0.10	0.00	0.03	0.03	0.03	543.16	999.73
110	ALG13	3.85E-07	6.08E-05	-0.71	0.14	6.60	0.12	0.01	0.03	0.03	0.03	1291.89	648.68
111	PHC2	3.85E-07	6.08E-05	-0.62	0.12	7.13	0.09	0.00	0.02	0.03	0.03	2073.19	1110.07
112	B9D1	4.00E-07	6.25E-05	0.70	0.14	5.82	0.11	0.00	0.03	0.04	0.04	195.71	392.52

113	THEM6	4.34E-07	6.73E-05	0.77	0.15	6.77	0.15	0.00	0.05	0.03	0.05	490.42	1030.27
114	CYB5RL	4.38E-07	6.73E-05	0.66	0.13	6.28	0.11	0.01	0.04	0.03	0.04	327.28	619.47
115	COL4A2	4.85E-07	7.40E-05	-1.07	0.21	5.92	0.30	0.00	0.11	0.04	0.11	942.23	313.33
116	HSD3B1	5.02E-07	7.59E-05	1.01	0.20	4.74	0.26	0.01	0.09	0.09	0.09	55.56	142.77
117	CHL1	5.22E-07	7.78E-05	-4.30	0.86	4.59	4.80	0.10	1.66	0.10	1.66	4221.41	134.36
118	FEM1C	5.24E-07	7.78E-05	-0.64	0.13	7.28	0.11	0.00	0.04	0.03	0.04	2446.00	1305.28
119	FGR	5.67E-07	8.35E-05	-1.61	0.32	4.64	0.68	0.03	0.22	0.10	0.22	435.77	80.92
120	SPTBN2	7.64E-07	0.000111594	0.65	0.13	6.28	0.10	0.01	0.02	0.03	0.03	320.40	618.83
121	CALD1	7.77E-07	0.000111823	0.63	0.13	7.85	0.11	0.00	0.04	0.03	0.04	1589.28	2958.77
122	STXBP5	7.78E-07	0.000111823	-0.75	0.15	6.63	0.15	0.00	0.05	0.03	0.05	1400.10	665.85
123	C9orf116	8.05E-07	0.000113801	0.85	0.17	5.37	0.19	0.01	0.07	0.06	0.07	110.93	261.24
124	SORCS2	8.02E-07	0.000113801	1.36	0.28	4.87	0.49	0.05	0.14	0.08	0.14	44.50	188.90
125	OTUD4	8.69E-07	0.000120393	-0.68	0.14	5.77	0.11	0.00	0.03	0.04	0.04	572.73	283.18
126	PDE1A	8.72E-07	0.000120393	1.35	0.28	6.15	0.48	0.01	0.18	0.04	0.18	210.06	638.19
127	TNNC1	8.72E-07	0.000120393	2.41	0.49	5.28	1.53	0.01	0.57	0.06	0.57	32.13	400.97
128	PELI3	9.06E-07	0.000124084	0.61	0.12	6.28	0.09	0.00	0.03	0.03	0.03	340.69	607.69
129	PEMT	9.41E-07	0.000127889	0.70	0.14	5.84	0.13	0.00	0.05	0.04	0.05	207.96	399.89
130	RSAD1	1.05E-06	0.000141317	0.58	0.12	6.56	0.07	0.00	0.01	0.03	0.03	452.75	794.56
131	G0S2	1.07E-06	0.000143239	1.26	0.26	6.63	0.42	0.00	0.16	0.03	0.16	297.55	1033.47
132	LRP3	1.09E-06	0.000145341	0.77	0.16	7.43	0.16	0.00	0.06	0.03	0.06	918.51	2021.43
133	PHACTR3	1.25E-06	0.000164334	-1.62	0.33	4.18	0.70	0.02	0.25	0.13	0.25	374.38	48.02
134	ECM2	1.31E-06	0.000168897	1.07	0.22	6.57	0.30	0.01	0.11	0.03	0.11	306.86	939.32
135	EP400	1.30E-06	0.000168897	-0.71	0.15	6.45	0.13	0.00	0.05	0.03	0.05	1129.71	560.36
136	FKBP9P1	1.31E-06	0.000168897	1.21	0.25	4.42	0.39	0.01	0.14	0.12	0.14	31.45	114.04
137	PLEKHA7	1.35E-06	0.000173272	-1.82	0.38	3.45	0.88	0.06	0.28	0.18	0.28	173.42	23.61
138	SEMA3D	1.37E-06	0.000173712	1.86	0.38	6.53	0.92	0.00	0.35	0.03	0.35	185.81	1139.03
139	IMPDH2	1.39E-06	0.000175385	0.57	0.12	7.52	0.09	0.00	0.03	0.03	0.03	1201.61	2094.01
140	SLC26A2	1.53E-06	0.000192105	-1.16	0.24	7.11	0.36	0.00	0.14	0.03	0.14	3471.67	1023.59
141	EBF4	1.59E-06	0.000197342	1.27	0.27	5.14	0.43	0.03	0.13	0.07	0.13	73.61	226.04

142	HERC1	1.74E-06	0.000214182	-0.60	0.13	6.23	0.08	0.00	0.02	0.04	0.04	839.38	454.90
143	RPL10A	1.78E-06	0.000218212	0.68	0.14	9.99	0.11	0.00	0.03	0.05	0.05	12966.10	25378.82
144	ICK	1.80E-06	0.000218948	-0.67	0.14	8.77	0.12	0.00	0.05	0.04	0.05	11427.85	5689.83
145	PRKD3	1.81E-06	0.000219053	-0.97	0.20	7.23	0.25	0.00	0.10	0.03	0.10	3355.15	1160.19
146	SELENOH	1.92E-06	0.00023073	0.59	0.12	7.63	0.09	0.00	0.04	0.03	0.04	1299.72	2355.44
147	DNAJC4	1.96E-06	0.000233532	0.59	0.12	6.37	0.08	0.00	0.02	0.03	0.03	373.09	663.09
148	ADIRF	2.08E-06	0.000246439	1.05	0.22	6.04	0.30	0.01	0.11	0.04	0.11	190.04	542.08
149	FAM13B	2.22E-06	0.000259412	-0.95	0.20	6.14	0.25	0.00	0.10	0.04	0.10	1065.84	395.22
150	TMEM200A	2.22E-06	0.000259412	1.19	0.25	8.99	0.38	0.00	0.15	0.04	0.15	3510.70	10634.09
151	RNASEH2C	2.30E-06	0.000266873	0.65	0.14	7.00	0.11	0.00	0.04	0.03	0.04	676.42	1270.05
152	MTMR9LP	2.34E-06	0.000269863	1.12	0.24	5.51	0.34	0.01	0.13	0.05	0.13	116.35	316.50
153	SPAG7	2.37E-06	0.000271636	0.58	0.12	6.75	0.09	0.00	0.04	0.03	0.04	550.78	969.73
154	CYP1B1	2.40E-06	0.000271659	1.98	0.42	5.77	1.06	0.00	0.42	0.04	0.42	84.71	557.86
155	KDM1B	2.40E-06	0.000271659	-0.68	0.14	6.48	0.13	0.00	0.05	0.03	0.05	1199.08	576.59
156	CDON	2.56E-06	0.000287639	1.12	0.24	4.72	0.34	0.02	0.12	0.09	0.12	46.09	149.30
157	ITGBL1	2.61E-06	0.000291857	1.65	0.35	6.23	0.75	0.00	0.29	0.04	0.29	148.89	807.37
158	LCP1	2.69E-06	0.000298357	-0.66	0.14	6.87	0.12	0.00	0.05	0.03	0.05	1715.35	859.16
159	FABP5	2.81E-06	0.00030981	-2.09	0.45	3.56	1.19	0.09	0.39	0.17	0.39	310.81	25.26
160	NAPB	2.89E-06	0.000316275	-0.59	0.13	7.38	0.09	0.00	0.04	0.03	0.04	2588.93	1451.29
161	KIF3A	2.93E-06	0.000318768	-1.60	0.34	6.40	0.70	0.00	0.28	0.03	0.28	3561.19	438.86
162	SLC2A3P1	2.95E-06	0.000319612	3.90	0.83	4.08	4.05	1.53	0.02	0.14	0.14	21.03	155.58
163	ENAH	3.25E-06	0.000344295	0.54	0.12	7.36	0.07	0.00	0.02	0.03	0.03	1030.22	1776.06
164	EPCAM	3.20E-06	0.000344295	1.33	0.28	5.69	0.48	0.01	0.19	0.05	0.19	113.20	420.89
165	MRPL24	3.28E-06	0.000344295	0.54	0.12	7.32	0.06	0.00	0.01	0.03	0.03	979.25	1680.74
166	TCEA3	3.22E-06	0.000344295	0.97	0.21	7.23	0.26	0.00	0.10	0.03	0.10	727.45	1723.96
167	ZNF189	3.27E-06	0.000344295	-0.76	0.16	7.98	0.16	0.00	0.06	0.03	0.06	6005.76	2537.98
168	BOLA1	3.33E-06	0.0003451	0.64	0.14	5.86	0.09	0.00	0.01	0.04	0.04	215.02	401.15
169	STBD1	3.31E-06	0.0003451	0.84	0.18	5.55	0.20	0.01	0.07	0.05	0.07	136.39	311.77
170	MRM1	3.52E-06	0.000363011	0.68	0.15	5.64	0.10	0.00	0.02	0.05	0.05	163.09	324.69

171	MTX1	3.56E-06	0.000364458	0.54	0.12	7.04	0.08	0.00	0.03	0.03	0.03	748.88	1288.63
172	HHIP	3.67E-06	0.000373618	2.34	0.51	5.57	1.51	0.01	0.60	0.05	0.60	88.33	476.90
173	DCN	3.69E-06	0.000373714	1.63	0.35	9.17	0.73	0.00	0.30	0.04	0.30	2657.57	15667.57
174	SYNE1	3.77E-06	0.000379834	-0.76	0.16	8.66	0.16	0.00	0.06	0.04	0.06	10861.92	5098.91
175	HSD3BP1	3.83E-06	0.000381307	1.43	0.31	5.05	0.56	0.01	0.22	0.07	0.22	52.10	231.24
176	RPL36A	3.83E-06	0.000381307	0.61	0.13	9.17	0.10	0.00	0.04	0.04	0.04	5916.44	11010.03
177	EEF1D	3.91E-06	0.00038738	0.60	0.13	9.19	0.10	0.00	0.04	0.04	0.04	6151.49	11229.45
178	CLIC5	3.94E-06	0.000388143	1.51	0.33	3.87	0.63	0.01	0.25	0.15	0.25	14.65	73.43
179	SLC25A6	4.08E-06	0.000399406	0.60	0.13	8.23	0.10	0.00	0.04	0.03	0.04	2342.15	4294.36
180	RPS5	4.14E-06	0.000403166	0.62	0.13	9.52	0.09	0.00	0.02	0.04	0.04	8481.40	15451.09
181	MRPS26	4.24E-06	0.000410803	0.53	0.12	7.34	0.07	0.00	0.02	0.03	0.03	1011.77	1731.47
182	SHROOM1	4.56E-06	0.000439136	-1.14	0.25	4.60	0.34	0.05	0.08	0.10	0.10	271.72	82.84
183	AEBP1	4.61E-06	0.000441784	1.35	0.29	6.61	0.50	0.00	0.21	0.03	0.21	313.71	1022.70
184	FOXD2	4.83E-06	0.000460454	1.27	0.28	4.00	0.45	0.02	0.16	0.14	0.16	21.65	75.86
185	MAGEH1	4.86E-06	0.000460796	0.69	0.15	7.18	0.13	0.00	0.05	0.03	0.05	776.40	1550.67
186	C1orf35	5.10E-06	0.000480408	0.73	0.16	5.42	0.11	0.01	0.02	0.06	0.06	126.57	262.74
187	CRYL1	5.28E-06	0.000492098	0.59	0.13	7.35	0.10	0.00	0.04	0.03	0.04	1006.98	1777.95
188	RPL8	5.26E-06	0.000492098	0.62	0.14	9.59	0.09	0.00	0.03	0.04	0.04	9241.59	16601.95
189	OLFML1	5.45E-06	0.000504921	1.01	0.22	5.12	0.29	0.01	0.11	0.07	0.11	83.90	210.11
190	CDK20	5.59E-06	0.000515231	1.01	0.22	6.92	0.29	0.00	0.12	0.03	0.12	545.80	1266.55
191	DNPH1	5.62E-06	0.000515231	0.54	0.12	6.96	0.08	0.00	0.03	0.03	0.03	691.90	1187.40
192	HCFC1R1	5.66E-06	0.000516473	0.68	0.15	6.62	0.13	0.00	0.05	0.03	0.05	465.15	869.61
193	STC2	5.79E-06	0.000525659	-1.73	0.38	2.06	0.81	0.06	0.25	0.28	0.28	49.21	5.37
194	SLC41A3	5.84E-06	0.000528007	0.53	0.12	6.77	0.07	0.00	0.03	0.03	0.03	577.76	985.14
195	CCDC121	5.92E-06	0.000529519	0.84	0.19	5.02	0.15	0.01	0.02	0.07	0.07	77.36	181.31
196	GLCE	5.91E-06	0.000529519	-0.75	0.17	7.79	0.16	0.00	0.07	0.03	0.07	4726.07	2122.67
197	CHRFAM7A	6.02E-06	0.000530928	-2.67	0.59	3.95	2.00	0.31	0.52	0.15	0.52	746.90	42.50
198	COLEC10	6.03E-06	0.000530928	-2.75	0.61	2.98	2.12	0.12	0.77	0.21	0.77	312.22	13.95
199	GAREM1	5.99E-06	0.000530928	-0.78	0.17	6.73	0.17	0.00	0.07	0.03	0.07	1637.89	736.93

200	GLT8D2	6.11E-06	0.000535239	0.92	0.20	4.92	0.24	0.01	0.09	0.08	0.09	68.82	170.56
201	CFH	6.15E-06	0.000536175	0.95	0.21	7.75	0.25	0.00	0.11	0.03	0.11	1128.25	2938.04
202	PQLC2	6.34E-06	0.000550397	0.55	0.12	6.38	0.08	0.00	0.03	0.03	0.03	381.49	668.66
203	NME4	6.67E-06	0.000575988	0.59	0.13	7.40	0.10	0.00	0.04	0.03	0.04	1043.63	1869.22
204	ISLR	6.95E-06	0.000588516	1.86	0.41	5.57	0.97	0.00	0.41	0.05	0.41	95.03	412.37
205	KCNG3	6.91E-06	0.000588516	-0.92	0.20	5.54	0.24	0.00	0.10	0.05	0.10	565.77	218.82
206	PDE3B	6.93E-06	0.000588516	-1.25	0.28	4.81	0.44	0.03	0.16	0.09	0.16	420.76	98.67
207	WFS1	6.91E-06	0.000588516	0.67	0.15	7.19	0.13	0.00	0.05	0.03	0.05	842.17	1539.22
208	TMEM120A	7.17E-06	0.000604186	0.52	0.12	7.30	0.08	0.00	0.03	0.03	0.03	1011.88	1653.87
209	AIP	7.59E-06	0.000636524	0.52	0.12	6.83	0.06	0.00	0.02	0.03	0.03	614.11	1031.98
210	MPI	7.83E-06	0.000653491	0.52	0.12	7.42	0.08	0.00	0.03	0.03	0.03	1099.42	1874.48
211	SLC36A1	7.87E-06	0.000654077	-1.00	0.22	4.52	0.22	0.01	0.04	0.11	0.11	208.05	76.81
212	TCEAL7	8.01E-06	0.000662556	1.17	0.26	4.04	0.36	0.02	0.12	0.14	0.14	22.43	76.38
213	PPIP5K1	8.10E-06	0.00066611	-0.64	0.14	5.92	0.09	0.01	0.02	0.04	0.04	620.88	331.64
214	DVL2	8.23E-06	0.000674345	0.58	0.13	6.03	0.08	0.00	0.02	0.04	0.04	262.93	472.52
215	C20orf27	8.42E-06	0.000684143	0.56	0.13	6.32	0.08	0.00	0.03	0.03	0.03	368.30	628.95
216	CLEC3B	8.43E-06	0.000684143	0.99	0.22	5.97	0.28	0.00	0.12	0.04	0.12	183.51	503.68
217	CNKSR2	8.76E-06	0.000702707	1.48	0.33	5.14	0.62	0.04	0.23	0.07	0.23	64.98	244.81
218	NTHL1	8.78E-06	0.000702707	0.78	0.18	5.15	0.15	0.01	0.05	0.07	0.07	93.58	204.76
219	PPP1R14B	8.77E-06	0.000702707	0.70	0.16	6.94	0.14	0.00	0.06	0.03	0.06	596.52	1228.03
220	FZD2	8.83E-06	0.000703801	1.19	0.27	4.27	0.40	0.02	0.15	0.13	0.15	28.38	97.14
221	RUNX1T1	9.00E-06	0.000713758	1.24	0.28	4.34	0.44	0.02	0.17	0.12	0.17	33.16	103.85
222	EIF1B	9.13E-06	0.000720848	-0.52	0.12	6.64	0.08	0.00	0.03	0.03	0.03	1177.11	699.14
223	DBNDD2	9.35E-06	0.00073363	0.55	0.12	6.72	0.09	0.00	0.03	0.03	0.03	533.59	940.67
224	RPL36	9.38E-06	0.00073363	0.60	0.13	9.55	0.08	0.00	0.02	0.04	0.04	8928.20	15967.44
225	HS3ST2	9.57E-06	0.000742297	-1.48	0.33	4.66	0.62	0.02	0.25	0.10	0.25	370.88	88.43
226	VARS2	9.56E-06	0.000742297	0.53	0.12	6.80	0.07	0.00	0.02	0.03	0.03	589.12	1002.94
227	MARCH6	9.66E-06	0.000745612	-0.51	0.12	7.25	0.07	0.00	0.03	0.03	0.03	2190.41	1280.52
228	MPG	9.71E-06	0.00074647	0.51	0.12	7.02	0.07	0.00	0.03	0.03	0.03	745.96	1261.72

229	SEC14L4	9.84E-06	0.000752744	-2.25	0.51	2.71	1.44	0.19	0.43	0.23	0.43	108.20	12.24
230	TPBG	1.01E-05	0.000769844	-2.16	0.49	4.68	1.33	0.02	0.56	0.10	0.56	1108.46	80.14
231	ACBD4	1.03E-05	0.00077337	0.61	0.14	5.96	0.11	0.00	0.04	0.04	0.04	256.99	442.22
232	DMPK	1.03E-05	0.00077337	0.94	0.21	7.05	0.25	0.00	0.11	0.03	0.11	609.55	1433.85
233	KDELR3	1.02E-05	0.00077337	0.95	0.21	4.60	0.19	0.01	0.03	0.10	0.10	46.72	122.30
234	LRRC75A	1.12E-05	0.000836068	0.55	0.12	8.76	0.07	0.00	0.02	0.04	0.04	4168.35	7121.99
235	RPLP0	1.14E-05	0.000848097	0.62	0.14	9.91	0.09	0.00	0.03	0.05	0.05	12684.10	22864.68
236	ZNF460	1.16E-05	0.000860202	-0.59	0.14	7.67	0.10	0.00	0.04	0.03	0.04	3495.46	1930.91
237	CHADL	1.24E-05	0.000919458	2.15	0.49	4.51	1.33	0.06	0.52	0.11	0.52	16.80	185.05
238	TNFRSF12A	1.26E-05	0.000930001	-0.91	0.21	5.92	0.24	0.00	0.10	0.04	0.10	843.34	320.97
239	JAM3	1.32E-05	0.000971271	0.69	0.16	6.46	0.14	0.00	0.06	0.03	0.06	379.14	745.40
240	DUSP26	1.34E-05	0.00098126	1.33	0.31	6.72	0.51	0.00	0.22	0.03	0.22	322.87	1168.97
241	RPS18	1.37E-05	0.000998088	0.62	0.14	9.92	0.11	0.00	0.05	0.05	0.05	12952.82	23414.06
242	CACNB4	1.39E-05	0.001008463	-1.41	0.32	5.05	0.58	0.02	0.23	0.07	0.23	628.70	124.57
243	CDK5RAP2	1.41E-05	0.001010089	-0.57	0.13	6.05	0.06	0.00	0.01	0.04	0.04	671.39	380.88
244	PKNOX2	1.41E-05	0.001010089	2.96	0.68	3.80	2.53	0.02	1.09	0.16	1.09	4.34	140.90
245	TWSG1	1.41E-05	0.001010089	-1.24	0.28	7.82	0.44	0.00	0.19	0.03	0.19	9169.99	1966.74
246	NLRC5	1.42E-05	0.001014818	-0.74	0.17	5.35	0.14	0.01	0.04	0.06	0.06	385.19	186.08
247	CYP27A1	1.45E-05	0.001025806	0.76	0.17	7.52	0.17	0.00	0.07	0.03	0.07	1084.07	2179.67
248	IZUMO1	1.46E-05	0.001025806	1.03	0.24	4.61	0.24	0.03	0.03	0.10	0.10	46.05	125.53
249	RPL23AP42	1.45E-05	0.001025806	0.55	0.13	8.63	0.09	0.00	0.04	0.04	0.04	3711.92	6358.46
250	PMF1	1.53E-05	0.001065743	0.51	0.12	6.69	0.07	0.00	0.03	0.03	0.03	536.73	900.78
251	PROS1	1.53E-05	0.001065743	0.68	0.16	5.59	0.13	0.00	0.06	0.05	0.06	165.59	311.72
252	AFF3	1.63E-05	0.001130247	-2.42	0.56	4.36	1.70	0.02	0.74	0.12	0.74	904.74	58.63
253	ATP2C1	1.64E-05	0.001130247	-0.82	0.19	8.48	0.20	0.00	0.09	0.04	0.09	9785.72	4207.60
254	CORIN	1.64E-05	0.001130247	-1.25	0.29	4.13	0.46	0.03	0.17	0.14	0.17	203.02	51.14
255	RPL18A	1.64E-05	0.001130247	0.60	0.14	9.79	0.09	0.00	0.03	0.05	0.05	11109.80	20334.52
256	MZT2A	1.66E-05	0.001136484	0.82	0.19	6.70	0.20	0.00	0.08	0.03	0.08	449.23	986.14
257	DNAJB4	1.69E-05	0.001145058	0.58	0.14	7.42	0.10	0.00	0.04	0.03	0.04	1060.72	1917.02

258	UFC1	1.68E-05	0.001145058	0.50	0.12	7.36	0.05	0.00	0.01	0.03	0.03	1062.39	1748.83
259	TMPPE	1.79E-05	0.00121287	-0.71	0.17	5.81	0.15	0.00	0.06	0.04	0.06	595.19	299.16
260	SHISA6	1.81E-05	0.001217521	1.93	0.45	4.40	1.09	0.04	0.45	0.12	0.45	18.07	152.39
261	PLEKHA6	1.88E-05	0.001261005	1.50	0.35	5.50	0.66	0.01	0.28	0.05	0.28	95.90	363.04
262	DMD	1.91E-05	0.001274597	0.73	0.17	6.41	0.16	0.00	0.07	0.03	0.07	374.90	712.44
263	COQ8A	1.94E-05	0.001287002	0.87	0.20	8.89	0.22	0.00	0.10	0.04	0.10	4053.27	8850.04
264	TMEM45B	1.94E-05	0.001287002	-2.44	0.57	2.22	1.75	0.10	0.69	0.27	0.69	252.35	5.45
265	NAXE	2.01E-05	0.001331193	0.49	0.12	7.38	0.06	0.00	0.02	0.03	0.03	1107.60	1775.42
266	JMY	2.14E-05	0.001409706	-0.56	0.13	7.23	0.09	0.00	0.04	0.03	0.04	2256.43	1247.58
267	PNKD	2.16E-05	0.001418017	0.49	0.12	7.47	0.07	0.00	0.03	0.03	0.03	1231.31	1952.65
268	URAHP	2.19E-05	0.001433032	1.37	0.32	3.30	0.46	0.06	0.09	0.19	0.19	9.02	38.09
269	PPP1R35	2.22E-05	0.001444246	0.68	0.16	5.86	0.14	0.01	0.06	0.04	0.06	215.53	406.49
270	METTL26	2.23E-05	0.001444539	0.50	0.12	6.55	0.06	0.00	0.02	0.03	0.03	468.68	783.74
271	PXYLP1	2.25E-05	0.001447604	-1.30	0.31	5.88	0.50	0.00	0.22	0.04	0.22	1217.29	295.21
272	RCN3	2.24E-05	0.001447604	0.93	0.22	4.69	0.25	0.01	0.10	0.10	0.10	52.14	137.27
273	PIGQ	2.28E-05	0.001463971	0.61	0.15	6.47	0.11	0.00	0.05	0.03	0.05	410.91	742.10
274	NCKAP5	2.32E-05	0.001480101	-1.79	0.42	3.51	0.95	0.06	0.37	0.17	0.37	174.68	27.05
275	TMEM176B	2.32E-05	0.001480101	1.49	0.35	6.87	0.66	0.00	0.30	0.03	0.30	368.21	1400.25
276	EVA1C	2.42E-05	0.00152694	-0.77	0.18	5.45	0.18	0.02	0.06	0.05	0.06	457.40	203.30
277	HSPB8	2.43E-05	0.00152694	-2.17	0.51	5.06	1.40	0.00	0.63	0.07	0.63	1182.39	132.96
278	NOL3	2.43E-05	0.00152694	0.62	0.15	6.50	0.11	0.00	0.05	0.03	0.05	422.77	762.78
279	USP38	2.42E-05	0.00152694	-0.50	0.12	8.01	0.07	0.00	0.03	0.03	0.03	4666.78	2730.12
280	CYHR1	2.51E-05	0.001574089	0.51	0.12	7.13	0.08	0.00	0.03	0.03	0.03	855.51	1398.69
281	COL8A1	2.53E-05	0.001576008	0.60	0.14	5.93	0.11	0.00	0.05	0.04	0.05	237.11	430.66
282	NAP1L5	2.55E-05	0.001582833	-0.54	0.13	7.04	0.09	0.00	0.04	0.03	0.04	1791.23	1043.79
283	SLAMF8	2.56E-05	0.00158303	-1.38	0.33	3.72	0.56	0.04	0.22	0.16	0.22	148.15	33.74
284	AKAP12	2.61E-05	0.001610295	0.72	0.17	7.76	0.16	0.00	0.07	0.03	0.07	1336.33	2792.62
285	NR2C2AP	2.66E-05	0.001633452	0.66	0.16	5.44	0.08	0.00	0.00	0.05	0.05	136.93	263.76
286	NIT1	2.73E-05	0.001674319	0.61	0.15	7.52	0.11	0.00	0.05	0.03	0.05	1153.52	2143.77

287	RHOU	2.74E-05	0.001674319	-1.23	0.29	7.90	0.45	0.00	0.21	0.03	0.21	7945.80	2304.43
288	GLB1L3	2.78E-05	0.001674957	-2.04	0.49	4.17	1.24	0.05	0.52	0.13	0.52	670.30	44.35
289	HMBS	2.77E-05	0.001674957	0.61	0.15	5.65	0.09	0.00	0.02	0.05	0.05	177.76	323.98
290	PI4K2B	2.78E-05	0.001674957	-0.57	0.14	6.72	0.10	0.00	0.04	0.03	0.04	1372.48	751.41
291	TMTC4	2.78E-05	0.001674957	0.75	0.18	5.07	0.14	0.00	0.05	0.07	0.07	88.41	189.50
292	CACNA1H	2.86E-05	0.001713998	1.46	0.35	7.94	0.64	0.00	0.29	0.03	0.29	1165.06	4061.08
293	EEF1G	2.89E-05	0.001721191	0.59	0.14	9.91	0.09	0.00	0.03	0.05	0.05	12898.68	22802.96
294	NUDT22	2.89E-05	0.001721191	0.50	0.12	6.62	0.07	0.00	0.03	0.03	0.03	502.71	839.92
295	RPS2	2.92E-05	0.001735883	0.68	0.16	9.75	0.14	0.00	0.06	0.05	0.06	10663.74	20035.86
296	RPS3	2.93E-05	0.001736593	0.57	0.14	9.60	0.08	0.00	0.03	0.04	0.04	9556.37	16818.88
297	GAS2L3	2.99E-05	0.001764452	-1.26	0.30	5.68	0.47	0.01	0.21	0.05	0.21	1144.63	233.47
298	GRIN3A	3.04E-05	0.001780785	-1.29	0.31	3.13	0.43	0.03	0.12	0.20	0.20	76.49	17.81
299	LAMB1	3.03E-05	0.001780785	-1.18	0.28	6.85	0.42	0.00	0.19	0.03	0.19	2721.64	797.99
300	NECTIN3	3.07E-05	0.001791627	-0.73	0.18	5.54	0.16	0.00	0.07	0.05	0.07	503.74	223.32
301	SLC16A4	3.09E-05	0.001797535	0.74	0.18	5.55	0.17	0.01	0.07	0.05	0.07	145.54	306.65
302	OBSL1	3.11E-05	0.001799654	0.57	0.14	8.41	0.10	0.00	0.04	0.04	0.04	2842.98	5128.95
303	TNFAIP3	3.11E-05	0.001799654	-1.57	0.38	6.08	0.74	0.01	0.33	0.04	0.33	2440.37	333.36
304	KLHDC9	3.23E-05	0.001860944	0.63	0.15	5.62	0.10	0.01	0.03	0.05	0.05	167.91	317.47
305	GPR137B	3.27E-05	0.001878665	-0.64	0.15	6.22	0.12	0.00	0.05	0.04	0.05	836.61	453.14
306	GXYLT1	3.29E-05	0.001881889	-0.50	0.12	6.94	0.07	0.00	0.02	0.03	0.03	1530.85	943.92
307	PCBD1	3.31E-05	0.001887835	0.63	0.15	7.42	0.12	0.00	0.06	0.03	0.06	1068.71	1917.02
308	C18orf25	3.41E-05	0.001937979	-0.57	0.14	6.98	0.10	0.00	0.04	0.03	0.04	1815.36	966.40
309	CPEB2	3.49E-05	0.001977192	-0.64	0.16	7.18	0.13	0.01	0.05	0.03	0.05	2229.74	1183.57
310	LEMD1	3.52E-05	0.001987694	1.35	0.33	3.18	0.53	0.06	0.18	0.20	0.20	9.04	33.88
311	TREX1	3.55E-05	0.002002182	0.59	0.14	6.23	0.10	0.01	0.04	0.04	0.04	323.54	584.66
312	EIF3F	3.60E-05	0.002020827	0.48	0.12	7.86	0.06	0.00	0.02	0.03	0.03	1793.64	2877.38
313	CYP4X1	3.63E-05	0.002032672	1.32	0.32	4.17	0.52	0.01	0.23	0.13	0.23	27.08	90.57
314	TMEM255A	3.65E-05	0.002036963	-1.59	0.39	3.12	0.77	0.11	0.24	0.20	0.24	98.37	18.48
315	CORO1C	3.67E-05	0.002044267	-0.65	0.16	7.09	0.13	0.00	0.06	0.03	0.06	2165.85	1068.02

316	MED11	3.72E-05	0.002062369	0.58	0.14	6.53	0.10	0.00	0.05	0.03	0.05	452.40	781.13
317	CISD3	3.80E-05	0.002072502	0.53	0.13	7.72	0.09	0.00	0.04	0.03	0.04	1522.65	2541.02
318	DNAL4	3.78E-05	0.002072502	0.62	0.15	5.52	0.10	0.00	0.03	0.05	0.05	152.70	287.56
319	NPM3	3.81E-05	0.002072502	0.58	0.14	5.78	0.09	0.00	0.03	0.04	0.04	209.36	368.79
320	PPP1R16A	3.81E-05	0.002072502	0.81	0.20	5.90	0.20	0.00	0.09	0.04	0.09	200.39	445.50
321	RPL23A	3.77E-05	0.002072502	0.54	0.13	9.25	0.08	0.00	0.04	0.04	0.04	6915.17	11737.26
322	SLC37A2	3.79E-05	0.002072502	2.34	0.57	9.04	1.66	0.00	0.77	0.04	0.77	3370.21	15218.69
323	SLC51A	3.82E-05	0.002072502	-1.22	0.30	5.07	0.45	0.01	0.20	0.07	0.20	589.62	129.40
324	CDK15	3.85E-05	0.002077467	-2.16	0.52	3.07	1.41	0.09	0.57	0.20	0.57	140.92	17.75
325	RWDD2B	3.85E-05	0.002077467	0.48	0.12	6.89	0.05	0.00	0.01	0.03	0.03	667.72	1088.32
326	C4orf19	3.89E-05	0.00209109	-1.79	0.43	3.10	0.97	0.17	0.28	0.20	0.28	120.19	17.80
327	DICER1	3.94E-05	0.002109253	-0.52	0.13	6.26	0.06	0.00	0.01	0.04	0.04	811.79	474.18
328	ORAI3	3.95E-05	0.002109253	0.49	0.12	7.09	0.07	0.00	0.03	0.03	0.03	821.82	1336.14
329	RPS16	3.96E-05	0.002109253	0.56	0.14	8.23	0.10	0.00	0.05	0.03	0.05	2467.15	4285.50
330	LTBP4	3.98E-05	0.002114735	1.15	0.28	5.66	0.40	0.01	0.18	0.05	0.18	145.00	370.66
331	MRPL10	4.03E-05	0.002131094	0.47	0.12	7.24	0.05	0.00	0.01	0.03	0.03	958.29	1535.48
332	RPL23AP1	4.04E-05	0.002131094	1.14	0.28	3.92	0.40	0.03	0.16	0.15	0.16	20.81	68.31
333	AKAP13	4.14E-05	0.002176773	-0.49	0.12	6.61	0.07	0.00	0.03	0.03	0.03	1111.18	683.69
334	ZDHHC23	4.18E-05	0.002191529	-1.20	0.29	4.29	0.44	0.03	0.18	0.12	0.18	193.51	62.82
335	C9orf3	4.19E-05	0.002192653	-0.50	0.12	6.36	0.06	0.00	0.01	0.03	0.03	864.35	524.78
336	TSEN2	4.25E-05	0.002218245	0.51	0.13	6.49	0.07	0.00	0.02	0.03	0.03	440.84	738.11
337	XYLT2	4.40E-05	0.002288712	0.52	0.13	6.63	0.08	0.00	0.04	0.03	0.04	504.43	850.51
338	IL6ST	4.44E-05	0.00229534	-0.70	0.17	7.79	0.15	0.00	0.07	0.03	0.07	4288.18	2175.36
339	RGS8	4.43E-05	0.00229534	2.39	0.59	3.61	1.74	0.05	0.78	0.17	0.78	6.39	89.96
340	MRPS24	4.45E-05	0.002296247	0.47	0.12	7.43	0.06	0.00	0.03	0.03	0.03	1198.79	1862.97
341	ERBB2	4.60E-05	0.002361872	0.77	0.19	5.01	0.17	0.01	0.07	0.08	0.08	83.37	180.25
342	MZT2B	4.64E-05	0.00237994	0.75	0.18	7.04	0.17	0.00	0.08	0.03	0.08	686.79	1358.59
343	UBXN1	4.74E-05	0.002424282	0.47	0.12	7.37	0.05	0.00	0.01	0.03	0.03	1102.78	1760.03
344	MXRA5	4.84E-05	0.002465824	-1.35	0.33	8.50	0.56	0.00	0.26	0.04	0.26	17108.91	4085.81

345	SSSCA1	4.88E-05	0.00248128	0.61	0.15	6.14	0.11	0.00	0.05	0.04	0.05	296.90	535.04
346	FRMD4A	4.93E-05	0.002498176	-0.91	0.23	5.54	0.26	0.01	0.11	0.05	0.11	633.03	215.71
347	FAM127C	4.96E-05	0.002505278	0.48	0.12	6.72	0.07	0.00	0.03	0.03	0.03	580.00	924.64
348	ADAM19	4.99E-05	0.002512563	-1.24	0.30	4.97	0.47	0.01	0.21	0.08	0.21	417.28	126.86
349	RPL19	5.04E-05	0.002533342	0.59	0.14	10.11	0.08	0.00	0.03	0.05	0.05	15640.99	27775.94
350	CCDC12	5.06E-05	0.002535471	0.57	0.14	6.15	0.10	0.00	0.05	0.04	0.05	310.35	532.06
351	COL4A1	5.08E-05	0.002537207	-0.91	0.22	6.76	0.25	0.00	0.12	0.03	0.12	1923.05	754.92
352	CYP4Z1	5.13E-05	0.002550681	2.08	0.51	2.29	1.33	0.10	0.54	0.26	0.54	3.42	16.11
353	IGFBP7	5.14E-05	0.002550681	0.77	0.19	9.51	0.18	0.00	0.09	0.04	0.09	7987.02	16204.80
354	TMEM169	5.20E-05	0.002572426	-1.60	0.40	3.46	0.79	0.05	0.32	0.18	0.32	136.75	26.46
355	ZBTB38	5.23E-05	0.002582429	-0.47	0.12	7.80	0.06	0.00	0.03	0.03	0.03	3584.96	2249.76
356	BDP1	5.25E-05	0.002587027	-0.47	0.12	7.50	0.06	0.00	0.02	0.03	0.03	2752.50	1645.72
357	SMC1A	5.31E-05	0.002609227	-0.52	0.13	6.21	0.08	0.00	0.03	0.04	0.04	756.13	455.97
358	ANXA3	5.38E-05	0.002628784	1.35	0.33	4.35	0.56	0.02	0.25	0.12	0.25	27.87	113.93
359	MRPS6	5.38E-05	0.002628784	0.53	0.13	7.45	0.09	0.00	0.04	0.03	0.04	1137.24	1951.15
360	C6orf1	5.43E-05	0.002642651	0.76	0.19	6.53	0.18	0.00	0.08	0.03	0.08	401.47	821.05
361	RPL41P2	5.46E-05	0.00264934	0.66	0.16	6.08	0.13	0.01	0.06	0.04	0.06	263.35	514.89
362	GABARAPL1	5.50E-05	0.00266147	-0.55	0.14	7.03	0.09	0.00	0.04	0.03	0.04	1762.85	1029.77
363	RPL32	5.52E-05	0.002666204	0.55	0.14	9.68	0.08	0.00	0.03	0.04	0.04	10533.86	17970.43
364	DDIT4	5.60E-05	0.00269121	0.64	0.16	7.53	0.13	0.00	0.06	0.03	0.06	1128.92	2186.57
365	TADA3	5.60E-05	0.00269121	0.47	0.12	7.92	0.05	0.00	0.02	0.03	0.03	1918.33	3027.87
366	C11orf74	5.81E-05	0.002772684	0.52	0.13	6.10	0.08	0.00	0.04	0.04	0.04	309.19	499.92
367	CD52	5.80E-05	0.002772684	-2.03	0.51	3.61	1.28	0.06	0.55	0.17	0.55	408.96	26.16
368	PSME4	5.89E-05	0.002803145	-0.78	0.19	6.49	0.19	0.00	0.09	0.03	0.09	1357.76	577.20
369	TMEM258	6.05E-05	0.002872376	0.47	0.12	7.79	0.07	0.00	0.03	0.03	0.03	1683.25	2701.46
370	KLHL21	6.28E-05	0.002968668	0.69	0.17	6.96	0.15	0.00	0.07	0.03	0.07	613.59	1249.70
371	MATN2	6.30E-05	0.002968668	1.16	0.29	6.74	0.42	0.00	0.20	0.03	0.20	407.24	1136.49
372	UGGT1	6.29E-05	0.002968668	-0.47	0.12	6.55	0.05	0.00	0.01	0.03	0.03	1031.24	641.55
373	PPP1R3G	6.32E-05	0.002970529	0.83	0.21	6.16	0.21	0.00	0.10	0.04	0.10	248.29	586.00

374	TPH1	6.36E-05	0.002981677	-3.14	0.78	1.64	3.06	0.12	1.36	0.32	1.36	321.49	2.48
375	SEC16A	6.45E-05	0.003016078	-0.54	0.14	7.82	0.09	0.00	0.04	0.03	0.04	3853.31	2281.25
376	CLRN1	6.54E-05	0.003046637	2.12	0.53	6.50	1.39	0.00	0.67	0.03	0.67	323.77	1127.28
377	HOXA7	6.55E-05	0.003046637	0.69	0.17	5.15	0.13	0.00	0.05	0.07	0.07	103.99	200.38
378	KCNK2	6.58E-05	0.003052252	0.86	0.21	8.09	0.23	0.00	0.11	0.03	0.11	1811.56	3998.08
379	EIF2D	6.81E-05	0.003149296	0.47	0.12	6.47	0.05	0.00	0.01	0.03	0.03	445.02	711.68
380	AHCY	6.88E-05	0.003174223	0.47	0.12	7.89	0.06	0.00	0.02	0.03	0.03	1846.72	2966.65
381	AP1G1	6.96E-05	0.003193652	-0.47	0.12	6.95	0.07	0.00	0.03	0.03	0.03	1527.47	961.37
382	MAPKAPK3	6.96E-05	0.003193652	0.73	0.18	7.13	0.17	0.00	0.08	0.03	0.08	740.50	1487.19
383	HIST2H2BA	7.10E-05	0.003241189	0.95	0.24	4.58	0.28	0.02	0.11	0.10	0.11	51.23	122.23
384	PAM16	7.09E-05	0.003241189	0.53	0.13	5.99	0.07	0.00	0.02	0.04	0.04	264.74	448.24
385	DHX29	7.25E-05	0.003295586	-0.47	0.12	7.94	0.07	0.00	0.03	0.03	0.03	4325.88	2560.23
386	FAM53B	7.26E-05	0.003295586	0.51	0.13	7.88	0.08	0.00	0.04	0.03	0.04	1818.19	2967.55
387	ADAMTSL5	7.31E-05	0.003303646	1.40	0.35	2.82	0.48	0.08	0.07	0.22	0.22	4.98	23.62
388	TET2	7.31E-05	0.003303646	-0.48	0.12	7.68	0.07	0.00	0.02	0.03	0.03	3206.53	1991.85
389	SPON2	7.34E-05	0.003306389	0.90	0.23	4.88	0.25	0.00	0.12	0.08	0.12	69.43	163.25
390	VASH1	7.47E-05	0.003354935	0.49	0.12	6.20	0.08	0.00	0.04	0.04	0.04	332.99	556.65
391	CUEDC1	7.53E-05	0.003377207	0.58	0.15	5.70	0.09	0.01	0.03	0.05	0.05	187.79	341.79
392	NLRX1	7.63E-05	0.003412938	0.54	0.14	5.82	0.08	0.00	0.03	0.04	0.04	220.69	380.62
393	CHCHD5	7.90E-05	0.003520931	0.61	0.15	5.89	0.12	0.00	0.05	0.04	0.05	233.29	416.52
394	ACP5	8.01E-05	0.003562024	-1.83	0.46	4.71	1.05	0.02	0.50	0.09	0.50	641.97	94.74
395	SGPL1	8.06E-05	0.003576653	-0.55	0.14	5.76	0.09	0.00	0.04	0.04	0.04	513.32	287.22
396	AGBL5	8.09E-05	0.003580483	0.53	0.13	6.68	0.09	0.00	0.04	0.03	0.04	548.78	891.78
397	RPL38	8.26E-05	0.003646099	0.51	0.13	9.09	0.07	0.00	0.03	0.04	0.04	6070.77	9901.86
398	RASIP1	8.38E-05	0.003688181	1.16	0.29	5.48	0.42	0.00	0.20	0.05	0.20	97.43	332.51
399	MACROD1	8.57E-05	0.003766387	0.89	0.23	4.97	0.25	0.01	0.11	0.08	0.11	75.67	179.46
400	ZDHHC2	8.62E-05	0.003776067	0.49	0.12	6.25	0.07	0.00	0.03	0.04	0.04	360.34	573.65
401	EEF1A1P5	8.67E-05	0.003789634	0.49	0.12	6.82	0.08	0.00	0.03	0.03	0.03	623.41	1022.24
402	MRPL53	8.72E-05	0.003803518	0.50	0.13	6.55	0.08	0.00	0.04	0.03	0.04	473.89	780.98

403	MYL6B	8.78E-05	0.003809141	0.59	0.15	6.50	0.11	0.00	0.05	0.03	0.05	420.54	768.51
404	TOMM7	8.78E-05	0.003809141	0.59	0.15	8.95	0.11	0.00	0.05	0.04	0.05	5058.10	8842.81
405	ADH1A	8.83E-05	0.003814667	1.31	0.34	3.75	0.55	0.05	0.22	0.16	0.22	17.07	60.71
406	SLC31A2	8.84E-05	0.003814667	-1.04	0.26	6.77	0.34	0.00	0.17	0.03	0.17	2539.92	733.23
407	GPR153	8.86E-05	0.00381685	-2.01	0.51	4.10	1.28	0.03	0.60	0.14	0.60	701.86	45.02
408	EXPH5	9.07E-05	0.003887061	-1.18	0.30	5.33	0.44	0.01	0.21	0.06	0.21	669.64	172.33
409	PYCR2	9.07E-05	0.003887061	0.45	0.12	7.47	0.06	0.00	0.03	0.03	0.03	1218.27	1939.85
410	NECAB3	9.13E-05	0.003902514	0.46	0.12	7.08	0.06	0.00	0.02	0.03	0.03	826.71	1315.80
411	RPS8	9.19E-05	0.003919882	0.58	0.15	10.36	0.11	0.00	0.05	0.05	0.05	21374.90	35707.54
412	ZNF135	9.28E-05	0.003945721	0.84	0.22	5.00	0.23	0.01	0.10	0.08	0.10	81.94	181.36
413	TMEM256	9.32E-05	0.003956553	0.46	0.12	6.82	0.05	0.00	0.02	0.03	0.03	637.74	1010.81
414	ELK3	9.60E-05	0.004055304	-0.66	0.17	5.18	0.12	0.00	0.04	0.07	0.07	302.69	158.15
415	POU3F1	9.59E-05	0.004055304	1.42	0.36	3.79	0.64	0.04	0.28	0.16	0.28	17.50	64.24
416	KRT19	9.66E-05	0.004069521	1.68	0.43	2.66	0.89	0.06	0.39	0.23	0.39	4.22	23.57
417	NAA38	9.81E-05	0.00412181	0.52	0.13	7.12	0.09	0.00	0.04	0.03	0.04	840.45	1384.24
418	FAM175A	9.88E-05	0.004143604	0.58	0.15	6.23	0.11	0.00	0.05	0.04	0.05	321.55	586.79
419	SLC25A11	9.93E-05	0.004154134	0.45	0.12	7.17	0.06	0.00	0.03	0.03	0.03	915.86	1442.77
420	ISY1	9.99E-05	0.004168459	0.53	0.14	6.08	0.05	0.01	0.00	0.04	0.04	286.86	487.91
421	MACC1	0.000101004	0.004204755	2.01	0.52	5.14	1.29	0.01	0.63	0.07	0.63	43.27	348.10
422	GCLM	0.000101793	0.004227549	-0.56	0.14	7.28	0.10	0.00	0.05	0.03	0.05	2272.32	1335.41
423	DCPS	0.000102689	0.00425467	0.50	0.13	6.14	0.06	0.00	0.01	0.04	0.04	312.28	513.88
424	TFPI	0.000103372	0.004272858	-1.45	0.37	5.62	0.67	0.00	0.33	0.05	0.33	992.42	234.76
425	PAPOLA	0.000105203	0.004338318	-0.45	0.12	7.50	0.05	0.00	0.02	0.03	0.03	2667.21	1662.46
426	C19orf33	0.000105486	0.004339766	1.49	0.39	3.50	0.71	0.07	0.29	0.17	0.29	12.43	50.24
427	HOXA6	0.000108063	0.004434452	0.58	0.15	5.57	0.07	0.00	0.01	0.05	0.05	164.98	297.19
428	PLEKHO1	0.000108293	0.004434452	0.75	0.19	6.92	0.18	0.00	0.09	0.03	0.09	574.83	1217.25
429	HDDC2	0.000110808	0.004526851	0.45	0.12	7.33	0.05	0.00	0.02	0.03	0.03	1091.54	1679.88
430	RP11-295K3.1	0.000112507	0.004585578	0.72	0.19	6.80	0.17	0.00	0.08	0.03	0.08	520.43	1075.03
431	VPS51	0.000113827	0.004628622	0.54	0.14	7.21	0.09	0.00	0.04	0.03	0.04	922.98	1527.94

432	SCAMP3	0.000114838	0.004658922	0.46	0.12	7.03	0.07	0.00	0.03	0.03	0.03	809.20	1252.34
433	TCIRG1	0.000116002	0.004695287	1.34	0.35	6.65	0.58	0.00	0.29	0.03	0.29	361.51	1062.91
434	KDM5C	0.000117576	0.004748013	-0.54	0.14	6.74	0.09	0.00	0.04	0.03	0.04	1330.93	771.94
435	CCDC88A	0.000118323	0.004764764	-0.46	0.12	7.64	0.05	0.00	0.02	0.03	0.03	3006.79	1905.51
436	CHRNA7	0.00011856	0.004764764	-3.70	0.96	3.88	4.41	0.13	2.09	0.15	2.09	3097.40	74.48
437	LRRC8E	0.000118806	0.004764764	1.08	0.28	4.82	0.38	0.01	0.18	0.09	0.18	67.56	157.74
438	RARRES2	0.000120084	0.004804995	1.24	0.32	9.77	0.49	0.00	0.25	0.05	0.25	9724.78	23317.71
439	ZNF92	0.00012079	0.004822242	0.56	0.14	6.98	0.10	0.00	0.05	0.03	0.05	700.68	1221.52
440	SELE	0.000121126	0.004824679	-2.19	0.57	3.07	1.55	0.20	0.58	0.20	0.58	153.93	18.31
441	ADGRB2	0.000123044	0.004878882	-1.23	0.32	4.21	0.48	0.04	0.21	0.13	0.21	195.96	60.15
442	GOLGA7B	0.000122849	0.004878882	-2.22	0.58	4.95	1.59	0.02	0.78	0.08	0.78	1470.32	110.55
443	BCAR3	0.00012345	0.00488395	0.47	0.12	6.42	0.06	0.00	0.03	0.03	0.03	434.35	679.39
444	CEBPG	0.000123951	0.004892712	-0.45	0.12	6.88	0.06	0.00	0.02	0.03	0.03	1411.42	900.52
445	AKIRIN2	0.0001278	0.005033316	-0.51	0.13	6.70	0.08	0.00	0.04	0.03	0.04	1294.41	737.70
446	AFF4	0.000128311	0.005037917	-0.48	0.12	7.31	0.07	0.00	0.04	0.03	0.04	2239.01	1380.24
447	THAP7	0.000128492	0.005037917	0.46	0.12	6.52	0.07	0.00	0.03	0.03	0.03	468.95	753.35
448	PCDHGA9	0.000128849	0.005040629	-1.05	0.27	5.85	0.35	0.01	0.17	0.04	0.17	916.42	298.37
449	SLC44A5	0.000130143	0.005079924	-2.78	0.73	4.57	2.50	0.03	1.23	0.10	1.23	1133.72	97.12
450	RAB11FIP1P1	0.000130697	0.005090226	-0.55	0.15	5.66	0.09	0.00	0.03	0.05	0.05	456.17	260.09
451	FNTB	0.000135138	0.005230472	-0.76	0.20	5.40	0.19	0.00	0.09	0.06	0.09	467.43	192.88
452	HIST2H2AC	0.000135194	0.005230472	0.52	0.14	6.99	0.09	0.00	0.04	0.03	0.04	745.50	1227.22
453	PCK2	0.00013511	0.005230472	-0.56	0.15	5.70	0.10	0.01	0.04	0.05	0.05	491.54	269.60
454	ZCCHC2	0.000136242	0.005259436	-0.70	0.18	6.21	0.16	0.01	0.07	0.04	0.07	895.30	448.77
455	ZMAT5	0.000138035	0.005316944	0.45	0.12	7.06	0.06	0.00	0.02	0.03	0.03	825.56	1290.87
456	ZCCHC14	0.000139669	0.005368079	-0.47	0.12	7.35	0.07	0.00	0.04	0.03	0.04	2301.26	1441.13
457	PNMA2	0.000140579	0.005379453	1.11	0.29	3.43	0.32	0.02	0.10	0.18	0.18	12.92	39.95
458	TRMT112	0.000140466	0.005379453	0.46	0.12	8.46	0.06	0.00	0.02	0.04	0.04	3274.89	5205.07
459	GHDC	0.000142249	0.005431489	0.62	0.16	6.59	0.12	0.00	0.06	0.03	0.06	469.01	837.21
460	ABCD2	0.000145732	0.005552376	-1.48	0.39	6.28	0.71	0.00	0.36	0.03	0.36	2337.34	460.31

461	ABCA6	0.000148151	0.005632301	0.63	0.17	5.82	0.13	0.00	0.06	0.04	0.06	204.37	397.45
462	CNEP1R1	0.000148878	0.005635501	-0.52	0.14	5.83	0.06	0.00	0.01	0.04	0.04	530.06	307.30
463	SAR1B	0.000148613	0.005635501	-0.44	0.12	7.44	0.05	0.00	0.02	0.03	0.03	2445.05	1581.38
464	DDX3X	0.000149257	0.00563765	-0.52	0.14	9.09	0.09	0.00	0.05	0.04	0.05	13416.84	8154.27
465	SCUBE3	0.000150089	0.005656894	0.83	0.22	6.47	0.22	0.00	0.11	0.03	0.11	386.06	784.16
466	TRPS1	0.000151248	0.005688359	-0.54	0.14	6.55	0.10	0.01	0.04	0.03	0.04	1076.55	641.77
467	C8orf82	0.000153938	0.00577714	0.62	0.16	5.98	0.13	0.00	0.06	0.04	0.06	254.79	456.49
468	TPCN1	0.000154756	0.005795431	0.48	0.13	6.14	0.05	0.00	0.01	0.04	0.04	318.15	512.56
469	FAM177A1	0.000155379	0.005806341	-0.46	0.12	6.50	0.06	0.00	0.02	0.03	0.03	959.12	612.24
470	HERC2P8	0.000155747	0.005807712	-1.23	0.33	3.41	0.39	0.07	0.06	0.18	0.18	95.68	24.03
471	KIRREL	0.000158544	0.005899465	0.50	0.13	8.01	0.08	0.00	0.04	0.03	0.04	2100.16	3363.48
472	ADAM10	0.000159409	0.005906545	-0.44	0.12	6.96	0.06	0.00	0.03	0.03	0.03	1559.71	970.89
473	MST1	0.000159074	0.005906545	0.68	0.18	5.17	0.14	0.01	0.05	0.07	0.07	106.17	205.63
474	ANKRD39	0.000162523	0.006009224	0.47	0.12	6.69	0.07	0.00	0.03	0.03	0.03	558.48	900.55
475	GSKIP	0.000163301	0.006025302	-0.57	0.15	5.79	0.11	0.00	0.05	0.04	0.05	540.43	298.12
476	C12orf10	0.000163767	0.006029803	0.44	0.12	6.83	0.05	0.00	0.02	0.03	0.03	650.39	1019.14
477	TCEAL3	0.000166433	0.006115102	0.47	0.12	7.37	0.07	0.00	0.04	0.03	0.04	1104.08	1767.51
478	RPL37A	0.000169638	0.006219817	0.59	0.16	10.65	0.09	0.00	0.04	0.06	0.06	27619.41	47899.66
479	CEACAM1	0.000171267	0.00626643	-0.69	0.18	5.34	0.16	0.01	0.07	0.06	0.07	375.47	187.37
480	KLHL2	0.000173363	0.006322391	-0.56	0.15	6.97	0.10	0.00	0.05	0.03	0.05	1663.44	979.72
481	TMEM138	0.00017372	0.006322391	0.57	0.15	7.41	0.11	0.00	0.05	0.03	0.05	1139.95	1861.57
482	TPMT	0.000173878	0.006322391	-0.54	0.14	7.15	0.10	0.00	0.05	0.03	0.05	2039.07	1155.97
483	MFGE8	0.000175211	0.006344509	0.75	0.20	7.34	0.19	0.00	0.10	0.03	0.10	875.78	1856.13
484	TMEM33	0.000174917	0.006344509	-0.43	0.12	7.06	0.04	0.00	0.01	0.03	0.03	1674.08	1070.67
485	DNAJC25	0.000177044	0.006388531	-0.44	0.12	6.65	0.05	0.00	0.02	0.03	0.03	1100.72	711.70
486	HSD17B4	0.00017752	0.006388531	-0.49	0.13	8.12	0.08	0.00	0.04	0.03	0.04	5030.84	3122.20
487	RPS10	0.000177177	0.006388531	0.51	0.14	9.60	0.07	0.00	0.03	0.04	0.04	10017.46	16496.12
488	CDPF1	0.000179317	0.006439987	0.57	0.15	5.83	0.11	0.01	0.05	0.04	0.05	218.85	390.38
489	RPL28	0.000181971	0.006521915	0.47	0.13	8.81	0.06	0.00	0.02	0.04	0.04	4650.25	7457.52

490	EHMT2	0.000183091	0.006525372	0.44	0.12	7.27	0.05	0.00	0.01	0.03	0.03	1017.83	1570.68
491	FRYL	0.000183184	0.006525372	-0.44	0.12	7.34	0.05	0.00	0.02	0.03	0.03	2221.33	1426.57
492	PCBP2	0.000182993	0.006525372	-0.61	0.16	9.30	0.12	0.00	0.06	0.04	0.06	18163.70	9917.93
493	PTS	0.00018361	0.006527276	-0.87	0.23	7.26	0.25	0.00	0.13	0.03	0.13	3364.21	1234.73
494	MYL9	0.000184432	0.00654324	0.74	0.20	10.10	0.18	0.00	0.09	0.05	0.09	14196.79	29276.83
495	FKBP9	0.000188629	0.006678603	0.50	0.13	7.39	0.08	0.00	0.04	0.03	0.04	1115.76	1826.96
496	ZAP70	0.000189339	0.006690225	-1.63	0.44	2.53	0.88	0.22	0.23	0.24	0.24	77.27	9.11
497	MKX	0.000191097	0.00673494	1.37	0.37	4.79	0.62	0.02	0.31	0.09	0.31	52.20	175.09
498	RWDD4	0.000191757	0.00673494	0.46	0.12	8.13	0.07	0.00	0.04	0.03	0.04	2478.03	3733.97
499	SLC4A2	0.000191403	0.00673494	0.66	0.18	6.14	0.15	0.00	0.07	0.04	0.07	276.45	551.18
500	ALKBH7	0.000192517	0.006748095	0.66	0.18	6.35	0.15	0.00	0.07	0.03	0.07	349.69	671.67
501	PCOLCE	0.000194269	0.006795934	1.37	0.37	5.66	0.62	0.00	0.32	0.05	0.32	137.75	394.11
502	PMVK	0.000197423	0.006892488	0.54	0.15	6.81	0.10	0.00	0.05	0.03	0.05	617.38	1025.38
503	NATD1	0.000198032	0.00689511	0.45	0.12	6.42	0.06	0.00	0.02	0.03	0.03	439.46	674.38
504	RPL21P16	0.000198333	0.00689511	0.58	0.15	7.99	0.11	0.00	0.05	0.03	0.05	1899.17	3402.46
505	SAC3D1	0.000198678	0.00689511	0.72	0.19	5.28	0.17	0.01	0.08	0.06	0.08	113.81	235.50
506	DYNLT3	0.000200264	0.006936406	0.67	0.18	7.55	0.15	0.00	0.08	0.03	0.08	1163.72	2250.22
507	CX3CR1	0.000203089	0.007006554	1.97	0.53	5.80	1.29	0.01	0.66	0.04	0.66	74.81	686.13
508	PARD3B	0.000202871	0.007006554	0.47	0.13	6.32	0.07	0.00	0.03	0.03	0.03	401.72	616.13
509	WBP1	0.000204848	0.007053369	0.48	0.13	7.16	0.08	0.00	0.04	0.03	0.04	892.46	1431.17
510	C19orf70	0.000206186	0.007085533	0.45	0.12	6.58	0.07	0.00	0.03	0.03	0.03	509.83	797.10
511	CSRP1	0.000206624	0.007086663	0.97	0.26	7.07	0.31	0.00	0.16	0.03	0.16	589.73	1522.83
512	GPRASP1	0.000207236	0.007093787	0.52	0.14	8.16	0.09	0.00	0.05	0.03	0.05	2340.04	3951.24
513	REPIN1	0.000208412	0.00712013	0.43	0.12	7.54	0.05	0.00	0.02	0.03	0.03	1340.74	2080.88
514	SLC39A1	0.000212887	0.00725885	0.44	0.12	8.17	0.05	0.00	0.02	0.03	0.03	2546.28	3877.19
515	TYSND1	0.000216044	0.007352219	0.44	0.12	6.78	0.06	0.00	0.02	0.03	0.03	622.89	975.04
516	TMEM176A	0.000216491	0.007353153	1.29	0.35	5.83	0.55	0.01	0.29	0.04	0.29	136.79	488.49
517	RP11-327P2.7	0.000218734	0.007400649	-2.46	0.67	1.15	2.02	0.13	0.93	0.36	0.93	85.19	1.94
518	RPS21	0.000218644	0.007400649	0.52	0.14	8.68	0.09	0.00	0.05	0.04	0.05	4076.64	6589.85

519	NFKBIL1	0.0002194	0.007408875	0.52	0.14	5.75	0.08	0.00	0.03	0.04	0.04	206.43	353.75
520	EMILIN3	0.000221024	0.00744936	-2.88	0.78	3.22	2.77	0.17	1.28	0.19	1.28	649.55	21.81
521	SAMD11	0.000221506	0.007451286	1.32	0.36	4.62	0.59	0.06	0.25	0.10	0.25	48.69	142.90
522	PIP5K1B	0.000224354	0.007532617	-0.91	0.25	6.24	0.28	0.00	0.14	0.04	0.14	1243.43	439.77
523	PABPC1P4	0.000224889	0.007536162	1.84	0.50	2.88	1.14	0.13	0.47	0.22	0.47	7.13	30.66
524	VEGFB	0.000227516	0.007609629	0.62	0.17	7.30	0.13	0.00	0.07	0.03	0.07	895.43	1724.47
525	TMEM2	0.000228722	0.007635408	-0.80	0.22	5.36	0.21	0.01	0.10	0.06	0.10	430.86	189.55
526	SUFU	0.000230192	0.007669849	-0.54	0.15	5.59	0.08	0.00	0.03	0.05	0.05	420.30	242.35
527	FAM196A	0.000231832	0.007709835	-1.53	0.42	3.01	0.79	0.09	0.33	0.21	0.33	117.69	16.02
528	C11orf71	0.000233235	0.007741817	0.72	0.20	5.14	0.18	0.00	0.09	0.07	0.09	110.86	199.68
529	HS6ST3	0.000235743	0.007770491	0.90	0.24	7.89	0.27	0.00	0.14	0.03	0.14	1543.73	3322.93
530	NSG1	0.000235929	0.007770491	-1.50	0.41	5.53	0.75	0.23	0.17	0.05	0.17	903.59	218.68
531	PAMR1	0.000235451	0.007770491	1.51	0.41	6.66	0.76	0.00	0.40	0.03	0.40	416.89	1137.90
532	RIDA	0.000236316	0.007770491	-0.74	0.20	6.98	0.18	0.00	0.10	0.03	0.10	2030.66	959.33
533	ZNF737	0.000235577	0.007770491	0.60	0.16	5.78	0.12	0.00	0.06	0.04	0.06	206.87	371.72
534	EFS	0.000240842	0.007904492	1.02	0.28	3.82	0.29	0.03	0.10	0.16	0.16	20.28	58.81
535	SMOC2	0.000241484	0.007910743	1.53	0.42	5.76	0.79	0.00	0.42	0.04	0.42	105.81	517.27
536	SMIM4	0.000242469	0.007928207	0.57	0.15	6.81	0.11	0.00	0.05	0.03	0.05	601.60	1037.63
537	ROGDI	0.00024446	0.007978421	0.46	0.13	6.40	0.06	0.00	0.02	0.03	0.03	424.57	661.82
538	KALRN	0.000245588	0.007988993	-0.79	0.22	6.76	0.21	0.00	0.11	0.03	0.11	1740.51	768.85
539	RPL18	0.000245696	0.007988993	0.51	0.14	9.85	0.06	0.00	0.02	0.05	0.05	12693.95	21193.27
540	KIAA2026	0.000246215	0.007991056	-0.43	0.12	7.49	0.06	0.00	0.02	0.03	0.03	2590.63	1644.79
541	FERMT1	0.000246936	0.007999615	1.03	0.28	7.47	0.36	0.00	0.19	0.03	0.19	945.37	2276.86
542	ABHD3	0.00025072	0.008107219	-0.63	0.17	5.27	0.11	0.01	0.04	0.06	0.06	329.56	174.55
543	RNLS	0.000253249	0.008153675	-0.46	0.12	6.31	0.06	0.00	0.03	0.03	0.03	800.62	509.82
544	TMEM101	0.000252693	0.008153675	0.48	0.13	5.95	0.05	0.00	0.01	0.04	0.04	261.17	422.65
545	ZNF781	0.000253552	0.008153675	0.68	0.19	5.30	0.14	0.02	0.04	0.06	0.06	117.64	236.15
546	SPACA9	0.000255724	0.008208465	0.54	0.15	6.26	0.10	0.00	0.05	0.04	0.05	351.41	592.14
547	CYS1	0.000259344	0.008294286	1.13	0.31	3.49	0.42	0.05	0.17	0.18	0.18	14.02	44.37

548	FAM50B	0.000259242	0.008294286	1.28	0.35	5.62	0.55	0.01	0.29	0.05	0.29	146.93	360.73
549	IRGQ	0.000259876	0.008296162	-0.43	0.12	6.58	0.05	0.00	0.01	0.03	0.03	1026.85	662.24
550	PLCD1	0.000260895	0.008313539	0.92	0.25	5.27	0.29	0.01	0.14	0.06	0.14	109.58	243.63
551	TFAP4	0.000262422	0.008347025	0.72	0.20	5.11	0.15	0.02	0.04	0.07	0.07	93.92	196.59
552	C4orf32	0.000266148	0.008418089	-0.57	0.16	7.28	0.11	0.00	0.06	0.03	0.06	2455.23	1308.99
553	CGNL1	0.000267058	0.008418089	-1.04	0.29	6.81	0.37	0.00	0.20	0.03	0.20	2659.21	762.43
554	CIPC	0.000266743	0.008418089	-0.55	0.15	5.48	0.08	0.00	0.03	0.05	0.05	390.40	216.22
555	MIF4GD	0.000266552	0.008418089	0.50	0.14	5.85	0.07	0.00	0.02	0.04	0.04	236.52	387.07
556	ZFYVE26	0.000266586	0.008418089	-0.44	0.12	7.27	0.05	0.00	0.01	0.03	0.03	2057.90	1323.55
557	LHFP	0.000267668	0.008422177	0.75	0.20	7.01	0.19	0.00	0.10	0.03	0.10	629.86	1342.99
558	GKN1	0.000268195	0.008423633	2.12	0.58	3.75	1.52	0.04	0.77	0.16	0.77	14.41	77.24
559	CSGALNACT2	0.00027037	0.008466403	-0.43	0.12	6.71	0.05	0.00	0.02	0.03	0.03	1161.41	760.01
560	LAMA3	0.000271006	0.008466403	-1.10	0.30	3.59	0.41	0.04	0.18	0.17	0.18	87.14	31.88
561	PITX1	0.000270982	0.008466403	-2.22	0.61	1.25	1.67	0.12	0.77	0.36	0.77	32.69	2.63
562	HIST2H3DP1	0.000277026	0.008639066	2.00	0.55	3.11	1.36	0.05	0.68	0.20	0.68	8.93	35.88
563	EIF3I	0.000277569	0.008640629	0.43	0.12	7.91	0.06	0.00	0.03	0.03	0.03	2038.58	2991.34
564	RP11-411G2.1	0.000284828	0.008850886	1.39	0.38	2.61	0.53	0.11	0.10	0.24	0.24	4.62	18.89
565	DBF4	0.000285362	0.008851766	-0.66	0.18	5.18	0.12	0.01	0.04	0.07	0.07	315.63	158.22
566	MRGPRF	0.000291598	0.009029242	1.35	0.37	4.25	0.63	0.02	0.32	0.13	0.32	28.30	108.01
567	NOX4	0.000292842	0.009051763	-2.43	0.67	2.72	2.02	0.09	1.00	0.23	1.00	531.13	9.47
568	HOMER1	0.000293955	0.00907017	-1.99	0.55	3.26	1.36	0.07	0.66	0.19	0.66	266.73	18.80
569	FAM89B	0.000297299	0.009157241	0.57	0.16	6.14	0.11	0.00	0.05	0.04	0.05	312.50	526.46
570	NTPCR	0.000299913	0.009200026	0.42	0.12	7.20	0.05	0.00	0.02	0.03	0.03	955.81	1463.47
571	SNAPIN	0.000299326	0.009200026	0.42	0.12	7.16	0.05	0.00	0.03	0.03	0.03	937.56	1406.11
572	TAGLN	0.000300263	0.009200026	1.13	0.31	8.87	0.44	0.00	0.24	0.04	0.24	3627.57	9482.62
573	BTG2	0.000302683	0.009209761	1.57	0.43	8.23	0.84	0.00	0.45	0.03	0.45	1168.28	6320.07
574	DVL1	0.00030193	0.009209761	0.66	0.18	6.18	0.15	0.00	0.08	0.04	0.08	295.61	565.20
575	SMAD2	0.000302097	0.009209761	-0.43	0.12	8.07	0.06	0.00	0.03	0.03	0.03	4527.63	2972.02
576	YEATS4	0.000302554	0.009209761	0.65	0.18	6.15	0.14	0.00	0.08	0.04	0.08	298.15	548.29

577	DDRGK1	0.000303351	0.009213634	0.42	0.12	7.54	0.06	0.00	0.03	0.03	0.03	1352.16	2086.19
578	LOXL1	0.000304387	0.009213634	0.78	0.22	4.91	0.20	0.03	0.07	0.08	0.08	80.83	162.71
579	NUDT7	0.000304359	0.009213634	0.45	0.13	6.23	0.06	0.00	0.02	0.04	0.04	357.60	562.92
580	HERC2P5	0.000305336	0.009226409	-1.21	0.33	3.18	0.38	0.07	0.07	0.20	0.20	74.94	19.06
581	ARF6	0.000306528	0.009246488	-0.43	0.12	8.09	0.06	0.00	0.03	0.03	0.03	4770.53	3000.00
582	CH507-9B2.3	0.000308046	0.009260388	0.61	0.17	7.30	0.13	0.00	0.07	0.03	0.07	973.53	1713.14
583	SLC2A4RG	0.000307861	0.009260388	0.57	0.16	5.47	0.11	0.01	0.05	0.05	0.05	155.91	273.02
584	ZNF280C	0.000309618	0.00929171	-0.54	0.15	5.65	0.07	0.01	0.01	0.05	0.05	438.18	256.52
585	EEF1AKMT1	0.000312395	0.009346271	0.53	0.15	5.62	0.08	0.01	0.03	0.05	0.05	181.53	312.43
586	ZDHHC15	0.000312502	0.009346271	-0.46	0.13	6.83	0.06	0.01	0.02	0.03	0.03	1343.54	857.98
587	RPL13	0.000313075	0.009347454	0.51	0.14	9.95	0.08	0.00	0.03	0.05	0.05	14065.84	23576.76
588	RPS25	0.000313952	0.009357706	0.53	0.15	9.59	0.10	0.00	0.05	0.04	0.05	9909.71	16551.93
589	FRY	0.000315934	0.009384169	-0.99	0.27	4.04	0.33	0.03	0.15	0.14	0.15	152.98	47.91
590	GBAS	0.000315498	0.009384169	0.42	0.12	7.57	0.05	0.00	0.02	0.03	0.03	1404.94	2116.65
591	SPNS3	0.000316447	0.009384169	1.43	0.40	2.98	0.70	0.08	0.30	0.21	0.30	7.79	29.08
592	CBL	0.000317689	0.009405105	-0.61	0.17	6.49	0.13	0.00	0.07	0.03	0.07	1165.32	594.37
593	COA4	0.000320387	0.009468965	0.41	0.12	7.28	0.05	0.00	0.02	0.03	0.03	1072.16	1577.74
594	STXBP6	0.000322365	0.009511398	-2.48	0.69	3.39	2.12	0.13	1.02	0.18	1.02	359.46	26.36
595	LIPA	0.000323389	0.009525576	-0.71	0.20	7.67	0.17	0.00	0.09	0.03	0.09	3942.87	1923.08
596	MTX3	0.000325932	0.009584355	-0.42	0.12	7.71	0.05	0.00	0.02	0.03	0.03	3188.27	2052.56
597	TOP1MT	0.000327635	0.009618322	0.58	0.16	5.57	0.12	0.00	0.06	0.05	0.06	173.11	299.81
598	ITIH3	0.000328825	0.009637103	1.15	0.32	4.48	0.45	0.02	0.22	0.11	0.22	35.46	122.56
599	PEAR1	0.000333055	0.009744768	0.77	0.21	4.61	0.16	0.01	0.05	0.10	0.10	54.84	119.73
600	CA5B	0.000335409	0.009797289	-0.85	0.24	4.39	0.25	0.01	0.12	0.12	0.12	168.51	71.63
601	SEMA6D	0.000338726	0.00987772	-1.77	0.49	4.38	1.08	0.07	0.52	0.12	0.52	687.41	59.49
602	SOX9	0.000339375	0.009880219	1.30	0.36	4.65	0.58	0.01	0.30	0.10	0.30	38.16	157.82
603	REEP6	0.000340966	0.009910054	0.90	0.25	6.22	0.28	0.00	0.15	0.04	0.15	301.39	619.05
604	C17orf58	0.000343072	0.009932658	0.49	0.14	5.94	0.07	0.00	0.03	0.04	0.04	264.56	423.39
605	EXOSC5	0.000343443	0.009932658	0.54	0.15	5.54	0.07	0.00	0.02	0.05	0.05	165.66	285.06

606	STK32C	0.000342518	0.009932658	0.53	0.15	5.81	0.10	0.01	0.05	0.04	0.05	222.37	379.83
607	ARHGAP32	0.000346679	0.010009728	-0.47	0.13	8.21	0.08	0.00	0.04	0.03	0.04	5384.90	3437.15
608	PAFAH1B3	0.000347391	0.010013771	0.61	0.17	5.25	0.09	0.01	0.02	0.06	0.06	119.45	217.88
609	ZNF805	0.000348121	0.010018333	-0.42	0.12	6.63	0.06	0.00	0.03	0.03	0.03	1087.84	705.12
610	CHPT1	0.000349722	0.010047925	-0.54	0.15	7.30	0.10	0.00	0.05	0.03	0.05	2367.99	1355.20
611	CARD8	0.000350979	0.010067537	-0.43	0.12	7.00	0.05	0.00	0.02	0.03	0.03	1562.78	1015.28
612	TMEM223	0.000353006	0.010109129	0.49	0.14	5.79	0.05	0.00	0.01	0.04	0.04	223.41	361.63
613	RANGRF	0.000354666	0.010140104	0.45	0.13	6.14	0.05	0.00	0.01	0.04	0.04	324.57	511.23
614	HCG4	0.000355287	0.010141304	1.53	0.43	3.45	0.81	0.07	0.37	0.18	0.37	12.96	48.19
615	IRAK1	0.000357644	0.010158273	0.43	0.12	6.77	0.06	0.00	0.03	0.03	0.03	633.98	960.82
616	RPL21	0.000358153	0.010158273	0.47	0.13	9.22	0.06	0.00	0.03	0.04	0.04	7091.86	11214.19
617	RPS15	0.0003582	0.010158273	0.45	0.13	8.91	0.05	0.00	0.01	0.04	0.04	5185.32	8112.25
618	SNRPE	0.000356741	0.010158273	0.42	0.12	6.78	0.04	0.00	0.01	0.03	0.03	631.63	966.62
619	AKR7A2	0.000360042	0.010161268	0.46	0.13	7.47	0.07	0.00	0.04	0.03	0.04	1253.60	1944.08
620	FST	0.000360045	0.010161268	-1.83	0.51	3.75	1.16	0.04	0.59	0.16	0.59	409.59	31.07
621	RPL29	0.000359356	0.010161268	0.53	0.15	9.53	0.10	0.00	0.05	0.04	0.05	9192.64	15567.42
622	SVEP1	0.000362043	0.010201223	1.32	0.37	7.01	0.60	0.00	0.33	0.03	0.33	535.40	1530.47
623	BCAP29	0.000363031	0.010212662	-0.46	0.13	6.96	0.07	0.00	0.04	0.03	0.04	1581.49	966.28
624	RPL11	0.000365649	0.010269815	0.50	0.14	9.83	0.08	0.00	0.04	0.05	0.05	12928.72	20843.66
625	CAMK1D	0.000366646	0.010281327	-0.83	0.23	5.35	0.24	0.01	0.12	0.06	0.12	429.86	190.40
626	STRADB	0.000367748	0.010295767	0.54	0.15	6.99	0.10	0.00	0.05	0.03	0.05	750.73	1219.83
627	ASGR1	0.000371424	0.010349088	-1.33	0.37	2.70	0.45	0.10	0.06	0.23	0.23	51.32	11.45
628	KIF17	0.00037026	0.010349088	0.52	0.15	7.34	0.09	0.00	0.05	0.03	0.05	1029.93	1748.50
629	TLE2	0.000370978	0.010349088	0.74	0.21	4.72	0.17	0.01	0.07	0.09	0.09	67.02	131.97
630	BCAT2	0.000372054	0.010350197	0.73	0.21	7.68	0.19	0.00	0.10	0.03	0.10	1326.11	2600.63
631	ARPC2	0.000376624	0.010460717	-0.42	0.12	7.51	0.06	0.00	0.03	0.03	0.03	2667.60	1700.25
632	HERC2P9	0.000377785	0.01047636	-0.53	0.15	6.17	0.10	0.01	0.05	0.04	0.05	781.87	435.97
633	PPM1L	0.000382048	0.010561152	-1.25	0.35	6.12	0.54	0.00	0.30	0.04	0.30	1534.00	382.47
634	UBE2B	0.000382035	0.010561152	-0.41	0.12	7.20	0.05	0.00	0.03	0.03	0.03	1909.33	1244.51

635	AP1S3	0.000383802	0.010591524	-1.25	0.35	3.65	0.55	0.03	0.27	0.17	0.27	167.28	31.17
636	TOP1	0.000384355	0.010591524	-0.42	0.12	8.02	0.05	0.00	0.02	0.03	0.03	4279.48	2838.71
637	CCRL2	0.000385773	0.010607488	-1.09	0.31	3.91	0.41	0.03	0.20	0.15	0.20	158.25	41.72
638	CUL7	0.000386145	0.010607488	0.54	0.15	7.76	0.10	0.00	0.05	0.03	0.05	1664.24	2618.29
639	RPL10	0.000389768	0.010680303	0.54	0.15	10.43	0.09	0.00	0.04	0.05	0.05	22943.11	38029.43
640	SMCO4	0.000390014	0.010680303	0.57	0.16	5.86	0.11	0.00	0.06	0.04	0.06	245.63	396.17
641	IL1R2	0.000392814	0.010740172	-2.12	0.60	3.92	1.57	0.08	0.77	0.15	0.77	286.77	45.88
642	SETBP1	0.000395789	0.010804677	0.90	0.25	4.99	0.28	0.02	0.14	0.08	0.14	81.16	185.05
643	PFKFB3	0.000398238	0.010854629	-0.58	0.16	6.63	0.12	0.00	0.06	0.03	0.06	1230.90	695.17
644	MYL12A	0.000398962	0.010857473	-0.43	0.12	8.28	0.06	0.00	0.04	0.03	0.04	5814.97	3636.35
645	OSGEPL1	0.00040049	0.010865303	0.42	0.12	6.50	0.05	0.00	0.02	0.03	0.03	474.57	726.76
646	ZFPM2	0.000400164	0.010865303	0.85	0.24	4.79	0.25	0.02	0.12	0.09	0.12	68.47	148.41
647	NEK7	0.000401443	0.010873986	0.59	0.17	7.26	0.12	0.00	0.07	0.03	0.07	900.74	1651.66
648	PID1	0.000402051	0.010873986	0.48	0.14	7.31	0.08	0.00	0.04	0.03	0.04	1058.98	1665.09
649	KCTD10	0.000409196	0.011050176	-0.47	0.13	5.99	0.05	0.00	0.01	0.04	0.04	588.50	365.05
650	IFITM3	0.000411147	0.011051795	0.41	0.12	7.89	0.05	0.00	0.02	0.03	0.03	1926.20	2928.50
651	MEA1	0.000409962	0.011051795	0.41	0.12	7.31	0.04	0.00	0.01	0.03	0.03	1094.45	1631.59
652	SGSM1	0.000410992	0.011051795	1.12	0.32	5.96	0.44	0.01	0.23	0.04	0.23	212.77	501.16
653	ERBIN	0.000413161	0.011088924	-0.42	0.12	6.62	0.06	0.00	0.03	0.03	0.03	1096.38	691.10
654	TUBB1	0.000414533	0.01110872	-1.38	0.39	2.80	0.62	0.14	0.18	0.22	0.22	54.07	13.59
655	ATAD3B	0.000415994	0.011130845	1.28	0.36	6.11	0.58	0.01	0.31	0.04	0.31	251.09	591.80
656	NDFIP1	0.000418157	0.011171669	-0.55	0.16	8.61	0.11	0.00	0.06	0.04	0.06	8885.03	5024.34
657	NME3	0.000418953	0.011175909	0.80	0.23	6.88	0.22	0.00	0.12	0.03	0.12	570.88	1173.66
658	POLD2	0.000421071	0.011215334	0.41	0.12	6.73	0.05	0.00	0.02	0.03	0.03	610.26	915.65
659	ACAN	0.000425116	0.011258986	-2.42	0.69	4.80	2.06	0.08	1.05	0.09	1.05	1224.54	117.26
660	ASAP1	0.00042528	0.011258986	-0.46	0.13	6.08	0.07	0.00	0.03	0.04	0.04	634.64	402.79
661	MRPS14	0.000424018	0.011258986	0.42	0.12	6.59	0.04	0.00	0.01	0.03	0.03	521.76	795.39
662	TXNRD3	0.000423639	0.011258986	0.44	0.12	6.29	0.06	0.00	0.02	0.03	0.03	386.95	590.61
663	H2AFY	0.000426415	0.01127201	-0.41	0.12	7.79	0.04	0.00	0.01	0.03	0.03	3405.58	2246.53

664	FNDC10	0.000432178	0.011407154	0.63	0.18	6.49	0.14	0.00	0.08	0.03	0.08	398.12	774.21
665	C6orf203	0.000433405	0.011417337	0.41	0.12	7.06	0.06	0.00	0.03	0.03	0.03	847.87	1279.58
666	LSM2	0.000433867	0.011417337	0.43	0.12	6.36	0.06	0.00	0.03	0.03	0.03	424.41	634.10
667	ADAMTSL3	0.000435382	0.011440025	0.42	0.12	6.43	0.06	0.00	0.03	0.03	0.03	447.00	681.45
668	CHSY1	0.000437246	0.011471812	-0.64	0.18	6.44	0.14	0.01	0.07	0.03	0.07	1118.60	561.65
669	SLC7A4	0.000438734	0.011493639	1.33	0.38	3.68	0.62	0.05	0.29	0.16	0.29	19.05	55.91
670	AMER1	0.000441223	0.011541606	-0.48	0.14	5.91	0.07	0.00	0.03	0.04	0.04	543.60	341.04
671	CHCHD2	0.00044231	0.011552786	0.48	0.14	9.60	0.08	0.00	0.04	0.04	0.04	10459.54	16498.23
672	SVIP	0.000443683	0.011571403	0.48	0.14	5.86	0.07	0.00	0.03	0.04	0.04	243.08	392.30
673	IKZF2	0.000445727	0.011607457	-0.60	0.17	5.30	0.11	0.01	0.04	0.06	0.06	335.06	181.35
674	WDR5B	0.00044777	0.011643352	0.42	0.12	6.47	0.06	0.00	0.03	0.03	0.03	464.53	710.07
675	MRPS34	0.000449167	0.011662383	0.41	0.12	7.59	0.06	0.00	0.03	0.03	0.03	1424.56	2188.57
676	DHX32	0.000451271	0.01169967	-0.61	0.17	7.58	0.13	0.00	0.07	0.03	0.07	3622.98	1750.78
677	RPL23	0.000453742	0.011746363	0.48	0.14	9.74	0.07	0.00	0.04	0.05	0.05	11831.79	18923.79
678	NEDD4	0.00045831	0.011847099	-0.54	0.15	8.38	0.10	0.00	0.05	0.03	0.05	7054.49	3966.16
679	COL16A1	0.000459044	0.011848615	1.12	0.32	5.10	0.44	0.01	0.23	0.07	0.23	79.06	221.83
680	PLCB3	0.000468738	0.012081042	0.61	0.18	7.16	0.13	0.00	0.07	0.03	0.07	853.79	1480.63
681	ATF7IP2	0.000470087	0.012098008	1.11	0.32	6.16	0.43	0.00	0.24	0.04	0.24	244.18	620.86
682	TMEM61	0.000473887	0.012177928	0.88	0.25	4.18	0.27	0.02	0.13	0.13	0.13	35.29	82.15
683	ECI1	0.000478645	0.01228218	0.42	0.12	6.74	0.06	0.00	0.03	0.03	0.03	610.57	931.72
684	SSNA1	0.00048248	0.012362502	0.42	0.12	6.54	0.06	0.00	0.03	0.03	0.03	495.42	763.27
685	HMGXB3	0.000487656	0.012476884	-0.50	0.14	7.49	0.09	0.00	0.05	0.03	0.05	2810.71	1632.86
686	CEP120	0.000489832	0.012514278	-0.42	0.12	7.57	0.05	0.00	0.02	0.03	0.03	2725.77	1804.69
687	MACROD2	0.000495577	0.012624239	-0.87	0.25	4.57	0.27	0.01	0.14	0.10	0.14	246.70	83.02
688	NDUFB4	0.000495393	0.012624239	0.43	0.12	8.71	0.06	0.00	0.03	0.04	0.04	4449.82	6683.13
689	TANC2	0.000499	0.012692987	-0.71	0.20	6.42	0.18	0.02	0.08	0.03	0.08	1096.37	559.41
690	MRPL12	0.000501234	0.012731338	0.43	0.12	6.40	0.06	0.00	0.03	0.03	0.03	441.99	660.28
691	UQCRB	0.00050225	0.012738682	0.48	0.14	9.00	0.08	0.00	0.05	0.04	0.05	5705.76	9044.30
692	BHLHE41	0.000504035	0.012765494	0.90	0.26	4.99	0.29	0.01	0.15	0.08	0.15	79.83	185.89

693	PECR	0.000506407	0.012793897	-0.77	0.22	5.88	0.21	0.00	0.12	0.04	0.12	732.80	318.02
694	TRAF4	0.000506617	0.012793897	-0.84	0.24	5.12	0.25	0.01	0.13	0.07	0.13	327.94	149.96
695	MCM7	0.000510794	0.012855768	0.42	0.12	6.40	0.06	0.00	0.03	0.03	0.03	436.62	665.17
696	NTRK2	0.000511267	0.012855768	1.41	0.40	5.79	0.70	0.01	0.39	0.04	0.39	150.90	477.09
697	PATZ1	0.000509901	0.012855768	0.41	0.12	6.84	0.04	0.00	0.01	0.03	0.03	680.58	1016.22
698	PBXIP1	0.000512424	0.01286639	0.43	0.12	8.63	0.05	0.00	0.02	0.04	0.04	3989.53	6151.24
699	KIAA1210	0.000515026	0.012876376	2.28	0.66	6.49	1.86	0.00	1.04	0.03	1.04	176.19	1407.78
700	MAMDC2	0.000514385	0.012876376	-1.26	0.36	6.13	0.56	0.00	0.31	0.04	0.31	1509.46	402.20
701	PKD1	0.000514691	0.012876376	0.53	0.15	5.66	0.09	0.01	0.03	0.05	0.05	192.03	324.85
702	DOCK8	0.000517226	0.012912967	-0.56	0.16	7.68	0.11	0.00	0.06	0.03	0.06	3545.57	1972.54
703	DCHS2	0.00051869	0.012931095	-1.82	0.52	2.77	1.17	0.06	0.60	0.23	0.60	195.22	11.45
704	HSPB2	0.000523153	0.013023832	1.05	0.30	3.85	0.39	0.07	0.15	0.15	0.15	22.18	62.91
705	CARHSP1	0.000523984	0.013026016	0.66	0.19	7.08	0.15	0.00	0.09	0.03	0.09	779.57	1385.17
706	RPS27	0.000525432	0.013043523	0.51	0.15	10.24	0.09	0.00	0.05	0.05	0.05	19022.06	31609.13
707	TIMM17B	0.00052821	0.01309393	0.40	0.12	6.94	0.05	0.00	0.02	0.03	0.03	754.75	1131.69
708	THBS3	0.000531161	0.013148496	0.58	0.17	5.71	0.12	0.01	0.06	0.05	0.06	195.84	347.90
709	RPS27A	0.0005348	0.013219904	0.45	0.13	9.32	0.07	0.00	0.04	0.04	0.04	7898.46	12391.73
710	IFITM2	0.000536966	0.013248859	0.56	0.16	6.86	0.11	0.00	0.06	0.03	0.06	627.68	1094.14
711	PPP2R5E	0.000537484	0.013248859	-0.42	0.12	7.01	0.06	0.00	0.03	0.03	0.03	1634.89	1024.68
712	CFAP221	0.000538551	0.013256529	0.85	0.25	5.47	0.26	0.01	0.14	0.05	0.14	129.34	299.73
713	IMP4	0.000542128	0.013307184	0.47	0.14	6.85	0.08	0.00	0.04	0.03	0.04	686.81	1047.82
714	OSR2	0.000541927	0.013307184	1.21	0.35	4.69	0.53	0.01	0.28	0.10	0.28	50.79	150.87
715	MAPK1IP1L	0.000546905	0.013386958	-0.43	0.13	6.19	0.05	0.00	0.02	0.04	0.04	710.19	450.90
716	PRKACA	0.000546791	0.013386958	-0.57	0.17	7.71	0.12	0.00	0.06	0.03	0.06	3677.52	2028.55
717	AKIP1	0.000549743	0.013428743	0.42	0.12	6.59	0.05	0.00	0.02	0.03	0.03	527.97	801.07
718	KIF21B	0.000550145	0.013428743	-0.79	0.23	4.48	0.19	0.02	0.07	0.11	0.11	174.13	77.90
719	ABHD4	0.000551935	0.013453702	-0.65	0.19	5.79	0.15	0.00	0.08	0.04	0.08	589.34	294.04
720	TBX18	0.000558779	0.013601607	1.52	0.44	3.48	0.83	0.03	0.43	0.18	0.43	9.90	61.77
721	FAM43B	0.000561209	0.013641827	0.57	0.17	6.62	0.12	0.00	0.06	0.03	0.06	472.35	867.25

722	TDG	0.00057151	0.013872276	-0.49	0.14	5.72	0.06	0.00	0.02	0.04	0.04	455.71	277.48
723	TIAL1	0.000572273	0.013872276	-0.41	0.12	6.64	0.05	0.00	0.02	0.03	0.03	1079.66	709.55
724	CLMN	0.000573707	0.013887835	-1.09	0.32	3.10	0.41	0.04	0.18	0.20	0.20	57.47	19.21
725	PAPSS2	0.000580148	0.01402437	-0.69	0.20	9.28	0.17	0.00	0.10	0.04	0.10	19012.34	9817.90
726	RALGDS	0.000582134	0.014052999	0.41	0.12	7.84	0.06	0.00	0.03	0.03	0.03	1890.72	2797.21
727	GFRA2	0.000583034	0.014055374	-1.86	0.54	3.11	1.24	0.06	0.64	0.20	0.64	120.74	19.97
728	ADAM22	0.000585903	0.014085776	-0.71	0.21	7.33	0.18	0.00	0.10	0.03	0.10	2758.98	1397.58
729	CDYL2	0.00058568	0.014085776	-1.11	0.32	5.71	0.44	0.02	0.23	0.05	0.23	932.50	259.02
730	HTN3	0.000587053	0.014094109	-1.47	0.43	3.50	0.78	0.05	0.39	0.17	0.39	240.20	25.00
731	CDC42SE2	0.000591246	0.0141174	-0.53	0.16	5.40	0.08	0.00	0.03	0.06	0.06	357.59	199.67
732	MRPS18B	0.000590658	0.0141174	0.41	0.12	8.08	0.05	0.00	0.02	0.03	0.03	2350.49	3522.87
733	PEX12	0.00059114	0.0141174	0.41	0.12	6.51	0.05	0.00	0.02	0.03	0.03	485.54	740.08
734	PITPNB	0.000589882	0.0141174	-0.46	0.13	5.89	0.06	0.00	0.03	0.04	0.04	527.48	333.10
735	GCH1	0.000597102	0.014237835	-1.39	0.41	4.80	0.70	0.01	0.38	0.09	0.38	523.84	106.17
736	NLE1	0.000603302	0.014366118	0.53	0.15	5.51	0.07	0.00	0.02	0.05	0.05	162.59	276.41
737	RCC1L	0.000609551	0.01447626	0.40	0.12	7.47	0.05	0.00	0.02	0.03	0.03	1297.78	1918.90
738	ZDHHC20	0.000609579	0.01447626	-0.50	0.15	7.14	0.09	0.00	0.05	0.03	0.05	2012.99	1148.76
739	PFDN5	0.000610921	0.0144885	0.44	0.13	9.04	0.05	0.00	0.02	0.04	0.04	5988.33	9279.89
740	MARCH3	0.0006141	0.014530336	-0.59	0.17	5.55	0.13	0.01	0.07	0.05	0.07	416.08	236.84
741	PAGR1	0.000614343	0.014530336	0.40	0.12	6.90	0.04	0.00	0.02	0.03	0.03	724.15	1086.37
742	SMYD2	0.000620046	0.014645465	-0.83	0.24	6.59	0.25	0.00	0.14	0.03	0.14	1735.35	630.95
743	SEL1L	0.000622492	0.014683446	-0.41	0.12	6.61	0.05	0.00	0.02	0.03	0.03	1045.86	686.34
744	MRPL48	0.00062335	0.014683915	0.41	0.12	6.70	0.05	0.00	0.02	0.03	0.03	589.05	886.12
745	FANK1	0.000625323	0.014710607	-0.91	0.27	4.97	0.30	0.01	0.16	0.08	0.16	309.22	128.67
746	RPS14	0.000627386	0.01473936	0.48	0.14	9.62	0.08	0.00	0.05	0.04	0.05	10411.90	16907.24
747	HIST1H1A	0.000630361	0.014769663	1.82	0.53	2.59	1.19	0.08	0.60	0.24	0.60	5.12	21.70
748	PARP11	0.000629944	0.014769663	-0.58	0.17	7.11	0.12	0.01	0.06	0.03	0.06	2021.23	1114.03
749	EPDR1	0.000635193	0.014856746	0.48	0.14	6.97	0.08	0.00	0.05	0.03	0.05	757.78	1189.40
750	GLMP	0.000636621	0.014856746	0.50	0.15	7.26	0.09	0.00	0.05	0.03	0.05	966.94	1602.38

751	LAMTOR2	0.000635854	0.014856746	0.40	0.12	6.70	0.05	0.00	0.02	0.03	0.03	590.26	886.70
752	SAMD1	0.000637551	0.014858673	0.64	0.19	4.98	0.13	0.01	0.06	0.08	0.08	87.80	170.64
753	CALCRL	0.000641952	0.01492877	-0.54	0.16	5.43	0.11	0.00	0.06	0.06	0.06	370.11	209.28
754	LAMB4	0.000642262	0.01492877	-1.61	0.47	3.22	0.93	0.30	0.23	0.19	0.23	118.38	21.74
755	SDCBP	0.000643237	0.014931613	-0.42	0.12	8.61	0.06	0.00	0.03	0.04	0.04	7894.17	5103.27
756	FOXP1	0.000659237	0.015251381	-0.54	0.16	5.42	0.08	0.00	0.03	0.06	0.06	349.48	205.92
757	PIK3R2	0.000658809	0.015251381	0.52	0.15	5.47	0.07	0.00	0.02	0.05	0.05	157.02	265.93
758	PWWP2B	0.000659675	0.015251381	0.55	0.16	6.63	0.11	0.00	0.06	0.03	0.06	491.14	875.81
759	SPECC1	0.000660493	0.015251381	0.58	0.17	6.55	0.12	0.00	0.07	0.03	0.07	448.16	810.08
760	PSD3	0.0006731	0.015522042	-0.51	0.15	7.13	0.09	0.00	0.05	0.03	0.05	1959.07	1142.81
761	SF3B5	0.000676356	0.015576635	0.40	0.12	7.76	0.05	0.00	0.03	0.03	0.03	1743.69	2554.81
762	SPPL2A	0.000679129	0.015619974	-0.39	0.12	7.21	0.05	0.00	0.02	0.03	0.03	1879.03	1266.96
763	HEXIM2	0.000688873	0.01580261	0.56	0.17	5.32	0.08	0.01	0.03	0.06	0.06	130.67	231.31
764	MMP19	0.000688511	0.01580261	-0.76	0.22	4.65	0.19	0.02	0.08	0.10	0.10	200.78	93.11
765	PRKCQ	0.000689951	0.015806635	-3.09	0.91	4.34	3.49	0.05	1.95	0.12	1.95	1286.61	92.60
766	FNDC4	0.000695939	0.015923022	-0.41	0.12	8.32	0.06	0.00	0.03	0.03	0.03	5748.57	3839.81
767	RPL26	0.000698931	0.015970609	0.48	0.14	9.61	0.08	0.00	0.05	0.04	0.05	10381.13	16656.49
768	ENO1	0.000700293	0.015980897	0.42	0.12	8.75	0.06	0.00	0.03	0.04	0.04	4586.27	6915.16
769	MPV17	0.000702673	0.016014368	0.54	0.16	7.44	0.11	0.00	0.06	0.03	0.06	1174.68	1929.20
770	RPS7	0.000709165	0.016141326	0.45	0.13	9.51	0.06	0.00	0.03	0.04	0.04	9408.85	14920.92
771	DNAJA1	0.000712515	0.016196547	-0.39	0.11	7.38	0.04	0.00	0.01	0.03	0.03	2201.67	1499.17
772	EFNA5	0.00071362	0.016200662	-1.96	0.58	3.39	1.41	0.05	0.75	0.18	0.75	221.07	26.99
773	CD55	0.000718503	0.016290399	0.79	0.23	9.57	0.23	0.00	0.13	0.04	0.13	7866.10	17887.99
774	HERC2	0.000724132	0.01635456	-0.45	0.13	6.00	0.06	0.00	0.02	0.04	0.04	596.39	372.58
775	NGFR	0.000722728	0.01635456	0.74	0.22	6.08	0.20	0.00	0.11	0.04	0.11	274.01	524.10
776	RPL12	0.000723213	0.01635456	0.46	0.14	9.64	0.08	0.00	0.04	0.04	0.04	11156.12	17151.82
777	PLAGL2	0.000727503	0.016375085	-0.53	0.16	5.56	0.10	0.00	0.05	0.05	0.05	406.03	239.71
778	RPL22	0.00072679	0.016375085	0.41	0.12	8.35	0.06	0.00	0.03	0.03	0.03	3084.89	4657.88
779	USP9X	0.000727844	0.016375085	-0.39	0.11	7.02	0.05	0.00	0.02	0.03	0.03	1527.89	1048.30

780	FBXO34	0.000729764	0.016397232	-0.48	0.14	6.45	0.08	0.00	0.05	0.03	0.05	995.28	574.97
781	VPS11	0.000731326	0.016411293	0.39	0.12	7.52	0.05	0.00	0.02	0.03	0.03	1374.48	2017.03
782	SSBP4	0.000736734	0.016511511	0.56	0.17	6.08	0.12	0.01	0.06	0.04	0.06	293.04	498.89
783	ABCC5	0.000743219	0.016635582	-0.45	0.13	6.61	0.08	0.00	0.04	0.03	0.04	1113.15	688.25
784	COQ7	0.000750049	0.016703118	0.44	0.13	6.06	0.05	0.00	0.02	0.04	0.04	303.54	471.38
785	CYTIP	0.000749354	0.016703118	-1.39	0.41	2.96	0.71	0.04	0.37	0.21	0.37	87.07	16.01
786	DCAF5	0.00074842	0.016703118	-0.40	0.12	7.56	0.05	0.00	0.02	0.03	0.03	2727.22	1771.02
787	TBL1X	0.000748977	0.016703118	-0.51	0.15	5.64	0.10	0.01	0.05	0.05	0.05	448.72	258.13
788	FAM127B	0.000754086	0.016771714	0.46	0.14	5.92	0.05	0.00	0.01	0.04	0.04	260.70	409.02
789	MOB4	0.000757684	0.016830381	-1.39	0.41	8.28	0.71	0.00	0.41	0.03	0.41	16571.53	3448.47
790	ACAP2	0.000760694	0.016849288	-0.42	0.12	6.19	0.05	0.00	0.02	0.04	0.04	692.01	453.95
791	DYNLL2	0.000761419	0.016849288	-0.43	0.13	9.13	0.06	0.00	0.03	0.04	0.04	13196.86	8547.94
792	RTKN	0.000759865	0.016849288	0.64	0.19	4.90	0.11	0.00	0.04	0.08	0.08	83.51	153.44
793	TCF7	0.000768622	0.016965839	-0.80	0.24	4.73	0.24	0.01	0.13	0.09	0.13	265.81	97.57
794	ZMAT3	0.000768092	0.016965839	-0.50	0.15	7.69	0.09	0.00	0.05	0.03	0.05	3476.91	2004.91
795	HAUS4	0.000772243	0.017024325	0.42	0.12	6.35	0.06	0.00	0.03	0.03	0.03	409.20	631.25
796	SEPT6	0.000780262	0.017179498	0.47	0.14	8.80	0.08	0.00	0.05	0.04	0.05	4608.96	7472.42
797	RPL31	0.000783913	0.017238217	0.50	0.15	10.26	0.07	0.00	0.03	0.05	0.05	19284.22	31845.74
798	CCNK	0.000788495	0.017295582	-0.65	0.19	5.38	0.15	0.01	0.08	0.06	0.08	377.52	196.39
799	CELF2	0.000787885	0.017295582	-0.83	0.25	5.81	0.26	0.01	0.14	0.04	0.14	653.56	306.88
800	SRI	0.000793222	0.017377503	0.39	0.12	7.58	0.05	0.00	0.03	0.03	0.03	1499.94	2119.73
801	CTBP2	0.000798493	0.017471151	-0.42	0.13	6.45	0.06	0.00	0.02	0.03	0.03	893.21	590.08
802	RPL30	0.000802431	0.017535412	0.45	0.14	9.57	0.07	0.00	0.03	0.04	0.04	10291.48	15871.79
803	AADAT	0.000809614	0.017670355	1.06	0.32	3.89	0.42	0.03	0.21	0.15	0.21	28.47	62.87
804	ACTR3	0.000811183	0.017682573	0.39	0.12	7.94	0.04	0.00	0.02	0.03	0.03	2073.08	3041.31
805	RPL35	0.000815434	0.017753163	0.44	0.13	9.30	0.05	0.00	0.02	0.04	0.04	7745.09	12112.49
806	BIRC3	0.00082017	0.017767994	-0.98	0.29	3.78	0.36	0.02	0.19	0.16	0.19	117.20	37.84
807	GIPC2	0.000817377	0.017767994	0.44	0.13	7.83	0.07	0.00	0.04	0.03	0.04	1803.41	2800.78
808	HELB	0.000819214	0.017767994	-0.74	0.22	4.60	0.20	0.01	0.10	0.10	0.10	185.92	89.64

809	NXN	0.000818569	0.017767994	1.74	0.52	4.86	1.13	0.01	0.64	0.08	0.64	49.09	224.03
810	USP35	0.000822573	0.017798041	0.51	0.15	7.51	0.09	0.00	0.05	0.03	0.05	1233.10	2077.84
811	HSPA2	0.000829813	0.017910481	1.12	0.33	6.20	0.46	0.00	0.26	0.04	0.26	219.39	690.41
812	IL1R1	0.000828986	0.017910481	-1.14	0.34	7.61	0.48	0.00	0.28	0.03	0.28	6188.36	1753.06
813	PNMAL1	0.000835354	0.018007897	0.67	0.20	8.09	0.17	0.00	0.10	0.03	0.10	2047.05	3849.72
814	PCBP4	0.000838345	0.018050167	0.45	0.14	6.00	0.06	0.01	0.02	0.04	0.04	284.62	447.67
815	SLC31A1	0.000854239	0.018347306	-0.61	0.18	7.59	0.14	0.00	0.08	0.03	0.08	3323.06	1811.72
816	THOC6	0.000853371	0.018347306	0.50	0.15	5.53	0.06	0.00	0.02	0.05	0.05	168.28	281.49
817	LLGL1	0.000857388	0.018350871	0.46	0.14	7.61	0.08	0.00	0.04	0.03	0.04	1475.08	2230.41
818	POGLUT1	0.000856983	0.018350871	-0.44	0.13	6.35	0.07	0.00	0.04	0.03	0.04	835.86	529.38
819	SYTL2	0.000857547	0.018350871	-1.19	0.36	7.55	0.53	0.00	0.30	0.03	0.30	5922.61	1682.94
820	RAB32	0.00086405	0.018467486	0.48	0.14	6.11	0.09	0.00	0.05	0.04	0.05	313.59	508.95
821	B4GAT1	0.00086545	0.018474878	0.39	0.12	7.44	0.05	0.00	0.03	0.03	0.03	1276.82	1856.13
822	CENPB	0.000867984	0.018506161	0.41	0.12	7.93	0.06	0.00	0.04	0.03	0.04	2038.01	3073.18
823	TANC1	0.000869027	0.018506161	-0.48	0.14	7.36	0.09	0.00	0.05	0.03	0.05	2327.48	1458.08
824	CDC20P1	0.000871648	0.018539443	1.30	0.39	2.63	0.63	0.10	0.26	0.24	0.26	5.17	20.98
825	BNC2	0.000883358	0.01871822	1.08	0.32	4.51	0.44	0.02	0.23	0.11	0.23	49.41	117.96
826	IFI27	0.000885068	0.01871822	-1.12	0.34	7.60	0.47	0.00	0.27	0.03	0.27	6351.78	1706.59
827	NDRG4	0.000885393	0.01871822	1.38	0.41	7.19	0.71	0.00	0.41	0.03	0.41	664.76	1936.35
828	PDIA5	0.000881629	0.01871822	0.51	0.15	5.84	0.10	0.00	0.05	0.04	0.05	246.16	386.10
829	RAD54L2	0.000884731	0.01871822	-0.39	0.12	7.00	0.05	0.00	0.03	0.03	0.03	1520.08	1021.31
830	COQ9	0.000889243	0.018776947	0.42	0.13	7.38	0.07	0.00	0.04	0.03	0.04	1156.65	1783.62
831	ADM	0.000894382	0.018862733	0.86	0.26	4.86	0.28	0.02	0.14	0.08	0.14	66.59	167.13
832	MYO9A	0.000896714	0.018889196	-0.50	0.15	5.68	0.09	0.01	0.05	0.05	0.05	477.38	267.87
833	ARHGAP18	0.000899458	0.018901554	-0.47	0.14	6.54	0.08	0.00	0.05	0.03	0.05	1031.97	643.52
834	DCP2	0.000899298	0.018901554	-0.44	0.13	6.21	0.05	0.01	0.01	0.04	0.04	712.65	457.71
835	C8orf33	0.000902147	0.018935373	0.53	0.16	7.56	0.11	0.00	0.06	0.03	0.06	1387.98	2143.59
836	PGLS	0.000913556	0.019151886	0.47	0.14	6.45	0.08	0.00	0.05	0.03	0.05	451.71	705.39
837	FAM50A	0.000925447	0.019319511	0.39	0.12	6.69	0.03	0.00	0.01	0.03	0.03	592.33	874.54

838	RPS7P1	0.000924177	0.019319511	0.77	0.23	4.48	0.16	0.02	0.04	0.11	0.11	49.34	104.94
839	UQCC3	0.000925936	0.019319511	0.54	0.16	6.29	0.11	0.00	0.06	0.03	0.06	354.63	614.58
840	ZNF385A	0.000925961	0.019319511	-1.50	0.45	4.72	0.84	0.03	0.47	0.09	0.47	579.56	95.90
841	GAPDHP23	0.000928908	0.019350298	1.56	0.47	1.69	0.87	0.22	0.27	0.32	0.32	1.99	8.15
842	NUDT16L1	0.000929645	0.019350298	0.40	0.12	7.04	0.06	0.00	0.03	0.03	0.03	856.36	1257.46
843	LY6D	0.000932475	0.019356233	1.90	0.58	6.38	1.36	0.01	0.79	0.03	0.79	307.41	1065.58
844	PPM1B	0.000932315	0.019356233	-0.39	0.12	7.32	0.06	0.00	0.03	0.03	0.03	2177.39	1405.79
845	TNFSF15	0.000933243	0.019356233	0.96	0.29	5.16	0.35	0.01	0.20	0.07	0.20	94.19	224.71
846	C1QL3	0.00093452	0.019359815	1.02	0.31	4.12	0.39	0.02	0.21	0.14	0.21	31.13	81.17
847	ESR2	0.000939448	0.01943892	0.92	0.28	6.27	0.32	0.00	0.18	0.03	0.18	324.11	660.27
848	RPL37	0.000943871	0.0195074	0.45	0.14	9.58	0.07	0.00	0.04	0.04	0.04	10220.97	16071.43
849	DCAKD	0.000949233	0.019595129	0.38	0.12	7.03	0.04	0.00	0.02	0.03	0.03	847.22	1221.50
850	ATG2B	0.000957483	0.019632768	-0.55	0.17	5.26	0.10	0.00	0.05	0.06	0.06	319.03	174.84
851	IGF1	0.000957736	0.019632768	1.42	0.43	4.80	0.76	0.02	0.42	0.09	0.42	55.80	183.21
852	MTOR	0.000955823	0.019632768	-0.59	0.18	6.43	0.13	0.00	0.07	0.03	0.07	1122.28	554.36
853	PLN	0.000952414	0.019632768	1.28	0.39	6.18	0.61	0.00	0.36	0.04	0.36	177.23	774.52
854	TAX1BP3	0.000954589	0.019632768	0.60	0.18	7.59	0.14	0.00	0.08	0.03	0.08	1268.42	2308.49
855	TPRXL	0.000957778	0.019632768	-0.76	0.23	4.59	0.19	0.02	0.07	0.10	0.10	181.06	88.67
856	CORO2A	0.000960476	0.019665075	-0.90	0.27	3.94	0.22	0.03	0.06	0.15	0.15	108.14	43.95
857	SFRP4	0.000965919	0.019753437	1.66	0.50	4.74	1.04	0.01	0.59	0.09	0.59	41.21	211.97
858	GLS	0.00096777	0.019768218	0.39	0.12	7.74	0.06	0.00	0.03	0.03	0.03	1728.10	2505.12
859	RGL2	0.000973025	0.019852424	0.39	0.12	6.69	0.06	0.00	0.03	0.03	0.03	608.32	880.00
860	BICD1	0.000978759	0.019946207	-0.70	0.21	4.97	0.19	0.01	0.10	0.08	0.10	272.70	130.11
861	PPARGC1A	0.000980845	0.019965498	1.63	0.49	4.72	1.00	0.01	0.57	0.09	0.57	56.36	172.52
862	MRAP2	0.000986047	0.020048102	1.65	0.50	4.51	1.02	0.02	0.58	0.11	0.58	57.83	141.77
863	RAB34	0.000987327	0.020050863	1.43	0.43	7.56	0.77	0.00	0.45	0.03	0.45	1115.45	2642.82
864	RPS29	0.000994716	0.020177546	0.45	0.14	9.69	0.08	0.00	0.04	0.04	0.04	11638.16	17999.06
865	ABCA3	0.000997727	0.020215221	0.89	0.27	6.31	0.30	0.00	0.17	0.03	0.17	330.17	682.49
866	MEI4	0.001003622	0.020311174	-1.57	0.48	4.71	0.93	0.02	0.53	0.09	0.53	657.99	93.99

867	SPOCD1	0.001009051	0.02039749	-1.34	0.41	2.82	0.47	0.18	-0.01	0.22	0.22	51.82	13.48
868	AAK1	0.001013161	0.020430629	-0.38	0.12	6.73	0.05	0.00	0.03	0.03	0.03	1138.83	784.66
869	COX8A	0.001012185	0.020430629	0.40	0.12	8.35	0.05	0.00	0.02	0.03	0.03	3083.60	4602.36
870	TEF	0.001014187	0.020430629	-0.73	0.22	7.21	0.20	0.01	0.11	0.03	0.11	2664.50	1226.42
871	TP53	0.001015513	0.020433841	0.38	0.12	7.09	0.04	0.00	0.01	0.03	0.03	890.14	1302.69
872	YTHDC2	0.001019582	0.020492185	-0.39	0.12	6.96	0.04	0.00	0.01	0.03	0.03	1454.01	978.57
873	ITGA3	0.001024699	0.020565892	0.47	0.14	6.77	0.08	0.00	0.05	0.03	0.05	597.98	976.23
874	PAWR	0.001025596	0.020565892	0.57	0.17	5.21	0.09	0.01	0.02	0.07	0.07	118.58	208.24
875	BAD	0.001027858	0.020587711	0.65	0.20	6.21	0.16	0.00	0.09	0.04	0.09	331.77	578.29
876	LPCAT1	0.001031816	0.020643392	0.56	0.17	5.81	0.12	0.00	0.06	0.04	0.06	219.11	386.18
877	FASTK	0.001033138	0.020646262	0.42	0.13	7.03	0.07	0.00	0.04	0.03	0.04	829.51	1253.15
878	DENND2A	0.001043462	0.020740414	0.68	0.21	4.81	0.13	0.02	0.04	0.09	0.09	72.06	142.46
879	FUNDC2	0.001043766	0.020740414	0.38	0.12	7.60	0.05	0.00	0.03	0.03	0.03	1524.28	2163.57
880	KCNJ8	0.001042218	0.020740414	1.82	0.55	6.50	1.25	0.00	0.73	0.03	0.73	312.48	1158.56
881	KCTD14	0.001040507	0.020740414	0.51	0.16	6.96	0.10	0.00	0.06	0.03	0.06	720.62	1197.32
882	TARS	0.001043024	0.020740414	-0.78	0.24	7.63	0.23	0.00	0.13	0.03	0.13	4389.80	1844.98
883	CH507-9B2.5	0.001060406	0.021034857	0.72	0.22	4.86	0.20	0.00	0.11	0.08	0.11	79.03	153.65
884	ZNF599	0.001060984	0.021034857	0.50	0.15	5.61	0.05	0.01	0.00	0.05	0.05	183.46	301.80
885	NMNAT3	0.001066071	0.021082954	0.62	0.19	4.94	0.13	0.01	0.06	0.08	0.08	93.59	159.78
886	NONO	0.001067424	0.021082954	0.39	0.12	8.31	0.04	0.00	0.01	0.03	0.03	2997.54	4396.57
887	RPLP2	0.001064956	0.021082954	0.43	0.13	9.30	0.06	0.00	0.03	0.04	0.04	8021.74	12029.80
888	SERINC2	0.001068222	0.021082954	0.93	0.29	5.43	0.33	0.01	0.19	0.06	0.19	113.53	304.95
889	ATAD2	0.001074395	0.021138753	-0.43	0.13	6.40	0.07	0.01	0.04	0.03	0.04	860.38	565.12
890	HECTD4	0.001074668	0.021138753	-0.49	0.15	5.62	0.07	0.01	0.02	0.05	0.05	412.36	252.35
891	RNF20	0.00107382	0.021138753	-0.55	0.17	6.18	0.12	0.00	0.07	0.04	0.07	824.28	436.16
892	LAMTOR4	0.001083728	0.021293074	0.41	0.13	7.47	0.06	0.00	0.04	0.03	0.04	1312.51	1925.97
893	TSTD1	0.001093663	0.021464202	0.90	0.28	6.87	0.31	0.00	0.18	0.03	0.18	548.39	1211.48
894	ARHGAP35	0.001099252	0.021549774	-0.38	0.12	7.68	0.05	0.00	0.03	0.03	0.03	3027.56	2008.40
895	QTRT1	0.001100893	0.021557832	0.42	0.13	6.06	0.06	0.00	0.03	0.04	0.04	304.39	471.96

896	MRPS2	0.001105819	0.021630118	0.45	0.14	6.74	0.08	0.00	0.05	0.03	0.05	595.03	943.79
897	SCYL2	0.001107554	0.021639894	-0.42	0.13	6.03	0.06	0.00	0.03	0.04	0.04	599.92	386.88
898	SCRN2	0.001120254	0.021863667	0.51	0.16	6.33	0.10	0.00	0.06	0.03	0.06	413.78	627.16
899	BCAM	0.001125493	0.021941474	0.46	0.14	5.66	0.07	0.00	0.04	0.05	0.05	198.29	320.27
900	HECA	0.001132298	0.02204961	-0.38	0.12	7.70	0.05	0.00	0.02	0.03	0.03	3066.43	2069.12
901	RFTN1	0.001141081	0.022195992	0.65	0.20	5.21	0.16	0.00	0.09	0.07	0.09	113.16	216.55
902	ENC1	0.001148456	0.022314671	-1.69	0.52	5.52	1.10	0.01	0.64	0.05	0.64	2077.71	187.52
903	KRT18P59	0.001155384	0.022399617	1.32	0.41	3.05	0.67	0.06	0.33	0.20	0.33	7.15	32.58
904	NME2	0.001155026	0.022399617	0.44	0.13	8.55	0.07	0.00	0.04	0.04	0.04	3677.00	5781.67
905	HS1BP3	0.001166376	0.022587739	0.39	0.12	7.09	0.06	0.00	0.03	0.03	0.03	882.59	1317.25
906	SCMH1	0.001170976	0.022651797	0.38	0.12	7.24	0.04	0.00	0.01	0.03	0.03	1041.20	1512.18
907	SDPR	0.001175639	0.022716928	0.56	0.17	7.36	0.12	0.00	0.07	0.03	0.07	1018.94	1820.40
908	C2orf40	0.001184098	0.022836301	1.87	0.58	5.46	1.34	0.01	0.79	0.05	0.79	84.03	449.86
909	CPPED1	0.001184423	0.022836301	-0.41	0.13	6.50	0.06	0.00	0.04	0.03	0.04	958.15	618.01
910	SETDB2	0.001189025	0.022896359	0.50	0.15	7.59	0.09	0.00	0.05	0.03	0.05	1335.62	2257.54
911	TAF10	0.001190151	0.022896359	0.39	0.12	7.80	0.06	0.00	0.03	0.03	0.03	1808.99	2667.56
912	FTSJ1	0.001192398	0.022914442	0.39	0.12	6.34	0.03	0.00	0.00	0.03	0.03	412.39	611.82
913	MRC2	0.001213638	0.023271572	1.12	0.34	5.96	0.48	0.01	0.28	0.04	0.28	192.96	533.58
914	PQBP1	0.001212651	0.023271572	0.38	0.12	6.97	0.03	0.00	0.00	0.03	0.03	790.14	1149.23
915	GCLC	0.001219724	0.023362708	-0.46	0.14	6.90	0.08	0.00	0.05	0.03	0.05	1492.81	916.94
916	AC093838.4	0.001225959	0.023405404	0.62	0.19	5.47	0.15	0.00	0.08	0.05	0.08	148.11	278.80
917	CLEC11A	0.001224401	0.023405404	0.76	0.23	4.97	0.22	0.01	0.12	0.08	0.12	82.83	176.06
918	ZFPL1	0.001225037	0.023405404	0.52	0.16	5.95	0.10	0.00	0.06	0.04	0.06	262.69	436.59
919	FAM155A	0.001229095	0.023421702	-1.92	0.59	4.20	1.42	0.03	0.82	0.13	0.82	790.30	50.91
920	ZNF775	0.001229486	0.023421702	0.60	0.19	5.02	0.10	0.01	0.04	0.08	0.08	92.98	173.41
921	CHDH	0.001236071	0.023506888	0.83	0.26	3.79	0.25	0.00	0.14	0.16	0.16	22.42	55.91
922	MAP3K9	0.00123664	0.023506888	-0.67	0.21	5.20	0.17	0.01	0.10	0.07	0.10	354.67	160.26
923	TSKU	0.001238887	0.02352409	-1.40	0.43	5.90	0.75	0.01	0.44	0.04	0.44	2286.09	298.53
924	BCL11B	0.001241243	0.023543325	-1.91	0.59	4.11	1.41	0.04	0.80	0.14	0.80	678.47	45.91

925	ATRAID	0.001243859	0.023567434	0.38	0.12	7.99	0.04	0.00	0.02	0.03	0.03	2183.63	3190.65
926	NPAS2	0.001247626	0.023613267	0.86	0.27	5.37	0.28	0.01	0.16	0.06	0.16	129.15	262.53
927	SRGN	0.001262619	0.023871265	-0.78	0.24	7.39	0.23	0.00	0.14	0.03	0.14	3069.67	1485.47
928	HEXIM1	0.001268866	0.023937716	-0.59	0.18	7.69	0.14	0.00	0.08	0.03	0.08	3660.20	2022.13
929	HSPB2-C11orf52	0.00126798	0.023937716	2.51	0.78	2.76	2.26	1.23	0.05	0.23	0.23	12.79	30.34
930	MAL2	0.001273019	0.023990246	-2.29	0.71	2.16	2.03	0.11	1.11	0.27	1.11	75.36	10.10
931	CEACAM22P	0.001274889	0.023999689	1.43	0.44	2.91	0.79	0.10	0.37	0.22	0.37	6.10	31.55
932	NT5DC3	0.001280843	0.024085902	0.62	0.19	4.85	0.12	0.00	0.05	0.08	0.08	81.36	146.29
933	RIBC1	0.001285823	0.024127774	0.68	0.21	4.69	0.13	0.01	0.04	0.09	0.09	66.08	126.25
934	TDRD6	0.001284524	0.024127774	-0.87	0.27	5.16	0.29	0.10	0.08	0.07	0.08	383.26	156.16
935	CREB3L4	0.001291327	0.024205126	0.50	0.16	5.46	0.09	0.00	0.04	0.05	0.05	164.78	263.73
936	PPP1R14A	0.001296744	0.0242807	1.18	0.37	4.84	0.54	0.01	0.31	0.09	0.31	56.53	181.07
937	ACVR2B	0.001298584	0.02428921	1.05	0.33	6.86	0.43	0.00	0.25	0.03	0.25	427.60	1318.18
938	FAM173A	0.001300772	0.024304183	0.56	0.17	5.89	0.12	0.01	0.07	0.04	0.07	237.69	417.93
939	CD200	0.001303056	0.024320944	2.00	0.62	5.20	1.54	0.01	0.91	0.07	0.91	43.78	369.74
940	P4HTM	0.001306868	0.02434541	0.38	0.12	6.79	0.06	0.00	0.03	0.03	0.03	654.89	971.13
941	THNSL1	0.001307145	0.02434541	0.65	0.20	6.92	0.16	0.00	0.10	0.03	0.10	627.50	1195.85
942	KRT8P47	0.00130955	0.02436431	-0.93	0.29	5.07	0.34	0.01	0.19	0.07	0.19	355.62	143.79
943	CD248	0.00132606	0.024645311	1.04	0.32	4.71	0.42	0.01	0.24	0.09	0.24	50.52	151.82
944	EEF1A1P13	0.001332431	0.024737485	0.58	0.18	5.08	0.10	0.01	0.04	0.07	0.07	102.94	183.03
945	AP5M1	0.001338872	0.024830756	-0.43	0.13	6.52	0.07	0.00	0.04	0.03	0.04	1033.56	622.19
946	CRACR2A	0.001350048	0.025011563	-0.92	0.29	3.88	0.21	0.05	0.02	0.15	0.15	102.29	41.29
947	RPS15A	0.001351682	0.025015391	0.43	0.13	9.43	0.07	0.00	0.04	0.04	0.04	9011.70	13754.84
948	C6orf48	0.001368238	0.025295088	0.41	0.13	7.77	0.06	0.00	0.04	0.03	0.04	1714.89	2605.28
949	REL	0.001378415	0.025456378	-0.58	0.18	7.13	0.13	0.00	0.08	0.03	0.08	2094.01	1147.38
950	EDEM2	0.00138837	0.025613239	0.51	0.16	7.53	0.10	0.00	0.06	0.03	0.06	1318.92	2090.10
951	STX17	0.001396704	0.025739895	-0.41	0.13	6.44	0.07	0.00	0.04	0.03	0.04	915.97	584.16
952	ATP5O	0.001410416	0.025965282	0.37	0.12	7.43	0.04	0.00	0.02	0.03	0.03	1275.15	1831.33
953	RPL7	0.001414602	0.026015018	0.45	0.14	9.89	0.07	0.00	0.04	0.05	0.05	14090.21	21882.79

954	LAMP3	0.00142631	0.026175402	-1.30	0.41	1.78	0.59	0.09	0.22	0.31	0.31	21.94	4.52
955	METTL21A	0.001426079	0.026175402	0.46	0.14	5.69	0.07	0.00	0.03	0.05	0.05	208.04	328.42
956	B2M	0.001431333	0.026232314	-0.55	0.17	11.23	0.08	0.00	0.03	0.07	0.07	117625.08	69026.08
957	FBXO38	0.001432641	0.026232314	-0.40	0.13	6.36	0.06	0.00	0.03	0.03	0.03	827.61	541.68
958	FIS1	0.001433901	0.026232314	0.38	0.12	8.20	0.04	0.00	0.02	0.03	0.03	2736.67	3948.29
959	PMM1	0.001443122	0.02637346	0.50	0.16	7.12	0.10	0.00	0.06	0.03	0.06	864.53	1394.31
960	MCM2	0.001446389	0.026405641	0.66	0.21	4.63	0.13	0.00	0.05	0.10	0.10	63.66	118.57
961	PPM1K	0.001450967	0.026461649	0.54	0.17	6.76	0.11	0.00	0.07	0.03	0.07	584.23	982.85
962	TBKBP1	0.001454025	0.026489865	0.57	0.18	5.36	0.13	0.01	0.07	0.06	0.07	139.15	245.13
963	GTF2H5	0.001460045	0.026562018	0.43	0.13	7.53	0.07	0.00	0.04	0.03	0.04	1414.15	2037.36
964	HOXD9	0.001461017	0.026562018	0.86	0.27	5.13	0.29	0.01	0.17	0.07	0.17	90.88	215.39
965	ST6GALNAC4	0.001462565	0.026562602	0.92	0.29	5.07	0.33	0.01	0.19	0.07	0.19	93.75	199.93
966	DDX25	0.001467412	0.02662304	-1.44	0.45	1.78	0.81	0.11	0.38	0.31	0.38	23.52	4.84
967	HAX1	0.001469126	0.026626581	0.37	0.12	7.57	0.04	0.00	0.02	0.03	0.03	1456.91	2109.18
968	EEF1A1P6	0.001474299	0.026692726	0.61	0.19	5.03	0.12	0.01	0.05	0.07	0.07	97.83	176.08
969	TWIST2	0.00148759	0.026905583	0.93	0.29	3.88	0.34	0.02	0.18	0.15	0.18	24.51	62.92
970	MGAT2	0.001491953	0.026928906	-0.37	0.12	7.75	0.05	0.00	0.03	0.03	0.03	3219.83	2169.01
971	TUBB4B	0.001491443	0.026928906	-0.70	0.22	8.14	0.19	0.00	0.11	0.03	0.11	6161.32	3139.69
972	IFI44	0.001498864	0.027009726	-0.45	0.14	5.77	0.06	0.00	0.03	0.04	0.04	460.18	297.86
973	RPSA	0.001500703	0.027009726	0.41	0.13	9.22	0.05	0.00	0.02	0.04	0.04	7340.32	11068.04
974	SPRY4	0.001501054	0.027009726	-0.90	0.28	5.01	0.32	0.00	0.19	0.08	0.19	394.31	129.82
975	PTX4	0.001504846	0.027050188	-1.72	0.54	2.57	1.15	0.25	0.45	0.24	0.45	57.00	11.83
976	RNF43	0.001506792	0.027057421	0.50	0.16	7.48	0.10	0.00	0.06	0.03	0.06	1246.90	2006.66
977	HGH1	0.001510025	0.027087716	0.48	0.15	5.48	0.08	0.00	0.04	0.05	0.05	169.54	266.69
978	PEX10	0.001513853	0.027100905	0.46	0.14	5.93	0.08	0.00	0.05	0.04	0.05	268.14	419.13
979	SSH1	0.001513448	0.027100905	-0.50	0.16	5.41	0.08	0.00	0.03	0.06	0.06	354.16	203.02
980	HOXA2	0.001525207	0.0272763	1.88	0.59	4.28	1.39	0.05	0.79	0.13	0.79	28.61	133.61
981	XPR1	0.001533719	0.027400572	-0.44	0.14	5.77	0.05	0.00	0.02	0.04	0.04	454.81	295.65
982	TMED7-TICAM2	0.001541714	0.027515364	-0.50	0.16	6.21	0.10	0.02	0.04	0.04	0.04	794.95	458.15

983	OST4	0.001545524	0.027555302	0.38	0.12	8.08	0.05	0.00	0.03	0.03	0.03	2454.88	3511.10
984	RPL17	0.001550556	0.027616915	0.43	0.14	9.65	0.06	0.00	0.03	0.04	0.04	11264.84	17107.81
985	MCF2	0.001564564	0.027781792	-1.27	0.40	7.92	0.64	0.00	0.39	0.03	0.39	8298.55	2508.20
986	MRPL34	0.001561507	0.027781792	0.40	0.13	7.20	0.06	0.00	0.04	0.03	0.04	1025.82	1459.39
987	NEXN	0.001564569	0.027781792	0.37	0.12	6.74	0.05	0.00	0.02	0.03	0.03	629.16	921.94
988	ELL2	0.001572384	0.027821346	-0.96	0.30	4.83	0.37	0.01	0.22	0.09	0.22	356.77	108.08
989	FKBP1A	0.001573146	0.027821346	-0.37	0.12	7.61	0.04	0.00	0.01	0.03	0.03	2734.81	1893.88
990	HSPA1A	0.00157158	0.027821346	0.64	0.20	8.68	0.16	0.00	0.10	0.04	0.10	3602.15	6996.31
991	RPL27A	0.001572376	0.027821346	0.61	0.19	9.40	0.15	0.00	0.09	0.04	0.09	8390.49	13934.47
992	ABHD2	0.001575918	0.027842272	-0.42	0.13	7.86	0.07	0.00	0.04	0.03	0.04	3769.32	2406.99
993	NR0B1	0.001581187	0.027879162	0.82	0.26	6.42	0.27	0.00	0.16	0.03	0.16	357.60	763.40
994	STK38	0.00157961	0.027879162	-0.38	0.12	6.85	0.06	0.00	0.03	0.03	0.03	1304.57	883.22
995	TMEM133	0.001585824	0.027932811	-0.63	0.20	5.52	0.16	0.01	0.09	0.05	0.09	432.56	230.02
996	CASD1	0.001588006	0.027943158	0.37	0.12	7.27	0.05	0.00	0.02	0.03	0.03	1068.00	1564.76
997	SHISA4	0.001592759	0.027998696	0.59	0.19	7.65	0.14	0.00	0.08	0.03	0.08	1358.81	2445.35
998	FBXL17	0.00160026	0.028046163	-0.40	0.13	7.96	0.06	0.00	0.04	0.03	0.04	3990.52	2701.23
999	NSMCE1	0.001599929	0.028046163	0.37	0.12	6.62	0.05	0.00	0.03	0.03	0.03	577.93	811.26
1000	RPS20	0.001599569	0.028046163	0.43	0.14	9.61	0.07	0.00	0.04	0.04	0.04	10888.54	16466.10
1001	LRRC32	0.001604503	0.028092432	0.64	0.20	7.53	0.16	0.00	0.10	0.03	0.10	1313.46	2161.49
1002	PTPRF	0.001606491	0.028099155	-0.66	0.21	7.39	0.17	0.00	0.10	0.03	0.10	2826.63	1501.25
1003	AC213203.1	0.001615578	0.028229934	1.13	0.36	4.36	0.50	0.02	0.29	0.12	0.29	37.68	108.82
1004	MCL1	0.001625333	0.028372102	-0.41	0.13	8.76	0.07	0.00	0.04	0.04	0.04	9056.77	5956.83
1005	C16orf58	0.001627664	0.028384513	0.37	0.12	6.81	0.04	0.00	0.01	0.03	0.03	690.33	983.42
1006	EPSTI1	0.001633517	0.028458265	-0.65	0.21	4.93	0.17	0.01	0.09	0.08	0.09	234.86	126.64
1007	ACAD8	0.001636526	0.02846126	-0.62	0.20	7.03	0.15	0.00	0.09	0.03	0.09	2082.06	1018.70
1008	IPPK	0.001636937	0.02846126	-0.52	0.17	6.69	0.11	0.00	0.06	0.03	0.06	1256.58	746.15
1009	RSG1	0.00163913	0.028471147	0.54	0.17	5.32	0.10	0.01	0.04	0.06	0.06	134.04	232.38
1010	PPP4R3A	0.001650591	0.028641835	-0.37	0.12	7.18	0.05	0.00	0.02	0.03	0.03	1854.40	1223.74
1011	CXCL9	0.001660188	0.028779883	-1.61	0.51	2.87	1.03	0.13	0.50	0.22	0.50	100.48	15.45

1012	RSAD2	0.001662044	0.028783573	-0.88	0.28	4.21	0.31	0.01	0.18	0.13	0.18	186.33	56.85
1013	AOC4P	0.001667136	0.028843264	-1.32	0.42	2.28	0.68	0.16	0.25	0.26	0.26	35.25	8.07
1014	PPP1R37	0.001675203	0.028927568	0.46	0.15	5.74	0.08	0.00	0.05	0.04	0.05	219.82	347.91
1015	SLC30A10	0.00167531	0.028927568	-2.02	0.64	4.43	1.62	0.02	0.97	0.11	0.97	782.18	76.60
1016	KLF6	0.001678687	0.028957356	-0.62	0.20	5.65	0.15	0.01	0.09	0.05	0.09	474.11	261.90
1017	ATP6V0D2	0.00168224	0.028990107	-1.43	0.46	1.31	0.72	0.15	0.25	0.35	0.35	17.31	2.54
1018	LILRB3	0.001688263	0.029065314	-0.89	0.28	4.50	0.32	0.07	0.12	0.11	0.12	200.86	81.69
1019	ADAM12	0.001691015	0.02908413	-1.57	0.50	1.07	0.97	0.07	0.53	0.37	0.53	15.93	2.17
1020	ANP32B	0.001697706	0.029170579	-0.47	0.15	5.82	0.09	0.00	0.05	0.04	0.05	513.49	310.81
1021	HIGD2A	0.001705582	0.029277208	0.36	0.12	7.59	0.04	0.00	0.02	0.03	0.03	1506.18	2138.75
1022	DIRC3	0.001708422	0.029297256	0.77	0.25	5.09	0.24	0.02	0.13	0.07	0.13	100.59	197.91
1023	FBXL13	0.001719648	0.029460941	-0.61	0.20	5.63	0.15	0.01	0.08	0.05	0.08	471.94	254.95
1024	HAMP	0.001730405	0.029616291	-1.31	0.42	1.83	0.48	0.12	0.09	0.30	0.30	17.90	4.82
1025	PARP15	0.001732767	0.029627777	-0.75	0.24	4.26	0.17	0.01	0.07	0.13	0.13	141.46	61.66
1026	ARHGAP42	0.001738463	0.029694952	-0.70	0.22	4.87	0.19	0.01	0.11	0.08	0.11	237.77	119.48
1027	TSPAN33	0.001740084	0.029694952	0.90	0.29	5.45	0.32	0.00	0.19	0.05	0.19	137.84	292.63
1028	NHP2	0.0017439	0.029731116	0.37	0.12	6.70	0.04	0.00	0.02	0.03	0.03	608.44	877.79
1029	AURKAIP1	0.001755078	0.029852083	0.44	0.14	7.30	0.08	0.00	0.05	0.03	0.05	1083.44	1647.00
1030	EFNB1	0.001756105	0.029852083	0.56	0.18	7.16	0.12	0.00	0.07	0.03	0.07	865.40	1483.25
1031	HIST1H4E	0.001753501	0.029852083	0.38	0.12	8.53	0.06	0.00	0.03	0.04	0.04	3779.13	5548.17
1032	FBXO32	0.001763382	0.029946744	0.88	0.28	5.85	0.31	0.00	0.19	0.04	0.19	178.24	457.54
1033	CHCHD2P6	0.00177237	0.030070241	1.49	0.48	1.91	0.48	0.25	-0.14	0.30	0.30	1.59	9.07
1034	ANAPC11	0.001780471	0.030150649	0.44	0.14	6.78	0.08	0.00	0.05	0.03	0.05	644.24	976.47
1035	MRPS12	0.00178227	0.030150649	0.42	0.14	6.57	0.07	0.00	0.04	0.03	0.04	513.74	793.16
1036	PRCP	0.001781907	0.030150649	-0.36	0.12	7.40	0.04	0.00	0.02	0.03	0.03	2213.83	1535.21
1037	OSBPL11	0.001788097	0.03021787	-0.36	0.12	6.93	0.05	0.00	0.03	0.03	0.03	1372.13	962.01
1038	SPSB3	0.001789692	0.03021787	0.52	0.17	7.40	0.11	0.00	0.07	0.03	0.07	1104.45	1856.24
1039	AC025335.1	0.001793808	0.030258213	0.51	0.16	5.74	0.11	0.00	0.06	0.04	0.06	213.40	355.30
1040	TANK	0.001798748	0.030312362	-0.38	0.12	6.44	0.04	0.00	0.02	0.03	0.03	860.60	586.15

1041	ZBTB2	0.001801337	0.030326829	-0.37	0.12	6.49	0.05	0.00	0.02	0.03	0.03	893.14	616.79
1042	AFAP1	0.001819183	0.030562458	0.54	0.17	5.42	0.12	0.01	0.06	0.06	0.06	151.10	259.87
1043	ATP9B	0.001820564	0.030562458	-0.42	0.14	5.87	0.06	0.00	0.03	0.04	0.04	511.28	328.19
1044	TPSG1	0.001817819	0.030562458	1.37	0.44	2.72	0.75	0.09	0.37	0.23	0.37	5.93	22.76
1045	MRPL43	0.001843077	0.030892916	0.36	0.12	7.29	0.03	0.00	0.01	0.03	0.03	1105.11	1582.21
1046	NR0B2	0.001843774	0.030892916	1.58	0.51	5.36	1.00	0.01	0.61	0.06	0.61	138.16	336.30
1047	LMAN1L	0.001845859	0.030898297	1.33	0.43	4.20	0.71	0.03	0.41	0.13	0.41	29.41	105.21
1048	IFT20	0.0018516	0.030964835	0.38	0.12	6.53	0.04	0.00	0.02	0.03	0.03	515.94	745.52
1049	CCDC8	0.001860485	0.031067285	1.75	0.56	6.37	1.22	0.00	0.75	0.03	0.75	297.65	998.92
1050	NOCT	0.001861835	0.031067285	-0.57	0.18	5.24	0.11	0.02	0.05	0.06	0.06	299.47	174.06
1051	TMEM87A	0.001863044	0.031067285	-0.36	0.12	6.98	0.04	0.00	0.02	0.03	0.03	1475.50	999.70
1052	PRSS35	0.001865885	0.031085082	1.48	0.48	5.45	0.88	0.01	0.54	0.05	0.54	82.26	392.88
1053	NHLRC4	0.001892097	0.031491826	0.69	0.22	5.50	0.19	0.00	0.11	0.05	0.11	146.47	297.34
1054	ADRA2A	0.001900989	0.031609803	1.58	0.51	5.23	1.00	0.01	0.61	0.06	0.61	93.81	303.68
1055	AMDHD2	0.001905261	0.031650803	0.52	0.17	5.56	0.11	0.01	0.06	0.05	0.06	175.35	296.88
1056	TBC1D2	0.00191101	0.031716258	-0.49	0.16	5.46	0.10	0.01	0.05	0.05	0.05	350.86	219.09
1057	TSPOAP1	0.001919706	0.031830432	0.53	0.17	5.74	0.11	0.01	0.07	0.04	0.07	203.50	357.35
1058	CHCHD6	0.001925991	0.031874145	0.50	0.16	5.37	0.09	0.00	0.04	0.06	0.06	150.44	239.36
1059	CTAGE5	0.001927798	0.031874145	-0.47	0.15	8.41	0.09	0.00	0.06	0.04	0.06	6766.95	4190.57
1060	PDK2	0.00192454	0.031874145	0.83	0.27	7.04	0.28	0.00	0.17	0.03	0.17	720.74	1403.32
1061	FDX2	0.001939732	0.032041227	0.43	0.14	5.82	0.07	0.00	0.04	0.04	0.04	241.58	374.47
1062	LMOD1	0.001948897	0.032162312	1.21	0.39	9.08	0.59	0.00	0.37	0.04	0.37	4582.92	12277.31
1063	CYLD	0.001952636	0.032193698	-0.38	0.12	8.28	0.06	0.00	0.04	0.03	0.04	5573.62	3678.42
1064	HOMER3	0.001963455	0.032341653	0.51	0.16	5.92	0.10	0.00	0.06	0.04	0.06	253.98	424.67
1065	ASAP3	0.001973813	0.03238751	0.37	0.12	6.70	0.05	0.00	0.03	0.03	0.03	618.10	889.94
1066	DGKH	0.001975479	0.03238751	-0.52	0.17	5.57	0.11	0.01	0.06	0.05	0.06	427.46	241.13
1067	ESRRA	0.001974135	0.03238751	0.55	0.18	5.90	0.12	0.00	0.07	0.04	0.07	243.10	421.40
1068	KIF18A	0.001970679	0.03238751	-1.03	0.33	2.81	0.41	0.04	0.20	0.22	0.22	45.12	14.10
1069	MRPL2	0.001969814	0.03238751	0.38	0.12	6.43	0.05	0.00	0.02	0.03	0.03	467.28	678.28

1070	AMIGO2	0.001978232	0.03240233	-0.68	0.22	7.36	0.18	0.00	0.11	0.03	0.11	2925.99	1419.78
1071	HEG1	0.001987046	0.0324557	-0.61	0.20	5.71	0.15	0.00	0.09	0.05	0.09	520.26	278.41
1072	RP13-512J5.1	0.001985645	0.0324557	0.70	0.23	4.46	0.13	0.01	0.04	0.11	0.11	50.23	100.68
1073	WASH7P	0.001986055	0.0324557	0.52	0.17	6.25	0.11	0.00	0.06	0.04	0.06	351.91	589.11
1074	CSDC2	0.001990446	0.032478226	2.44	0.79	6.63	2.42	0.01	1.49	0.03	1.49	740.08	1461.04
1075	TCEA2	0.001992131	0.032478226	0.40	0.13	6.16	0.06	0.00	0.03	0.04	0.04	346.99	518.89
1076	EMB	0.002005174	0.032660487	-1.20	0.39	6.74	0.59	0.00	0.36	0.03	0.36	3024.13	725.98
1077	ZNF850	0.002009109	0.032694184	0.49	0.16	6.45	0.08	0.03	0.02	0.03	0.03	442.74	712.88
1078	FBXO18	0.002017751	0.032804367	-0.36	0.12	6.85	0.04	0.00	0.02	0.03	0.03	1262.48	888.42
1079	DNASE1L1	0.002022839	0.032856597	0.50	0.16	6.93	0.10	0.00	0.06	0.03	0.06	733.73	1141.12
1080	WDR31	0.002027634	0.032903987	0.56	0.18	6.17	0.13	0.00	0.08	0.04	0.08	338.89	545.75
1081	CPE	0.002049882	0.033234263	0.75	0.24	6.96	0.23	0.00	0.14	0.03	0.14	670.66	1269.21
1082	RP11-350J20.9	0.002061606	0.033393452	-1.02	0.33	3.12	0.32	0.06	0.09	0.20	0.20	51.26	19.54
1083	TNFRSF9	0.002092443	0.033861633	-1.48	0.48	2.48	0.89	0.04	0.52	0.25	0.52	105.98	8.68
1084	NSA2	0.002105382	0.034039599	0.40	0.13	7.60	0.06	0.00	0.04	0.03	0.04	1510.22	2199.75
1085	AOC2	0.002110068	0.034044954	-0.95	0.31	3.47	0.37	0.04	0.19	0.18	0.19	74.08	29.11
1086	B3GALNT2	0.002111541	0.034044954	-0.52	0.17	7.09	0.11	0.00	0.07	0.03	0.07	1995.09	1096.94
1087	WDFY3	0.002109281	0.034044954	-0.68	0.22	6.74	0.19	0.00	0.12	0.03	0.12	1797.55	752.29
1088	RP11-1277A3.2	0.002128907	0.034261915	0.42	0.14	5.82	0.05	0.00	0.02	0.04	0.04	242.99	368.26
1089	STX10	0.002127815	0.034261915	0.37	0.12	6.49	0.05	0.00	0.02	0.03	0.03	504.51	715.17
1090	SHISA9	0.002131535	0.034272731	-0.65	0.21	4.60	0.15	0.01	0.08	0.10	0.10	184.37	88.80
1091	SNTB1	0.002138544	0.034353909	1.09	0.36	7.94	0.49	0.00	0.30	0.03	0.30	2141.80	3587.24
1092	HERPUD1	0.002143184	0.034396925	-0.50	0.16	8.44	0.10	0.00	0.06	0.04	0.06	7186.30	4288.37
1093	STK16	0.002156922	0.034585747	0.37	0.12	6.84	0.04	0.00	0.02	0.03	0.03	718.35	1014.52
1094	SLC27A5	0.00216778	0.034664707	0.64	0.21	5.67	0.17	0.01	0.10	0.05	0.10	199.24	335.39
1095	SSH3	0.00216479	0.034664707	0.99	0.32	4.40	0.40	0.02	0.23	0.12	0.23	50.73	100.55
1096	STX2	0.002166122	0.034664707	-0.84	0.27	6.19	0.29	0.00	0.18	0.04	0.18	1104.83	434.43
1097	WDR37	0.002178959	0.0348117	-0.38	0.12	6.35	0.05	0.00	0.03	0.03	0.03	783.71	536.54
1098	PTGES2	0.002184623	0.034870411	0.36	0.12	7.20	0.04	0.00	0.02	0.03	0.03	1010.77	1445.18

1099	PDE9A	0.002193968	0.034964052	0.47	0.15	6.02	0.09	0.00	0.05	0.04	0.05	286.71	462.81
1100	RNASEK	0.00219448	0.034964052	0.47	0.15	8.30	0.09	0.00	0.06	0.03	0.06	2943.72	4486.04
1101	CUTA	0.002209058	0.035164358	0.47	0.15	8.57	0.09	0.00	0.06	0.04	0.06	3835.62	5918.41
1102	MFAP3	0.002213874	0.03517913	-0.41	0.13	5.92	0.04	0.00	0.01	0.04	0.04	519.80	345.12
1103	NTM	0.002214001	0.03517913	-2.43	0.79	3.29	2.41	0.06	1.45	0.19	1.45	429.28	27.24
1104	COLCA2	0.002225267	0.03529414	0.95	0.31	3.59	0.36	0.06	0.16	0.17	0.17	18.48	46.87
1105	IFIT2	0.00222387	0.03529414	-0.51	0.17	7.27	0.11	0.00	0.07	0.03	0.07	2420.65	1313.76
1106	ACVR1B	0.002233298	0.035389489	-0.41	0.13	5.95	0.05	0.00	0.02	0.04	0.04	536.56	355.66
1107	DOK4	0.002254157	0.035687767	0.67	0.22	4.51	0.12	0.01	0.04	0.11	0.11	55.30	104.84
1108	EIF3J	0.002261817	0.035726176	-0.36	0.12	6.79	0.03	0.00	0.01	0.03	0.03	1193.78	826.98
1109	FTCDNL1	0.002262699	0.035726176	0.58	0.19	5.18	0.14	0.01	0.08	0.07	0.08	115.82	207.17
1110	SRM	0.002261909	0.035726176	0.43	0.14	5.70	0.06	0.00	0.03	0.05	0.05	217.06	328.71
1111	KIAA1161	0.002284195	0.036033122	-0.71	0.23	6.81	0.21	0.00	0.13	0.03	0.13	1717.26	823.75
1112	MTHFD2	0.002294711	0.036166458	-0.68	0.22	5.29	0.19	0.01	0.11	0.06	0.11	359.99	181.26
1113	AC012314.11	0.002320044	0.036303516	0.47	0.15	7.02	0.09	0.00	0.06	0.03	0.06	785.10	1260.95
1114	AC012314.12	0.002320044	0.036303516	0.47	0.15	7.02	0.09	0.00	0.06	0.03	0.06	785.10	1260.95
1115	AC012314.14	0.002320044	0.036303516	0.47	0.15	7.02	0.09	0.00	0.06	0.03	0.06	785.10	1260.95
1116	AC012314.2	0.002320044	0.036303516	0.47	0.15	7.02	0.09	0.00	0.06	0.03	0.06	785.10	1260.95
1117	AC012314.4	0.002320044	0.036303516	0.47	0.15	7.02	0.09	0.00	0.06	0.03	0.06	785.10	1260.95
1118	AC012314.5	0.002320044	0.036303516	0.47	0.15	7.02	0.09	0.00	0.06	0.03	0.06	785.10	1260.95
1119	DCTD	0.002307154	0.036303516	0.35	0.12	7.36	0.04	0.00	0.02	0.03	0.03	1185.22	1694.40
1120	FAM122A	0.002314212	0.036303516	-0.35	0.12	7.46	0.04	0.00	0.02	0.03	0.03	2387.40	1631.95
1121	FXYD1	0.002325446	0.036303516	1.22	0.40	4.41	0.61	0.02	0.36	0.12	0.36	42.21	118.78
1122	SLC25A33	0.002326192	0.036303516	-0.54	0.18	5.83	0.12	0.00	0.07	0.04	0.07	525.89	315.23
1123	WNK1	0.002325706	0.036303516	-0.36	0.12	7.04	0.05	0.00	0.03	0.03	0.03	1537.71	1075.81
1124	OAF	0.002330622	0.036340287	0.40	0.13	6.92	0.07	0.00	0.04	0.03	0.04	767.64	1110.27
1125	AKAP1	0.002350365	0.036499161	0.46	0.15	5.48	0.07	0.00	0.03	0.05	0.05	171.60	264.32
1126	DGKA	0.002351224	0.036499161	-0.65	0.21	5.77	0.17	0.01	0.10	0.04	0.10	566.04	293.59
1127	ORAI2	0.002343672	0.036499161	0.65	0.21	6.04	0.17	0.01	0.10	0.04	0.10	282.00	489.17

1128	TBC1D8	0.00234848	0.036499161	-0.55	0.18	5.05	0.11	0.00	0.06	0.07	0.07	256.13	142.28
1129	WBP1L	0.002348016	0.036499161	-0.38	0.13	8.85	0.05	0.00	0.03	0.04	0.04	9505.53	6561.03
1130	SRC	0.002355784	0.036537583	0.46	0.15	5.97	0.09	0.00	0.05	0.04	0.05	287.64	435.15
1131	CDCA3	0.002369589	0.036719205	-0.82	0.27	4.04	0.24	0.04	0.10	0.14	0.14	124.00	49.76
1132	ABHD10	0.002380956	0.036830211	0.35	0.12	6.78	0.04	0.00	0.02	0.03	0.03	665.57	955.62
1133	CNPPD1	0.002379004	0.036830211	0.49	0.16	7.87	0.10	0.00	0.06	0.03	0.06	1978.38	2896.38
1134	BTBD7	0.002393063	0.03698485	-0.37	0.12	6.61	0.05	0.00	0.03	0.03	0.03	1047.10	695.80
1135	NPIPB4	0.002395431	0.036985407	-0.46	0.15	5.63	0.07	0.01	0.03	0.05	0.05	404.32	259.04
1136	SCARB2	0.00239732	0.036985407	-0.35	0.12	7.36	0.03	0.00	0.01	0.03	0.03	2085.58	1477.22
1137	ATXN7	0.002406215	0.037088617	-0.54	0.18	5.17	0.10	0.01	0.05	0.07	0.07	276.49	160.91
1138	EPG5	0.002410358	0.037088617	-0.36	0.12	7.65	0.04	0.00	0.02	0.03	0.03	2842.32	1975.13
1139	HIST1H4D	0.002408958	0.037088617	0.49	0.16	6.99	0.10	0.00	0.06	0.03	0.06	785.75	1212.06
1140	CRB1	0.002418532	0.037115428	1.19	0.39	3.71	0.58	0.07	0.30	0.16	0.30	19.46	58.59
1141	PLA2G7	0.002419891	0.037115428	-1.62	0.53	4.58	1.09	0.02	0.67	0.10	0.67	422.04	92.51
1142	PWP2	0.002414908	0.037115428	-1.22	0.40	3.68	0.61	0.18	0.21	0.16	0.21	108.33	38.54
1143	RPL7A	0.002420571	0.037115428	0.47	0.16	10.67	0.06	0.00	0.02	0.06	0.06	29825.81	47455.56
1144	IDS	0.002427838	0.03719431	-0.42	0.14	6.96	0.07	0.00	0.04	0.03	0.04	1540.95	985.69
1145	NIPA2	0.002430907	0.037208798	-0.35	0.12	6.98	0.05	0.00	0.03	0.03	0.03	1480.54	1008.45
1146	DLAT	0.002436983	0.037269259	0.35	0.12	7.63	0.04	0.00	0.02	0.03	0.03	1551.28	2222.95
1147	GPR162	0.002443242	0.037332392	-1.20	0.40	3.52	0.60	0.06	0.32	0.17	0.32	139.47	28.83
1148	WARS2	0.002470499	0.037715998	0.41	0.14	5.88	0.05	0.00	0.02	0.04	0.04	267.91	389.33
1149	CAMKK1	0.00248192	0.037791592	0.79	0.26	5.97	0.26	0.00	0.16	0.04	0.16	260.57	468.66
1150	EPHA2	0.002479829	0.037791592	0.70	0.23	4.37	0.21	0.01	0.12	0.12	0.12	47.98	95.89
1151	TMEM201	0.002480691	0.037791592	0.56	0.19	6.05	0.13	0.00	0.08	0.04	0.08	300.40	481.84
1152	HEMK1	0.00250348	0.03805377	0.47	0.16	7.12	0.09	0.00	0.06	0.03	0.06	884.91	1386.21
1153	MAPK14	0.002503444	0.03805377	-0.35	0.12	7.08	0.03	0.00	0.01	0.03	0.03	1595.05	1115.64
1154	CHST4	0.002507962	0.038088857	1.56	0.52	4.27	1.01	0.03	0.61	0.13	0.61	33.54	120.40
1155	MBOAT1	0.002512174	0.038119789	1.04	0.35	5.52	0.45	0.01	0.28	0.05	0.28	138.48	334.86
1156	RASEF	0.002524124	0.038267983	-0.57	0.19	6.47	0.13	0.00	0.08	0.03	0.08	1039.27	599.23

1157	EIF3H	0.002535424	0.038406088	0.37	0.12	8.26	0.06	0.00	0.04	0.03	0.04	2905.50	4204.43
1158	HDAC9	0.002543185	0.038439497	-0.93	0.31	5.44	0.36	0.01	0.22	0.05	0.22	586.84	206.94
1159	TPD52	0.00254421	0.038439497	1.21	0.40	5.31	0.61	0.01	0.38	0.06	0.38	104.25	290.93
1160	UQCC2	0.002542426	0.038439497	0.36	0.12	7.65	0.05	0.00	0.02	0.03	0.03	1588.71	2293.02
1161	LAYN	0.002556607	0.038593542	-1.56	0.52	4.40	1.01	0.03	0.61	0.12	0.61	894.89	62.17
1162	DDX60L	0.002564776	0.038650278	-0.67	0.22	4.54	0.16	0.01	0.08	0.11	0.11	168.77	83.75
1163	GYPC	0.002563941	0.038650278	0.83	0.28	7.51	0.29	0.00	0.18	0.03	0.18	1204.71	2184.47
1164	UNC5B	0.00258752	0.038959513	0.49	0.16	6.26	0.10	0.00	0.06	0.04	0.06	364.75	593.68
1165	PODN	0.002595652	0.039048407	1.07	0.36	5.72	0.48	0.00	0.30	0.04	0.30	158.47	427.90
1166	INMT	0.002601729	0.039106268	0.91	0.30	7.07	0.34	0.00	0.22	0.03	0.22	684.88	1497.23
1167	CADM3	0.002608131	0.039135367	-3.00	1.00	2.74	3.76	0.24	2.15	0.23	2.15	1187.21	18.40
1168	RPL41	0.002607735	0.039135367	0.47	0.16	10.65	0.07	0.00	0.03	0.06	0.06	30132.60	46838.40
1169	BAG5	0.00261114	0.039146992	-0.37	0.12	7.47	0.06	0.00	0.03	0.03	0.03	2523.36	1640.68
1170	ZGPAT	0.002619778	0.039242938	0.53	0.18	6.23	0.12	0.00	0.07	0.04	0.07	356.81	572.23
1171	AZIN2	0.00262266	0.03925255	0.68	0.23	4.84	0.19	0.02	0.11	0.09	0.11	81.97	148.69
1172	MYO5A	0.002635369	0.039409112	-0.36	0.12	7.53	0.05	0.00	0.03	0.03	0.03	2521.45	1759.22
1173	MYO9B	0.002639077	0.039410655	-0.47	0.16	5.79	0.09	0.00	0.06	0.04	0.06	498.14	304.04
1174	TBCE	0.00263997	0.039410655	-0.36	0.12	7.39	0.06	0.00	0.03	0.03	0.03	2274.16	1522.28
1175	CARD19	0.002646979	0.039481667	0.48	0.16	5.81	0.10	0.00	0.06	0.04	0.06	227.90	378.30
1176	MRPS7	0.002649681	0.039488363	0.35	0.12	7.45	0.04	0.00	0.02	0.03	0.03	1321.07	1867.45
1177	FOXRED1	0.002652547	0.039497489	0.48	0.16	5.99	0.10	0.00	0.06	0.04	0.06	279.91	452.77
1178	CASP3	0.00265492	0.03949926	-0.36	0.12	6.59	0.05	0.00	0.02	0.03	0.03	988.41	684.51
1179	GPR22	0.002666734	0.03964138	-1.15	0.38	3.47	0.55	0.06	0.30	0.18	0.30	97.46	28.70
1180	TXK	0.0026765	0.039752835	-0.85	0.28	3.82	0.26	0.04	0.11	0.15	0.15	93.76	40.50
1181	BAG2	0.002679307	0.039760821	0.85	0.28	6.91	0.30	0.00	0.19	0.03	0.19	640.32	1224.81
1182	FAM213B	0.002685674	0.039787931	0.64	0.21	5.88	0.17	0.00	0.11	0.04	0.11	224.38	425.01
1183	HMGCL	0.002684769	0.039787931	0.39	0.13	7.83	0.06	0.00	0.04	0.03	0.04	1884.16	2772.37
1184	WWC2	0.002691128	0.039835061	-0.35	0.12	7.37	0.05	0.00	0.03	0.03	0.03	2152.74	1501.21
1185	MPND	0.002697071	0.039889337	0.45	0.15	6.47	0.08	0.01	0.05	0.03	0.05	469.83	715.59

1186	C9orf84	0.002703642	0.039904284	-1.95	0.65	6.68	1.60	0.01	1.01	0.03	1.01	5875.02	807.71
1187	RGMA	0.002704912	0.039904284	1.16	0.39	4.17	0.56	0.02	0.33	0.13	0.33	28.01	94.43
1188	SIMC1	0.002701496	0.039904284	0.36	0.12	6.45	0.05	0.00	0.03	0.03	0.03	486.22	684.08
1189	NPRL2	0.002714138	0.040006715	0.47	0.16	6.59	0.09	0.00	0.06	0.03	0.06	504.57	820.88
1190	CYP4B1	0.002728267	0.040129893	1.59	0.53	5.27	1.06	0.01	0.66	0.06	0.66	63.17	352.70
1191	IL15	0.00272711	0.040129893	-0.90	0.30	4.24	0.34	0.03	0.18	0.13	0.18	174.34	61.51
1192	ZNF444P1	0.002729364	0.040129893	1.71	0.57	2.50	1.22	0.28	0.50	0.25	0.50	6.56	19.21
1193	FNDC3A	0.002741066	0.040268166	-0.40	0.13	9.56	0.06	0.00	0.04	0.04	0.04	20587.27	13227.02
1194	H1FX	0.002746525	0.040314573	0.54	0.18	6.77	0.12	0.00	0.08	0.03	0.08	597.39	993.90
1195	ARMCX3	0.002749561	0.040325364	-0.47	0.16	7.68	0.09	0.00	0.06	0.03	0.06	3362.69	1997.10
1196	DSTYK	0.002753889	0.040330722	-0.35	0.12	7.94	0.04	0.00	0.02	0.03	0.03	3793.09	2614.06
1197	THAP8	0.002754529	0.040330722	0.73	0.24	4.20	0.14	0.01	0.04	0.13	0.13	37.13	78.77
1198	INSR	0.002757967	0.040347357	-0.56	0.19	7.01	0.13	0.00	0.08	0.03	0.08	1909.10	1016.85
1199	PARD3	0.00276369	0.040378873	-0.37	0.12	8.57	0.05	0.00	0.02	0.04	0.04	7276.46	4951.69
1200	YAF2	0.002764729	0.040378873	-0.48	0.16	5.40	0.08	0.00	0.04	0.06	0.06	346.84	202.13
1201	METRN	0.002771695	0.040413253	1.02	0.34	5.28	0.43	0.01	0.27	0.06	0.27	99.69	266.87
1202	ODC1	0.002770889	0.040413253	0.46	0.15	6.36	0.09	0.00	0.05	0.03	0.05	420.36	644.26
1203	GBA	0.002778965	0.040485572	0.47	0.16	7.14	0.09	0.00	0.06	0.03	0.06	906.50	1422.42
1204	CNTNAP5	0.002784809	0.040537005	-1.37	0.46	0.13	0.60	0.04	0.27	0.46	0.46	5.78	0.38
1205	SPTLC3	0.002791392	0.040599115	-0.57	0.19	5.03	0.14	0.01	0.08	0.07	0.08	265.45	138.27
1206	PLD6	0.002810587	0.040844394	-1.17	0.39	6.23	0.58	0.00	0.37	0.04	0.37	1649.68	456.25
1207	DUS3L	0.002825635	0.041029066	0.48	0.16	5.61	0.09	0.01	0.03	0.05	0.05	187.79	307.32
1208	CYB5B	0.002832034	0.04105214	0.56	0.19	10.29	0.13	0.00	0.09	0.05	0.09	20854.42	33485.52
1209	EFR3A	0.002835396	0.04105214	-0.34	0.12	6.81	0.03	0.00	0.01	0.03	0.03	1199.56	854.26
1210	EIF3L	0.002837539	0.04105214	0.37	0.13	8.77	0.05	0.00	0.03	0.04	0.04	4883.68	6999.55
1211	IGFBP5	0.002838936	0.04105214	0.60	0.20	8.08	0.15	0.00	0.10	0.03	0.10	2145.28	3746.45
1212	OSR1	0.002836523	0.04105214	1.81	0.61	2.81	1.38	0.12	0.76	0.22	0.76	8.52	26.96
1213	CENPP	0.00284265	0.04107195	-0.63	0.21	5.63	0.16	0.03	0.08	0.05	0.08	484.52	256.39
1214	ZIM2	0.002850475	0.041151086	0.58	0.20	6.83	0.14	0.00	0.09	0.03	0.09	660.68	1052.34

1215	HDDC3	0.00286387	0.04124424	0.47	0.16	6.50	0.09	0.00	0.06	0.03	0.06	499.17	741.71
1216	LHFPL1	0.002863987	0.04124424	-1.95	0.65	3.47	1.61	0.04	0.99	0.18	0.99	276.89	29.55
1217	MAP1LC3B	0.002859833	0.04124424	-0.45	0.15	6.55	0.09	0.00	0.05	0.03	0.05	1034.65	650.53
1218	OLFML3	0.00288491	0.041511444	1.08	0.36	6.99	0.50	0.00	0.32	0.03	0.32	614.60	1469.52
1219	FAM188B2	0.00290918	0.041826326	1.00	0.34	3.12	0.39	0.07	0.16	0.20	0.20	12.60	29.13
1220	CERS4	0.002928484	0.041975605	0.84	0.28	5.75	0.30	0.00	0.19	0.04	0.19	160.07	408.31
1221	DHX36	0.002925472	0.041975605	-0.36	0.12	6.54	0.06	0.00	0.03	0.03	0.03	939.83	655.23
1222	KIAA1549	0.002923824	0.041975605	-0.59	0.20	7.11	0.15	0.00	0.09	0.03	0.09	2085.79	1124.32
1223	TSPAN5	0.002929143	0.041975605	-1.30	0.44	2.75	0.72	0.05	0.41	0.23	0.41	60.46	14.28
1224	RP11-1016B18.1	0.002933872	0.041977756	-1.63	0.55	1.50	1.13	0.09	0.63	0.33	0.63	16.64	5.13
1225	ST6GAL1	0.002934084	0.041977756	-0.59	0.20	5.95	0.15	0.00	0.09	0.04	0.09	659.74	352.59
1226	C9orf142	0.00294019	0.04203081	0.52	0.17	5.51	0.11	0.00	0.07	0.05	0.07	166.88	282.14
1227	IL27RA	0.002949146	0.042124471	-0.63	0.21	5.01	0.17	0.01	0.10	0.08	0.10	260.07	137.11
1228	LUZP1	0.002967073	0.042311577	0.38	0.13	8.05	0.06	0.00	0.04	0.03	0.04	2335.73	3459.53
1229	TM4SF1	0.002965497	0.042311577	0.55	0.19	7.55	0.13	0.00	0.08	0.03	0.08	1345.87	2166.96
1230	EXD3	0.002989469	0.042576894	0.50	0.17	5.30	0.10	0.01	0.05	0.06	0.06	136.19	226.82
1231	NR4A3	0.002990537	0.042576894	-1.70	0.57	7.49	1.23	0.00	0.79	0.03	0.79	8327.31	1860.73
1232	FAU	0.003000321	0.042681512	0.38	0.13	8.95	0.05	0.00	0.02	0.04	0.04	5965.92	8376.89
1233	ANO7	0.003004894	0.0427119	0.80	0.27	3.99	0.14	0.03	0.00	0.14	0.14	27.99	63.83
1234	SYF2	0.003016157	0.042837254	0.35	0.12	7.55	0.04	0.00	0.02	0.03	0.03	1458.76	2059.24
1235	SHC1	0.003029667	0.042994291	0.35	0.12	7.65	0.03	0.00	0.00	0.03	0.03	1585.49	2244.12
1236	ZNF835	0.0030554	0.043324387	0.69	0.23	4.74	0.19	0.04	0.08	0.09	0.09	71.10	137.08
1237	GTF2A1	0.003059731	0.043350718	-0.37	0.12	6.93	0.06	0.00	0.04	0.03	0.04	1443.25	953.83
1238	LINS1	0.00307289	0.043501993	-0.48	0.16	5.49	0.10	0.01	0.06	0.05	0.06	388.03	223.14
1239	DPH1	0.003078674	0.043545267	0.37	0.12	6.26	0.06	0.00	0.03	0.04	0.04	405.24	567.42
1240	GABBR2	0.003085962	0.043545267	-1.84	0.62	1.92	1.44	0.13	0.80	0.30	0.80	65.72	5.85
1241	GUCY1A2	0.003081929	0.043545267	0.80	0.27	6.72	0.27	0.00	0.17	0.03	0.17	584.00	1002.60
1242	NAGLU	0.00308837	0.043545267	0.50	0.17	6.33	0.11	0.00	0.07	0.03	0.07	395.91	638.43
1243	NDUFA10	0.003085879	0.043545267	0.35	0.12	7.85	0.05	0.00	0.03	0.03	0.03	1958.28	2797.62

1244	LGALS1	0.003097386	0.043567247	0.56	0.19	7.99	0.13	0.00	0.09	0.03	0.09	2046.20	3399.65
1245	TRIO	0.003096362	0.043567247	-0.37	0.13	6.26	0.06	0.00	0.03	0.04	0.04	745.01	488.13
1246	TVP23C-CDRT4	0.003094372	0.043567247	0.68	0.23	5.15	0.20	0.01	0.12	0.07	0.12	100.50	211.85
1247	CRIM1	0.003106306	0.043657677	0.46	0.15	5.42	0.06	0.00	0.02	0.06	0.06	158.41	250.25
1248	CYP4F35P	0.003117421	0.043778782	-2.20	0.75	1.92	2.07	0.19	1.14	0.30	1.14	42.17	12.37
1249	RNF208	0.003122342	0.043812784	0.90	0.30	3.99	0.34	0.03	0.19	0.14	0.19	26.09	71.35
1250	MAF1	0.003128067	0.043858008	0.37	0.12	7.40	0.06	0.00	0.04	0.03	0.04	1268.63	1774.86
1251	RPS6	0.003139081	0.043977241	0.45	0.15	10.19	0.09	0.00	0.05	0.05	0.05	19107.67	29854.01
1252	AQP3	0.003152009	0.044123086	-1.96	0.66	2.37	1.64	0.16	0.90	0.26	0.90	62.99	10.07
1253	DIRC2	0.003162458	0.044198762	-0.52	0.18	7.19	0.12	0.00	0.07	0.03	0.07	2289.87	1205.30
1254	IGSF1	0.003161768	0.044198762	-1.51	0.51	2.34	0.98	0.22	0.41	0.26	0.41	42.50	9.31
1255	C7orf31	0.003181337	0.044286032	0.73	0.25	4.39	0.23	0.01	0.14	0.12	0.14	52.87	96.61
1256	CPA4	0.003172375	0.044286032	1.23	0.42	3.29	0.65	0.03	0.39	0.19	0.39	16.84	36.69
1257	FABP4	0.003180239	0.044286032	-2.03	0.69	3.92	1.77	0.05	1.09	0.15	1.09	266.10	60.87
1258	GMFB	0.003179668	0.044286032	-0.35	0.12	6.53	0.05	0.00	0.03	0.03	0.03	948.76	642.59
1259	NRBF2	0.003178176	0.044286032	-0.36	0.12	6.31	0.04	0.00	0.02	0.03	0.03	742.19	513.54
1260	CALN1	0.003190342	0.044376136	0.66	0.22	9.53	0.19	0.00	0.12	0.04	0.12	8698.01	16396.91
1261	DALRD3	0.003193131	0.044379714	0.41	0.14	6.61	0.07	0.00	0.04	0.03	0.04	553.49	816.72
1262	C12orf50	0.003201775	0.044429378	-0.61	0.21	4.68	0.13	0.01	0.07	0.10	0.10	181.75	98.13
1263	CYB5R2	0.003199577	0.044429378	-1.25	0.43	3.53	0.67	0.03	0.40	0.17	0.40	100.65	32.22
1264	FDX1	0.003211185	0.044524708	-0.60	0.20	10.60	0.16	0.00	0.10	0.06	0.10	65178.82	37527.53
1265	WASH2P	0.003214337	0.044533178	0.41	0.14	5.93	0.07	0.01	0.04	0.04	0.04	279.31	415.15
1266	NPY5R	0.003224914	0.044644432	0.81	0.28	4.99	0.28	0.01	0.17	0.08	0.17	91.73	182.54
1267	ARAP2	0.003234746	0.044674667	-1.37	0.46	4.71	0.80	0.02	0.50	0.09	0.50	412.25	104.66
1268	DAAM2	0.003231449	0.044674667	-0.61	0.21	8.08	0.16	0.00	0.10	0.03	0.10	5706.60	2973.37
1269	ZNF148	0.003233978	0.044674667	-0.39	0.13	7.48	0.06	0.00	0.04	0.03	0.04	2544.88	1644.66
1270	AKIRIN1	0.003240384	0.044717302	-0.34	0.12	6.97	0.04	0.00	0.02	0.03	0.03	1430.03	1007.74
1271	C3orf52	0.003249645	0.044809814	-1.54	0.52	3.61	1.01	0.03	0.62	0.17	0.62	152.10	34.23
1272	ENPP5	0.003254914	0.044847189	-0.97	0.33	6.72	0.40	0.00	0.26	0.03	0.26	1993.92	765.02

1273	LRRC20	0.003266932	0.04497741	0.39	0.13	6.36	0.05	0.01	0.01	0.03	0.03	424.12	628.56
1274	FAP	0.003275166	0.045055383	-2.07	0.70	2.61	1.84	0.18	1.01	0.24	1.01	161.69	11.19
1275	DDC	0.003291647	0.045231329	1.33	0.45	4.24	0.76	0.04	0.45	0.13	0.45	31.88	110.54
1276	TIMM13	0.003293117	0.045231329	0.50	0.17	7.82	0.11	0.00	0.07	0.03	0.07	1691.95	2837.86
1277	RIC8B	0.003296203	0.045238263	-0.38	0.13	6.06	0.04	0.00	0.01	0.04	0.04	583.60	398.12
1278	IER3	0.00331896	0.045514936	0.93	0.32	5.72	0.37	0.00	0.23	0.04	0.23	169.24	397.34
1279	CHI3L1	0.003331076	0.045645384	-1.61	0.55	1.97	1.11	0.15	0.58	0.29	0.58	48.19	5.78
1280	HAAO	0.003337903	0.045703191	0.66	0.22	4.97	0.18	0.01	0.11	0.08	0.11	90.74	170.66
1281	PAPLN	0.003346361	0.045783228	0.97	0.33	6.26	0.41	0.01	0.26	0.04	0.26	274.87	709.99
1282	MAB21L3	0.003356845	0.045890843	-0.69	0.24	5.02	0.21	0.01	0.13	0.07	0.13	292.96	137.28
1283	PRRG3	0.003369028	0.046021504	-1.64	0.56	1.92	1.17	0.03	0.72	0.30	0.72	59.72	4.84
1284	NRG4	0.00337196	0.046025675	-0.79	0.27	4.54	0.27	0.04	0.13	0.11	0.13	196.37	84.74
1285	CADM4	0.003385948	0.046117796	0.43	0.15	6.34	0.08	0.01	0.04	0.03	0.04	408.23	628.80
1286	DGKI	0.003386676	0.046117796	-0.84	0.29	3.66	0.29	0.03	0.15	0.16	0.16	80.64	35.07
1287	RPS11	0.003386034	0.046117796	0.42	0.14	10.02	0.05	0.00	0.02	0.05	0.05	16494.41	24526.26
1288	TMEM261	0.003389234	0.046117796	0.35	0.12	6.62	0.05	0.00	0.03	0.03	0.03	574.17	818.91
1289	MALSU1	0.003398477	0.046207683	0.36	0.12	6.45	0.03	0.00	0.01	0.03	0.03	477.97	678.32
1290	ZNF593	0.003402345	0.046224415	0.49	0.17	5.74	0.11	0.00	0.06	0.04	0.06	218.65	351.46
1291	EVA1B	0.003427286	0.046485909	0.69	0.23	5.01	0.20	0.01	0.13	0.08	0.13	93.76	179.59
1292	KCNJ12	0.003425165	0.046485909	-2.11	0.72	3.68	1.93	0.05	1.20	0.16	1.20	398.77	51.85
1293	ZNF581	0.003429549	0.046485909	0.40	0.14	5.87	0.05	0.00	0.02	0.04	0.04	261.01	387.51
1294	LRRC24	0.003451197	0.04667538	1.43	0.49	2.96	0.88	0.13	0.44	0.21	0.44	9.56	29.43
1295	PAK3	0.003447866	0.04667538	1.44	0.49	4.70	0.90	0.01	0.57	0.09	0.57	51.28	176.11
1296	SNX15	0.003451517	0.04667538	0.46	0.16	5.43	0.08	0.00	0.04	0.06	0.06	159.96	254.29
1297	FBXO6	0.003466441	0.046841057	0.59	0.20	5.37	0.15	0.00	0.09	0.06	0.09	148.82	245.48
1298	SGK1	0.003472219	0.046882983	0.92	0.31	7.10	0.36	0.00	0.24	0.03	0.24	630.82	1605.23
1299	ASTN1	0.003477571	0.04688301	0.36	0.12	8.58	0.04	0.00	0.02	0.04	0.04	4114.12	5768.59
1300	CDK4	0.003476524	0.04688301	0.49	0.17	7.54	0.10	0.00	0.07	0.03	0.07	1365.00	2114.29
1301	TSC22D2	0.003498879	0.047134013	-0.40	0.14	5.89	0.06	0.00	0.03	0.04	0.04	502.81	337.21

1302	IL6R	0.003527513	0.047483251	0.51	0.17	5.16	0.11	0.00	0.06	0.07	0.07	117.45	198.74
1303	IFI27L2	0.003533587	0.047528514	0.41	0.14	6.95	0.07	0.00	0.05	0.03	0.05	767.64	1151.48
1304	PTPRH	0.003545107	0.047610375	-0.38	0.13	6.14	0.06	0.00	0.04	0.04	0.04	641.02	439.11
1305	SH3TC2	0.003543806	0.047610375	1.06	0.36	3.32	0.32	0.13	0.01	0.19	0.19	12.22	35.68
1306	ACOT4	0.003563984	0.04768408	-0.99	0.34	3.30	0.42	0.06	0.22	0.19	0.22	72.05	24.31
1307	GRPEL2	0.003566057	0.04768408	-0.36	0.12	6.34	0.05	0.00	0.03	0.03	0.03	774.87	533.81
1308	IDH3B	0.00356964	0.04768408	0.35	0.12	7.58	0.05	0.00	0.03	0.03	0.03	1509.56	2138.74
1309	LZTS2	0.0035676	0.04768408	0.34	0.12	6.67	0.04	0.00	0.02	0.03	0.03	608.06	849.01
1310	MAP2	0.003555963	0.04768408	0.67	0.23	4.38	0.17	0.01	0.09	0.12	0.12	50.20	93.76
1311	NUMB	0.003558298	0.04768408	-0.34	0.12	6.96	0.04	0.00	0.02	0.03	0.03	1433.55	986.88
1312	PKN1	0.003569265	0.04768408	0.35	0.12	6.30	0.04	0.00	0.02	0.03	0.03	415.46	590.62
1313	DCT	0.003573565	0.047700153	0.87	0.30	3.71	0.31	0.05	0.14	0.16	0.16	21.42	52.11
1314	MTA3	0.003591569	0.047901458	0.37	0.13	6.85	0.06	0.00	0.04	0.03	0.04	750.15	1018.56
1315	RPS28	0.003596433	0.047901458	0.40	0.14	9.73	0.05	0.00	0.03	0.05	0.05	12413.99	18517.02
1316	ZNF214	0.003596846	0.047901458	0.69	0.24	4.45	0.18	0.02	0.08	0.11	0.11	53.88	100.63
1317	ATP5F1P5	0.003615532	0.048063035	1.47	0.50	1.53	0.60	0.28	-0.03	0.33	0.33	1.57	5.95
1318	BIRC2	0.003614263	0.048063035	-0.35	0.12	6.44	0.03	0.00	0.01	0.03	0.03	828.91	586.31
1319	EFHC1	0.003620512	0.048063035	-0.39	0.14	6.74	0.07	0.01	0.04	0.03	0.04	1186.00	791.46
1320	FANCE	0.00362269	0.048063035	0.63	0.22	4.59	0.12	0.01	0.05	0.10	0.10	63.05	112.53
1321	STX16	0.00362219	0.048063035	-0.41	0.14	5.77	0.04	0.00	0.01	0.04	0.04	446.74	298.79
1322	SMCHD1	0.003632419	0.048155659	-0.40	0.14	5.86	0.07	0.00	0.04	0.04	0.04	483.59	328.72
1323	FAM105A	0.003639848	0.048215329	-0.65	0.22	4.48	0.15	0.01	0.07	0.11	0.11	148.96	80.15
1324	NPY1R	0.003642422	0.048215329	0.73	0.25	6.28	0.23	0.00	0.15	0.03	0.15	370.43	635.22
1325	SLC19A1	0.003647332	0.048243876	-0.72	0.25	4.74	0.22	0.02	0.13	0.09	0.13	215.26	104.75
1326	ADH5	0.00365623	0.048307597	0.34	0.12	7.58	0.05	0.00	0.03	0.03	0.03	1550.26	2107.16
1327	BLOC1S2	0.003658016	0.048307597	-0.34	0.12	7.48	0.04	0.00	0.02	0.03	0.03	2377.83	1669.47
1328	STK40	0.003660418	0.048307597	-0.36	0.12	6.67	0.04	0.01	0.02	0.03	0.03	1069.49	739.97
1329	PREB	0.003668634	0.048379598	0.34	0.12	7.01	0.05	0.00	0.03	0.03	0.03	860.51	1199.24
1330	CNPY4	0.003685698	0.048379713	0.36	0.12	6.27	0.04	0.00	0.02	0.03	0.03	395.93	571.39

1331	KAZALD1	0.003691026	0.048379713	0.90	0.31	3.33	0.30	0.04	0.13	0.19	0.19	15.35	34.65
1332	KRAS	0.003683162	0.048379713	-0.39	0.13	6.19	0.07	0.00	0.04	0.04	0.04	722.43	454.14
1333	MAP1B	0.003679329	0.048379713	-1.13	0.39	7.06	0.55	0.00	0.36	0.03	0.36	3450.63	1075.65
1334	MRPL27	0.003698713	0.048379713	0.34	0.12	7.39	0.04	0.00	0.02	0.03	0.03	1268.93	1749.25
1335	NT5C3B	0.003699008	0.048379713	0.34	0.12	6.77	0.03	0.00	0.01	0.03	0.03	665.50	944.33
1336	OTULIN	0.003695264	0.048379713	-0.54	0.19	5.09	0.10	0.01	0.04	0.07	0.07	257.36	147.76
1337	PNRC2	0.003678	0.048379713	0.33	0.11	7.34	0.03	0.00	0.01	0.03	0.03	1186.10	1651.52
1338	SSR2	0.003689185	0.048379713	0.36	0.12	8.80	0.05	0.00	0.03	0.04	0.04	4978.10	7216.46
1339	THYN1	0.003671465	0.048379713	0.34	0.12	7.08	0.04	0.00	0.02	0.03	0.03	925.31	1273.42
1340	TOX	0.003685849	0.048379713	-1.94	0.67	3.18	1.64	0.09	0.98	0.20	0.98	232.37	21.33
1341	ZNF711	0.003706647	0.048443472	0.79	0.27	8.77	0.27	0.00	0.18	0.04	0.18	4111.56	7923.30
1342	JUNB	0.003713102	0.048491676	1.22	0.42	8.08	0.65	0.00	0.43	0.03	0.43	1320.56	4993.80
1343	NUDT10	0.003716504	0.048499968	-0.96	0.33	3.72	0.40	0.04	0.23	0.16	0.23	110.48	36.19
1344	KCNK9	0.003720695	0.048518525	-2.83	0.98	3.33	3.49	0.12	2.16	0.19	2.16	354.88	41.03
1345	LYRM9	0.003762879	0.048999573	0.43	0.15	6.57	0.08	0.00	0.05	0.03	0.05	520.61	788.05
1346	OSTF1	0.003763176	0.048999573	-0.33	0.12	7.03	0.04	0.00	0.03	0.03	0.03	1496.30	1070.67
1347	UROD	0.003784112	0.049235591	0.35	0.12	8.12	0.05	0.00	0.04	0.03	0.04	2596.47	3641.12
1348	GALM	0.003787921	0.04924859	0.70	0.24	7.26	0.21	0.00	0.14	0.03	0.14	1039.97	1671.77
1349	RPL5	0.003793339	0.049282475	0.43	0.15	10.10	0.08	0.00	0.05	0.05	0.05	17909.89	27000.05
1350	PDHA1	0.003808075	0.049437282	0.34	0.12	7.46	0.05	0.00	0.03	0.03	0.03	1380.85	1879.58
1351	COMMD6	0.003811963	0.049451125	0.35	0.12	8.17	0.05	0.00	0.03	0.03	0.03	2701.14	3813.86
1352	TRIP6	0.003815051	0.049454577	0.58	0.20	5.97	0.15	0.00	0.09	0.04	0.09	263.67	456.61
1353	BID	0.003825344	0.049504415	-0.41	0.14	5.79	0.04	0.00	0.01	0.04	0.04	456.48	303.42
1354	DGKD	0.003822361	0.049504415	-0.56	0.19	4.98	0.11	0.01	0.05	0.08	0.08	234.56	132.44
1355	GLRX	0.00382737	0.049504415	-0.59	0.20	7.57	0.15	0.00	0.10	0.03	0.10	3455.32	1761.02
1356	GDF11	0.003834671	0.049525749	-1.00	0.35	6.66	0.44	0.01	0.28	0.03	0.28	2644.10	663.67
1357	LRRC9	0.003834068	0.049525749	-1.92	0.66	2.20	1.62	0.21	0.85	0.27	0.85	101.01	6.98
1358	NUDT14	0.003857659	0.049785955	0.62	0.21	5.64	0.17	0.00	0.11	0.05	0.11	181.64	333.18
1359	PDLIM4	0.003863606	0.049826018	1.12	0.39	3.42	0.55	0.14	0.22	0.18	0.22	13.25	43.01

1360	FAT2	0.003871518	0.049891337	-0.91	0.31	3.23	0.32	0.05	0.15	0.19	0.19	55.21	22.74

Supplementary table 4. Sleuth output for genes differentially expressed between ATPase-wildtype and ATPase-mutated tumors

	target_id	pval	qval	b	se_b	mean_obs	var_obs	tech_var	sigma_sq	smooth_sigma_sq	final_sigma_sq	Expression ATPase mutated	Expression ATPase wildtype
1	ENO3	1.73E-23	3.04E-19	-3.13	0.31	5.89	2.84	0.01	0.35	0.05	0.35	2705.47	122.83
2	PNLIP	6.17E-12	5.41E-08	-3.52	0.51	3.18	4.06	0.05	0.90	0.21	0.90	232.69	7.98
3	ARSG	2.57E-10	1.50E-06	-1.14	0.18	5.72	0.45	0.01	0.11	0.05	0.11	634.00	201.45
4	RAB9B	2.38E-09	8.36E-06	-0.77	0.13	5.60	0.19	0.00	0.04	0.06	0.06	433.81	202.44
5	VIM	2.30E-09	8.36E-06	0.77	0.13	8.83	0.21	0.00	0.06	0.05	0.06	4400.24	9616.08
6	P3H2	1.12E-07	0.000325887	-0.57	0.11	6.73	0.12	0.00	0.04	0.04	0.04	1208.08	678.21
7	SLC17A7	1.95E-07	0.000486983	-1.57	0.30	3.64	0.94	0.04	0.29	0.18	0.29	130.42	20.82
8	SYNE3	2.40E-07	0.000526155	0.77	0.15	6.54	0.23	0.00	0.08	0.04	0.08	438.78	993.86
9	CHODL	3.90E-07	0.000760341	-1.55	0.30	2.89	0.92	0.07	0.27	0.23	0.27	49.89	10.97
10	ADCY6	5.78E-07	0.00097564	-0.77	0.16	6.90	0.23	0.00	0.08	0.04	0.08	1605.75	765.00
11	TRIM50	6.12E-07	0.00097564	-1.76	0.35	2.13	1.04	0.17	0.08	0.28	0.28	28.02	3.96
12	PCK1	7.49E-07	0.001094173	-1.82	0.37	1.56	1.31	0.12	0.36	0.31	0.36	17.08	2.35
13	PVALB	8.64E-07	0.001164494	-2.03	0.41	3.24	1.62	0.06	0.55	0.20	0.55	110.47	14.56
14	TTC23L	9.93E-07	0.001242604	-0.65	0.13	5.61	0.15	0.01	0.04	0.05	0.05	410.48	217.09
15	SIGLEC11	1.15E-06	0.001340461	-0.53	0.11	8.48	0.11	0.00	0.04	0.04	0.04	6723.50	3983.41
16	RP11-420B22.2	1.27E-06	0.001387884	2.01	0.41	1.24	1.61	0.17	0.45	0.33	0.45	1.31	7.81
17	ANO4	2.68E-06	0.002759395	1.56	0.33	5.94	1.00	0.01	0.39	0.05	0.39	203.31	815.94
18	GPD1	2.86E-06	0.002777298	-1.28	0.27	4.37	0.68	0.01	0.26	0.13	0.26	197.30	52.03
19	TRIB2	3.01E-06	0.002777298	0.48	0.10	6.95	0.09	0.00	0.03	0.04	0.04	786.99	1283.95
20	ZBTB7C	3.99E-06	0.003497616	0.71	0.15	5.10	0.19	0.01	0.06	0.08	0.08	110.80	222.33
21	SUGCT	4.93E-06	0.004113682	-1.84	0.40	5.66	1.42	0.00	0.58	0.05	0.58	1210.76	159.75
22	ZNF426	7.31E-06	0.005821885	0.76	0.17	6.43	0.25	0.00	0.10	0.04	0.10	419.51	870.72
23	CLDND2	8.55E-06	0.005996037	-0.93	0.21	4.29	0.32	0.02	0.08	0.14	0.14	131.54	52.74
24	SLC46A3	8.12E-06	0.005996037	-0.71	0.16	6.83	0.22	0.00	0.09	0.04	0.09	1436.07	729.69
25	VN1R1	8.41E-06	0.005996037	1.21	0.27	3.72	0.62	0.09	0.17	0.18	0.18	22.85	70.84
26	SLC35F2	9.85E-06	0.006642067	1.78	0.40	3.64	1.36	0.05	0.53	0.18	0.53	16.23	99.61

27	PPP1R16B	1.06E-05	0.006655631	-1.07	0.24	6.51	0.49	0.00	0.21	0.04	0.21	1316.96	497.68
28	SLC16A6	1.03E-05	0.006655631	-1.68	0.38	5.56	1.22	0.01	0.52	0.06	0.52	991.07	150.80
29	MYRF	1.13E-05	0.006813011	-1.00	0.23	4.36	0.43	0.01	0.18	0.13	0.18	152.53	56.91
30	RXFP2	1.22E-05	0.00697899	-4.15	0.95	3.06	7.44	0.08	3.16	0.22	3.16	274.00	63.19
31	SHISA8	1.23E-05	0.00697899	-1.09	0.25	4.89	0.52	0.01	0.22	0.09	0.22	269.42	95.59
32	SCTR	1.28E-05	0.006988156	-1.37	0.31	4.17	0.81	0.04	0.31	0.15	0.31	157.71	45.85
33	KIAA1549L	1.37E-05	0.007293171	-2.26	0.52	6.25	2.22	0.01	0.97	0.04	0.97	2537.48	344.86
34	GLDN	1.48E-05	0.007617903	1.75	0.40	4.47	1.34	0.02	0.57	0.12	0.57	38.74	220.27
35	MYH14	1.54E-05	0.007686631	-1.20	0.28	4.98	0.62	0.02	0.25	0.09	0.25	307.38	107.19
36	LPCAT1	1.72E-05	0.008386583	-0.52	0.12	5.81	0.12	0.00	0.05	0.05	0.05	462.23	279.79
37	C9orf84	2.58E-05	0.011760369	1.89	0.45	6.68	1.60	0.01	0.72	0.04	0.72	279.86	2848.71
38	FCGR3B	2.67E-05	0.011760369	1.06	0.25	3.88	0.49	0.06	0.16	0.16	0.16	29.40	80.40
39	IL1R2	2.64E-05	0.011760369	1.87	0.45	3.92	1.57	0.08	0.63	0.16	0.63	22.30	141.89
40	SLC30A4	2.68E-05	0.011760369	-0.43	0.10	6.72	0.07	0.00	0.03	0.04	0.04	1090.08	697.82
41	PPY	3.09E-05	0.012896822	-2.40	0.58	2.61	2.59	0.14	1.05	0.24	1.05	105.80	7.45
42	REPS2	3.07E-05	0.012896822	-0.46	0.11	8.48	0.09	0.00	0.04	0.04	0.04	6378.33	4097.19
43	SDC1	3.94E-05	0.016067993	-1.29	0.31	6.36	0.75	0.00	0.35	0.04	0.35	1336.11	416.21
44	TXLNGY	4.12E-05	0.016427213	-4.93	1.20	2.89	11.10	0.09	5.12	0.23	5.12	767.49	89.42
45	HRH1	4.35E-05	0.01694485	-0.99	0.24	5.17	0.45	0.01	0.20	0.08	0.20	337.10	131.29
46	SBK1	4.59E-05	0.017489001	-0.63	0.15	5.12	0.14	0.01	0.04	0.08	0.08	248.26	131.85
47	DLGAP1	4.92E-05	0.01789195	1.53	0.38	5.46	1.08	0.03	0.48	0.06	0.48	102.76	566.19
48	LTBP2	5.00E-05	0.01789195	-0.50	0.12	5.94	0.11	0.01	0.05	0.05	0.05	531.24	318.13
49	MYBPHL	4.92E-05	0.01789195	-3.07	0.76	3.82	4.35	0.05	2.02	0.17	2.02	317.79	36.07
50	THSD4	5.12E-05	0.017941689	-0.45	0.11	7.23	0.09	0.00	0.04	0.04	0.04	1821.89	1180.18
51	XKRX	5.71E-05	0.019613918	1.65	0.41	4.00	1.26	0.05	0.56	0.16	0.56	26.44	138.52
52	ESM1	6.15E-05	0.020743293	-1.50	0.37	5.29	1.04	0.00	0.50	0.07	0.50	493.57	157.62
53	ALOX15B	7.60E-05	0.025120694	1.70	0.43	4.09	1.36	0.06	0.61	0.15	0.61	23.27	162.38
54	DDX3Y	8.03E-05	0.026067827	-4.97	1.26	2.95	11.65	0.07	5.64	0.22	5.64	896.95	117.77
55	UTY	8.21E-05	0.026149161	-4.64	1.18	4.29	10.19	0.22	4.79	0.14	4.79	2609.51	377.98

56	KCNMB4	8.90E-05	0.027865326	1.47	0.37	6.26	1.03	0.01	0.50	0.04	0.50	227.43	1293.42
57	KDM5D	0.000108252	0.033284697	-3.60	0.93	5.54	6.22	0.22	2.89	0.06	2.89	3795.97	685.90
58	IDH2	0.000116463	0.034322039	-0.75	0.19	6.14	0.27	0.00	0.13	0.04	0.13	802.11	355.70
59	LAMP5	0.000115671	0.034322039	3.75	0.97	4.95	6.78	0.14	3.26	0.09	3.26	83.50	1963.88
60	SYT16	0.000117501	0.034322039	1.85	0.48	1.92	1.65	0.05	0.77	0.29	0.77	1.88	22.67
61	WHRN	0.000121202	0.034822827	-0.51	0.13	8.13	0.12	0.00	0.06	0.04	0.06	4747.90	2849.57
62	MTHFD2	0.000131783	0.037252211	0.63	0.16	5.29	0.19	0.01	0.09	0.07	0.09	139.91	268.40
63	PITPNC1	0.000152672	0.042471923	-0.62	0.16	6.40	0.19	0.00	0.09	0.04	0.09	904.21	492.19
64	ANKDD1A	0.0001601	0.042513913	0.51	0.14	6.43	0.13	0.00	0.06	0.04	0.06	466.17	783.00
65	FIBCD1	0.000158385	0.042513913	1.17	0.31	5.23	0.68	0.03	0.32	0.07	0.32	97.31	374.20
66	TES	0.000158279	0.042513913	0.59	0.15	5.17	0.17	0.01	0.08	0.08	0.08	129.55	229.98
67	CKS1B	0.000170093	0.044493241	-0.40	0.11	6.41	0.08	0.00	0.04	0.04	0.04	778.88	528.58
68	PHOSPHO1	0.000172803	0.044537335	1.46	0.39	2.74	1.05	0.12	0.43	0.24	0.43	8.50	31.31
69	DNAJB5	0.000176242	0.0447655	-0.65	0.17	6.36	0.21	0.00	0.11	0.04	0.11	904.79	464.88
70	HOXB3	0.000183157	0.044887072	1.09	0.29	4.70	0.58	0.04	0.26	0.10	0.26	64.63	192.16
71	SCN9A	0.000184404	0.044887072	-1.60	0.43	3.57	1.26	0.03	0.62	0.18	0.62	108.90	28.44
72	SYDE2	0.000184252	0.044887072	0.49	0.13	6.40	0.12	0.00	0.06	0.04	0.06	458.01	751.52
73	WBP1	0.000191134	0.045887976	-0.39	0.10	7.16	0.08	0.00	0.04	0.04	0.04	1632.83	1117.16
74	CPT1B	0.000206539	0.048264093	-0.80	0.21	5.26	0.32	0.00	0.16	0.07	0.16	320.33	154.53
75	RANBP17	0.000206125	0.048264093	-0.43	0.12	6.54	0.09	0.00	0.05	0.04	0.05	906.59	597.39

Supplementary table 5. Sleuth output for genes differentially expressed between KCNJ5-wildtype and KCNJ5-mutated tumors

	target_id	pval	qval	b	se_b	mean_obs	var_obs	tech_var	sigma_sq	smooth_sigma_sq	final_sigma_sq	Expression KCNJ5 mutated	Expression KCNJ5 wildtype
1	AMOT	4.04E-14	7.07E-10	1.39	0.18	6.64	0.56	0.00	0.11	0.04	0.11	319.04	1277.77
2	MAP9	5.12E-11	3.78E-07	1.56	0.24	5.43	0.75	0.00	0.18	0.06	0.18	97.64	400.48
3	PTPRZ1	6.48E-11	3.78E-07	2.59	0.40	3.81	2.08	0.04	0.48	0.17	0.48	8.73	140.69
4	CHRNA3	1.34E-10	5.86E-07	3.06	0.48	2.96	2.93	0.04	0.72	0.22	0.72	2.20	81.19
5	SALL4	5.72E-09	2.00E-05	1.73	0.30	2.86	0.98	0.05	0.25	0.23	0.25	5.84	34.62
6	PPP2R2B	1.12E-08	3.27E-05	-1.59	0.28	4.18	0.84	0.02	0.24	0.15	0.24	199.66	42.90
7	SIAH3	2.16E-08	5.41E-05	1.50	0.27	4.05	0.76	0.02	0.22	0.15	0.22	21.63	110.34
8	FAM153B	3.91E-08	8.29E-05	-1.06	0.19	5.20	0.38	0.01	0.11	0.08	0.11	375.76	135.17
9	TPSB2	4.26E-08	8.29E-05	2.85	0.52	3.04	2.77	0.15	0.75	0.22	0.75	3.45	85.27
10	SLC8A1	8.22E-08	0.000143987	1.43	0.27	7.43	0.70	0.00	0.23	0.04	0.23	666.59	3135.79
11	HAS2	1.34E-07	0.000213909	-2.35	0.45	4.42	1.93	0.03	0.63	0.13	0.63	515.68	52.21
12	CHRM3	1.96E-07	0.000286806	1.36	0.26	3.35	0.60	0.03	0.14	0.20	0.20	12.94	46.01
13	LRRC10B	2.68E-07	0.000360656	2.01	0.39	1.76	1.35	0.20	0.22	0.31	0.31	1.25	13.25
14	RASSF5	5.59E-07	0.000699339	-0.73	0.15	7.14	0.19	0.00	0.07	0.04	0.07	2071.62	1026.04
15	CLDN11	6.00E-07	0.000700918	-1.38	0.28	4.59	0.69	0.01	0.24	0.11	0.24	297.89	64.46
16	DMKN	6.57E-07	0.000713235	2.34	0.47	4.71	1.98	0.02	0.71	0.10	0.71	23.35	350.23
17	STEAP1	6.92E-07	0.000713235	-1.71	0.34	3.53	1.06	0.05	0.35	0.19	0.35	114.00	23.98
18	SH3BGRL2	7.85E-07	0.000764585	0.56	0.11	6.96	0.11	0.00	0.04	0.04	0.04	745.10	1295.38
19	LMCD1	1.96E-06	0.001811782	-1.05	0.22	5.54	0.41	0.01	0.15	0.06	0.15	517.85	198.08
20	CHST2	2.18E-06	0.001892659	0.90	0.19	4.63	0.30	0.01	0.10	0.11	0.11	57.01	146.84
21	RASD2	2.38E-06	0.001892659	2.00	0.42	4.14	1.50	0.03	0.57	0.15	0.57	16.77	197.65
22	SETDB2	2.33E-06	0.001892659	-0.52	0.11	7.59	0.09	0.00	0.03	0.04	0.04	2823.71	1697.87
23	EFNB2	2.71E-06	0.002065205	0.61	0.13	6.49	0.14	0.00	0.05	0.04	0.05	449.47	826.48
24	C10orf82	3.58E-06	0.00254052	3.11	0.67	4.25	3.70	0.04	1.46	0.14	1.46	8.44	411.21
25	FAM153A	3.62E-06	0.00254052	-1.18	0.26	3.70	0.45	0.04	0.08	0.17	0.17	90.47	28.77

26	FAM189A2	4.34E-06	0.00281441	0.77	0.17	5.78	0.23	0.00	0.09	0.05	0.09	197.77	436.26
27	KIAA1644	4.26E-06	0.00281441	3.15	0.69	5.01	3.82	0.03	1.54	0.09	1.54	22.76	730.07
28	IL12RB2	5.03E-06	0.003148705	1.60	0.35	4.23	0.99	0.07	0.34	0.14	0.34	24.21	146.77
29	CD28	5.27E-06	0.003182732	1.84	0.40	4.02	1.31	0.08	0.47	0.16	0.47	16.06	139.51
30	RPL22L1	6.15E-06	0.003591668	-0.54	0.12	6.10	0.10	0.00	0.03	0.04	0.04	656.70	376.08
31	NSUN7	7.50E-06	0.004238923	1.27	0.28	3.68	0.64	0.02	0.25	0.18	0.25	20.69	63.19
32	FRK	7.99E-06	0.004244105	0.55	0.12	8.04	0.12	0.00	0.05	0.04	0.05	2148.73	3828.15
33	RPS4X	7.78E-06	0.004244105	-0.52	0.12	9.00	0.11	0.00	0.04	0.05	0.05	11550.80	6965.83
34	ITM2C	9.58E-06	0.004939834	1.19	0.27	6.19	0.56	0.00	0.24	0.04	0.24	243.45	788.72
35	LRFN2	1.02E-05	0.005092973	2.73	0.62	3.23	2.95	0.09	1.18	0.20	1.18	4.16	116.66
36	CPEB1	1.10E-05	0.005355567	-1.24	0.28	5.31	0.62	0.04	0.23	0.07	0.23	471.18	154.45
37	B3GALT2	1.26E-05	0.005801222	-0.83	0.19	4.67	0.27	0.01	0.10	0.11	0.11	192.82	85.70
38	POSTN	1.23E-05	0.005801222	1.79	0.41	6.56	1.27	0.00	0.55	0.04	0.55	262.46	1561.47
39	ALDH4A1	1.30E-05	0.005838448	1.29	0.30	7.59	0.67	0.00	0.29	0.04	0.29	871.30	3536.87
40	METTL7B	1.87E-05	0.008202517	2.42	0.57	4.52	2.38	0.02	1.04	0.12	1.04	26.36	379.20
41	NRP1	2.33E-05	0.009940182	0.51	0.12	8.72	0.11	0.00	0.05	0.04	0.05	4482.16	7414.43
42	APLNR	2.75E-05	0.010947654	1.63	0.39	4.69	1.10	0.01	0.50	0.11	0.50	39.50	246.81
43	ARHGEF2	2.69E-05	0.010947654	0.85	0.20	5.63	0.30	0.01	0.13	0.06	0.13	161.05	406.49
44	TBC1D1	2.62E-05	0.010947654	0.54	0.13	5.83	0.09	0.00	0.02	0.05	0.05	238.12	412.20
45	HELQ	3.04E-05	0.01166218	-0.45	0.11	7.33	0.08	0.00	0.03	0.04	0.04	2080.66	1330.23
46	PCK1	3.06E-05	0.01166218	1.77	0.42	1.56	1.31	0.12	0.48	0.33	0.48	1.06	11.83
47	GNG11	3.22E-05	0.01197512	-1.10	0.26	6.93	0.50	0.00	0.23	0.04	0.23	2299.82	806.56
48	PDE4D	3.28E-05	0.01197512	1.54	0.37	4.06	0.99	0.02	0.43	0.15	0.43	20.44	133.81
49	REPS2	3.38E-05	0.012078218	0.47	0.11	8.48	0.09	0.00	0.04	0.04	0.04	3559.43	5734.75
50	RMND1	3.92E-05	0.013731575	-0.52	0.13	6.61	0.12	0.00	0.05	0.04	0.05	1059.91	644.14
51	ABCB4	4.16E-05	0.013783457	0.76	0.19	7.70	0.25	0.00	0.12	0.04	0.12	1366.13	3047.06
52	ALDH1A1	4.20E-05	0.013783457	-1.34	0.33	7.80	0.76	0.00	0.36	0.04	0.36	6121.61	1946.94
53	ESM1	4.25E-05	0.013783457	1.57	0.38	5.29	1.04	0.00	0.49	0.07	0.49	81.21	397.39
54	SLC4A11	4.11E-05	0.013783457	-1.43	0.35	4.99	0.87	0.03	0.38	0.09	0.38	391.26	118.78

55	TLE4	4.44E-05	0.01415546	0.50	0.12	5.98	0.09	0.00	0.03	0.05	0.05	285.92	471.01
56	CTNNA3	4.66E-05	0.014595702	1.66	0.41	2.87	1.18	0.11	0.44	0.23	0.44	5.67	43.42
57	CHRNB4	4.84E-05	0.014837563	1.78	0.44	4.37	1.35	0.01	0.62	0.13	0.62	24.70	200.80
58	PROCR	4.91E-05	0.014837563	-0.74	0.18	5.09	0.23	0.01	0.10	0.08	0.10	279.91	131.91
59	CYBRD1	5.41E-05	0.01608301	-0.79	0.20	7.51	0.27	0.00	0.13	0.04	0.13	3243.69	1482.16
60	CD163L1	5.61E-05	0.016086113	1.65	0.41	3.56	1.17	0.05	0.52	0.18	0.52	13.79	76.31
61	DPY19L2P4	5.60E-05	0.016086113	-1.08	0.27	3.34	0.47	0.04	0.17	0.20	0.20	64.93	20.99
62	SNCA	5.69E-05	0.016086113	-1.34	0.33	5.20	0.77	0.02	0.34	0.08	0.34	473.44	139.22
63	GJC1	5.84E-05	0.01625789	1.06	0.26	4.43	0.48	0.02	0.21	0.13	0.21	41.98	138.81
64	UNC79	6.34E-05	0.0173633	2.01	0.50	5.16	1.75	0.01	0.83	0.08	0.83	67.59	499.64
65	NEBL	6.47E-05	0.017431924	-0.95	0.24	3.90	0.39	0.01	0.18	0.16	0.18	95.39	39.55
66	SLC19A1	6.56E-05	0.017431924	0.76	0.19	4.74	0.22	0.02	0.07	0.10	0.10	68.54	156.00
67	GBGT1	6.77E-05	0.017442092	-0.53	0.13	6.09	0.12	0.00	0.05	0.04	0.05	630.95	382.67
68	PCOLCE2	6.69E-05	0.017442092	-1.45	0.36	3.23	0.91	0.13	0.31	0.20	0.31	78.56	18.05
69	RIMKLA	7.42E-05	0.018851168	-1.49	0.38	4.53	0.96	0.07	0.40	0.12	0.40	303.84	69.82
70	DPP4	8.00E-05	0.020034984	2.09	0.53	4.90	1.90	0.01	0.92	0.09	0.92	61.65	336.23
71	RASGEF1A	8.16E-05	0.020148318	1.99	0.51	4.30	1.73	0.07	0.78	0.14	0.78	20.54	217.77
72	SCN2A	8.50E-05	0.020682164	2.03	0.52	4.10	1.81	0.08	0.81	0.15	0.81	20.02	172.15
73	RGS7	9.31E-05	0.022355217	-2.02	0.52	4.07	1.80	0.06	0.84	0.15	0.84	246.15	49.16
74	WDR63	9.61E-05	0.022771906	-1.64	0.42	7.47	1.18	0.00	0.58	0.04	0.58	5771.54	1379.02
75	CPA3	0.000102014	0.023728678	1.50	0.38	3.76	0.99	0.03	0.46	0.17	0.46	16.45	91.20
76	MYLIP	0.000102897	0.023728678	-0.73	0.19	7.18	0.23	0.00	0.12	0.04	0.12	2275.39	1067.96
77	SOWAHB	0.000106011	0.024129109	1.75	0.45	5.65	1.36	0.01	0.67	0.05	0.67	118.79	657.02
78	APOBEC3G	0.000113896	0.025267578	-0.55	0.14	5.75	0.13	0.00	0.06	0.05	0.06	469.29	266.95
79	RBP5	0.000113532	0.025267578	0.82	0.21	4.33	0.30	0.01	0.14	0.13	0.14	48.03	104.57
80	EMID1	0.000124299	0.026921656	0.66	0.17	6.26	0.20	0.00	0.10	0.04	0.10	356.67	671.15
81	LRFN5	0.000124876	0.026921656	0.84	0.22	5.80	0.31	0.02	0.14	0.05	0.14	195.36	470.36
82	OTOA	0.000127164	0.026921656	1.89	0.49	5.14	1.61	0.02	0.80	0.08	0.80	53.07	456.97
83	TUSC3	0.000127496	0.026921656	-0.45	0.12	8.26	0.09	0.00	0.05	0.04	0.05	5296.81	3378.38

84	NRTN	0.000129123	0.026940494	1.14	0.30	4.41	0.59	0.01	0.28	0.13	0.28	39.90	141.47
85	ANXA6	0.000132326	0.02728413	-0.44	0.12	8.41	0.09	0.00	0.04	0.04	0.04	6151.91	3970.84
86	RP5-1052I5.2	0.000134081	0.027324435	-0.81	0.21	4.49	0.21	0.03	0.03	0.12	0.12	157.52	69.71
87	EIF2S3	0.000142996	0.028300525	-0.40	0.11	6.80	0.07	0.00	0.04	0.04	0.04	1186.34	800.82
88	FAM175A	0.0001424	0.028300525	-0.49	0.13	6.23	0.11	0.00	0.05	0.04	0.05	710.08	445.57
89	SRPX	0.000143715	0.028300525	-1.34	0.35	7.31	0.81	0.00	0.41	0.04	0.41	3873.59	1231.65
90	GDAP1L1	0.000154347	0.030056417	2.03	0.54	5.06	1.87	0.03	0.93	0.08	0.93	43.25	459.11
91	USP53	0.000162657	0.031326694	-0.63	0.17	7.30	0.18	0.00	0.09	0.04	0.09	2360.25	1250.11
92	SLC22A17	0.000170013	0.032387542	1.05	0.28	6.67	0.51	0.00	0.26	0.04	0.26	406.13	1262.27
93	DCN	0.000180665	0.034046531	-1.26	0.34	9.17	0.73	0.00	0.38	0.05	0.38	23936.41	7630.15
94	ABCA6	0.000202498	0.03658741	-0.53	0.14	5.82	0.13	0.00	0.06	0.05	0.06	491.20	292.65
95	EEF1B2	0.000201252	0.03658741	-0.42	0.11	8.74	0.07	0.00	0.03	0.04	0.04	8292.01	5495.96
96	FAM20C	0.000197032	0.03658741	0.66	0.18	5.66	0.20	0.00	0.10	0.05	0.10	186.87	380.66
97	SCN9A	0.000200576	0.03658741	1.65	0.44	3.57	1.26	0.03	0.63	0.18	0.63	12.60	84.64
98	TPSAB1	0.000211691	0.037858178	3.01	0.81	2.45	4.20	0.21	1.99	0.26	1.99	1.54	62.35
99	ADAM8	0.000215912	0.038001611	1.14	0.31	3.15	0.60	0.06	0.26	0.21	0.26	11.32	39.21
100	HPSE	0.00021683	0.038001611	-1.11	0.30	5.31	0.58	0.01	0.29	0.07	0.29	434.63	163.02
101	EVX1	0.000245148	0.042539292	2.01	0.55	4.17	1.89	0.03	0.97	0.15	0.97	31.90	174.38
102	IFRD1	0.00025597	0.043981652	-0.43	0.12	6.07	0.08	0.00	0.04	0.04	0.04	588.62	381.11
103	KIAA1024	0.000270419	0.046013294	-1.65	0.45	6.47	1.28	0.00	0.68	0.04	0.68	2401.08	510.06
104	IDH3A	0.000276527	0.046519535	0.39	0.11	7.09	0.07	0.00	0.03	0.04	0.04	930.00	1385.96
105	SYT7	0.000278703	0.046519535	2.01	0.55	3.84	1.91	0.06	0.96	0.17	0.96	12.53	161.55
106	AC007325.2	0.000294594	0.048707998	-2.71	0.75	1.66	3.47	0.26	1.61	0.32	1.61	36.15	5.92

Supplementary table 6. Sleuth output for genes differentially expressed between ATPase-/CACNA1D-mutated tumors and KCNJ5-mutated tumors

	target_id	pval	qval	b	se_b	mean_obs	var_obs	tech_var	sigma_sq	smooth_sigma_sq	final_sigma_sq	Expression KCNJ5 mutated	Expression CACNA1D/ATPase mutated
1	KCNJ8	2.99E-10	5.27E-06	1.22	0.19	6.82	0.49	0.00	0.11	0.02	0.11	494.60	1549.42
2	CD99	7.37E-09	6.49E-05	0.52	0.09	7.46	0.09	0.00	0.02	0.02	0.02	1279.98	2178.60
3	NLGN4Y	1.06E-07	0.000620359	5.84	1.10	4.29	12.26	0.15	3.37	0.09	3.37	2.33	1804.65
4	USP9Y	2.03E-07	0.000894978	5.56	1.07	4.07	11.22	0.13	3.20	0.10	3.20	1.97	1242.08
5	ANOS2P	7.29E-07	0.001426522	3.82	0.77	1.54	5.45	0.01	1.72	0.33	1.72	0.00	37.62
6	DDX3Y	5.65E-07	0.001426522	5.78	1.15	3.57	12.38	0.05	3.84	0.13	3.84	0.77	880.50
7	HSFY2	7.16E-07	0.001426522	3.28	0.66	1.22	4.01	0.03	1.25	0.38	1.25	0.00	19.55
8	TMSB4Y	5.99E-07	0.001426522	4.74	0.95	2.07	8.35	0.01	2.62	0.25	2.62	0.00	108.38
9	ZFY	4.64E-07	0.001426522	5.28	1.05	3.45	10.30	0.37	2.83	0.13	2.83	1.92	549.88
10	LRRC8B	8.83E-07	0.001555626	0.46	0.09	8.10	0.08	0.00	0.02	0.02	0.02	2524.20	4031.16
11	SRY	1.45E-06	0.002127062	3.35	0.70	1.26	4.27	0.03	1.38	0.37	1.38	0.00	22.76
12	UTY	1.36E-06	0.002127062	5.23	1.08	4.93	10.36	0.18	3.24	0.06	3.24	6.39	2601.85
13	KDM5D	1.59E-06	0.002153785	4.20	0.88	5.96	6.72	0.21	2.03	0.03	2.03	36.03	3917.83
14	B3GALT2	2.58E-06	0.003249089	-0.89	0.19	4.71	0.31	0.01	0.09	0.07	0.09	191.83	80.09
15	NTS	3.07E-06	0.003606078	-4.13	0.88	2.28	6.59	0.18	2.11	0.23	2.11	298.31	3.61
16	RPS4Y1	4.24E-06	0.004671358	7.11	1.55	4.97	19.77	0.10	6.88	0.05	6.88	2.64	8475.67
17	PRKAA2	4.60E-06	0.004763241	-0.79	0.17	6.20	0.24	0.00	0.08	0.03	0.08	807.53	366.30
18	PTH1R	5.65E-06	0.005261501	-0.62	0.14	6.28	0.15	0.01	0.05	0.03	0.05	794.80	419.59
19	TXLNGY	5.68E-06	0.005261501	5.57	1.23	3.43	12.20	0.06	4.32	0.13	4.32	0.88	739.02
20	RP11-108M9.5	6.41E-06	0.005640621	1.00	0.22	5.01	0.39	0.01	0.13	0.05	0.13	94.33	232.79
21	LRRC73	1.29E-05	0.0107936	0.55	0.13	5.54	0.12	0.00	0.04	0.04	0.04	189.18	327.02
22	EIF1AY	2.11E-05	0.01618703	4.88	1.15	3.49	9.83	0.06	3.79	0.13	3.79	1.45	673.31
23	SHROOM2P1	2.03E-05	0.01618703	3.34	0.78	1.26	4.59	0.04	1.76	0.37	1.76	0.00	27.88
24	PHKA2	2.25E-05	0.016501102	0.48	0.11	6.25	0.10	0.00	0.04	0.03	0.04	394.06	650.49
25	IL17RD	2.40E-05	0.016921424	-1.20	0.28	3.13	0.51	0.08	0.06	0.15	0.15	46.79	14.81
26	ARHGEF2	2.90E-05	0.019650966	0.68	0.16	5.46	0.19	0.01	0.07	0.04	0.07	162.32	319.90
27	SPINK13	3.11E-05	0.02026056	-0.94	0.23	3.69	0.32	0.03	0.07	0.12	0.12	71.09	27.87

28	S1PR3	3.32E-05	0.020905736	0.69	0.17	8.77	0.20	0.00	0.08	0.03	0.08	4444.63	8864.03
29	ZNF676	3.86E-05	0.023459197	-1.00	0.24	3.57	0.35	0.04	0.05	0.13	0.13	63.54	24.37
30	STEAP2	4.25E-05	0.024966256	-1.17	0.29	5.15	0.58	0.01	0.22	0.05	0.22	374.46	115.78
31	RP5-1052I5.2	4.84E-05	0.027515866	-0.79	0.19	4.45	0.26	0.03	0.08	0.08	0.08	144.24	63.04
32	DNAH10	5.29E-05	0.029109054	1.35	0.33	3.68	0.69	0.20	0.03	0.12	0.12	20.40	72.63
33	EFHB	6.46E-05	0.03447216	-1.28	0.32	3.92	0.66	0.19	0.05	0.11	0.11	108.34	34.84
34	DPY19L2P4	7.59E-05	0.039330031	-1.07	0.27	3.43	0.50	0.04	0.17	0.13	0.17	63.86	21.01
35	GCHFR	8.05E-05	0.040520698	-0.85	0.22	4.31	0.31	0.03	0.11	0.09	0.11	129.31	55.16
36	ZNF541	8.68E-05	0.042469273	-1.08	0.28	4.33	0.51	0.07	0.16	0.09	0.16	153.52	52.86
37	RFK	9.39E-05	0.04468092	0.40	0.10	6.45	0.07	0.00	0.03	0.02	0.03	504.16	759.49
38	CTRB2	0.000100433	0.046545444	1.74	0.45	2.07	1.33	0.25	0.34	0.26	0.34	2.94	20.10
39	CPNE4	0.000104672	0.047265992	1.64	0.42	4.04	1.18	0.08	0.44	0.10	0.44	26.85	131.70
40	RRP12	0.000109824	0.048352892	0.41	0.11	5.78	0.07	0.00	0.03	0.03	0.03	256.75	391.87
